# Breast and Prostate Cancer Risks for Male *BRCA1* and* BRCA2* Pathogenic Variant Carriers Using Polygenic Risk Scores

**DOI:** 10.1093/jnci/djab147

**Published:** 2021-07-28

**Authors:** Daniel R Barnes, Valentina Silvestri, Goska Leslie, Lesley McGuffog, Joe Dennis, Xin Yang, Julian Adlard, Bjarni A Agnarsson, Munaza Ahmed, Kristiina Aittomäki, Irene L Andrulis, Adalgeir Arason, Norbert Arnold, Bernd Auber, Jacopo Azzollini, Judith Balmaña, Rosa B Barkardottir, Daniel Barrowdale, Julian Barwell, Muriel Belotti, Javier Benitez, Pascaline Berthet, Susanne E Boonen, Åke Borg, Aniko Bozsik, Angela F Brady, Paul Brennan, Carole Brewer, Joan Brunet, Agostino Bucalo, Saundra S Buys, Trinidad Caldés, Maria A Caligo, Ian Campbell, Hayley Cassingham, Lise Lotte Christensen, Giulia Cini, Kathleen B M Claes, GEMO Study Collaborators, EMBRACE Collaborators, Jackie Cook, Anna Coppa, Laura Cortesi, Giuseppe Damante, Esther Darder, Rosemarie Davidson, Miguel de la Hoya, Kim De Leeneer, Robin de Putter, Jesús Del Valle, Orland Diez, Yuan Chun Ding, Susan M Domchek, Alan Donaldson, Jacqueline Eason, Ros Eeles, Christoph Engel, D Gareth Evans, Lidia Feliubadaló, Florentia Fostira, Megan Frone, Debra Frost, David Gallagher, Andrea Gehrig, Sophie Giraud, Gord Glendon, Andrew K Godwin, David E Goldgar, Mark H Greene, Helen Gregory, Eva Gross, Eric Hahnen, Ute Hamann, Thomas V O Hansen, Helen Hanson, Julia Hentschel, Judit Horvath, KConFab Investigators, HEBON Investigators, Louise Izatt, Angel Izquierdo, Paul A James, Ramunas Janavicius, Uffe Birk Jensen, Oskar Th Johannsson, Esther M John, Gero Kramer, Lone Kroeldrup, Torben A Kruse, Charlotte Lautrup, Conxi Lazaro, Fabienne Lesueur, Adria Lopez-Fernández, Phuong L Mai, Siranoush Manoukian, Zoltan Matrai, Laura Matricardi, Kara N Maxwell, Noura Mebirouk, Alfons Meindl, Marco Montagna, Alvaro N Monteiro, Patrick J Morrison, Taru A Muranen, Alex Murray, Katherine L Nathanson, Susan L Neuhausen, Heli Nevanlinna, Tu Nguyen-Dumont, Dieter Niederacher, Edith Olah, Olufunmilayo I Olopade, Domenico Palli, Michael T Parsons, Inge Sokilde Pedersen, Bernard Peissel, Pedro Perez-Segura, Paolo Peterlongo, Annabeth H Petersen, Pedro Pinto, Mary E Porteous, Caroline Pottinger, Miquel Angel Pujana, Paolo Radice, Juliane Ramser, Johanna Rantala, Mark Robson, Mark T Rogers, Karina Rønlund, Andreas Rump, Ana María Sánchez de Abajo, Payal D Shah, Saba Sharif, Lucy E Side, Christian F Singer, Zsofia Stadler, Linda Steele, Dominique Stoppa-Lyonnet, Christian Sutter, Yen Yen Tan, Manuel R Teixeira, Alex Teulé, Darcy L Thull, Marc Tischkowitz, Amanda E Toland, Stefania Tommasi, Angela Toss, Alison H Trainer, Vishakha Tripathi, Virginia Valentini, Christi J van Asperen, Marta Venturelli, Alessandra Viel, Joseph Vijai, Lisa Walker, Shan Wang-Gohrke, Barbara Wappenschmidt, Anna Whaite, Ines Zanna, Kenneth Offit, Mads Thomassen, Fergus J Couch, Rita K Schmutzler, Jacques Simard, Douglas F Easton, Georgia Chenevix-Trench, Antonis C Antoniou, Laura Ottini, the Consortium of Investigators of Modifiers of BRCA1 and BRCA2

**Affiliations:** 1 Centre for Cancer Genetic Epidemiology, Department of Public Health and Primary Care, University of Cambridge, Cambridge, UK; 2 Department of Molecular Medicine, Sapienza University of Rome, Rome, Italy; 3 Yorkshire Regional Genetics Service, Chapel Allerton Hospital, Leeds, UK; 4 Department of Pathology, Landspitali University Hospital, Reykjavik, Iceland; 5 School of Medicine, University of Iceland, Reykjavik, Iceland; 6 North East Thames Regional Genetics Service, Great Ormond Street Hospital for Children NHS Trust, London, UK; 7 Department of Clinical Genetics, Helsinki University Hospital, University of Helsinki, Helsinki, Finland; 8 Fred A. Litwin Center for Cancer Genetics, Lunenfeld-Tanenbaum Research Institute of Mount Sinai Hospital, Toronto, ON, Canada; 9 Department of Molecular Genetics, University of Toronto, Toronto, ON, Canada; 10 BMC (Biomedical Centre), Faculty of Medicine, University of Iceland, Reykjavik, Iceland; 11 Department of Gynaecology and Obstetrics, University Hospital of Schleswig-Holstein, Campus Kiel, Christian-Albrechts University Kiel, Kiel, Germany; 12 Institute of Clinical Molecular Biology, University Hospital of Schleswig-Holstein, Campus Kiel, Christian-Albrechts University Kiel, Kiel, Germany; 13 Department of Human Genetics, Hannover Medical School, Hannover, Germany; 14 Unit of Medical Genetics, Department of Medical Oncology and Hematology, Fondazione IRCCS Istituto Nazionale dei Tumori di Milano, Milan, Italy; 15 Hereditary Cancer Genetics Group, Vall d’Hebron Institute of Oncology, Vall d’Hebron Hospital Campus, Barcelona, Spain; 16 Department of Medical Oncology, Vall d’Hebron Hospital Universitari, Vall d’Hebron Barcelona Hospital Campus, Barcelona, Spain; 17 Leicestershire Clinical Genetics Service, University Hospitals of Leicester NHS Trust, Leicester, UK; 18 Service de Génétique, Institut Curie, Paris, France; 19 Biomedical Network on Rare Diseases (CIBERER), Madrid, Spain; 20 Human Cancer Genetics Programme, Spanish National Cancer Research Centre (CNIO), Madrid, Spain; 21 Département de Biopathologie, Centre François Baclesse, Caen, France; 22 Department of Clinical Genetics, Odense University Hospital, Odense, Denmark; 23 Division of Oncology and Pathology, Department of Clinical Sciences Lund, Lund University, Lund, Sweden; 24 Department of Molecular Genetics, National Institute of Oncology, Budapest, Hungary; 25 North West Thames Regional Genetics Service, London North West University Healthcare NHS Trust, Northwick Park Hospital, Harrow, UK; 26 Northern Genetics Service, Newcastle Hospitals NHS Foundation Trust, Newcastle, UK; 27 Department of Clinical Genetics, Royal Devon & Exeter Hospital, Exeter, UK; 28 Hereditary Cancer Program, Oncobell-IDIBELL-IGTP, Catalan Institute of Oncology, CIBERONC, Barcelona, Spain; 29 Department of Internal Medicine, Huntsman Cancer Institute at the University of Utah, Salt Lake City, UT, USA; 30 Molecular Oncology Laboratory, CIBERONC, Hospital Clinico San Carlos, IdISSC (Instituto de Investigación Sanitaria del Hospital Clínico San Carlos), Madrid, Spain; 31 SOD Genetica Molecolare, University Hospital, Pisa, Italy; 32 Peter MacCallum Cancer Center, Melbourne, Victoria, Australia; 33 Sir Peter MacCallum Department of Oncology, The University of Melbourne, Melbourne, Victoria, Australia; 34 Department of Internal Medicine, Division of Human Genetics, The Ohio State University Wexner Medical Center, Columbus, OH, USA; 35 Division of Surgical Oncology, National Cancer Centre, Singapore, Singapore; 36 Division of Functional Onco-Genomics and Genetics, Centro di Riferimento Oncologico di Aviano (CRO), IRCCS, Aviano, Italy; 37 Centre for Medical Genetics, Ghent University, Gent, Belgium; 38 Department of Tumour Biology, INSERM U830, Paris, France; 39 Institut Curie, Paris, France; 40 Mines ParisTech, Fontainebleau, France; 41 Sheffield Clinical Genetics Service, Sheffield Children’s Hospital, Sheffield, UK; 42 Department of Experimental Medicine, Sapienza University of Rome, Rome, Italy; 43 Department of Oncology and Haematology, University of Modena and Reggio Emilia, Modena, Italy; 44 Department of Medicine, University of Udine, Udine, Italy; 45 Department of Clinical Genetics, South Glasgow University Hospitals, Glasgow, UK; 46 Area of Clinical and Molecular Genetics, Vall d’Hebron Hospital Universitari, Vall d’Hebron Barcelona Hospital Campus, Barcelona, Spain; 47 Department of Population Sciences, Beckman Research Institute of City of Hope, Duarte, CA, USA; 48 Basser Center for BRCA, Abramson Cancer Center, University of Pennsylvania, Philadelphia, PA, USA; 49 Clinical Genetics Department, St Michael’s Hospital, Bristol, UK; 50 Nottingham Clinical Genetics Service, Nottingham University Hospitals NHS Trust, Nottingham, UK; 51 Oncogenetics Team, The Institute of Cancer Research and Royal Marsden NHS Foundation Trust, London, UK; 52 Institute for Medical Informatics, Statistics and Epidemiology, University of Leipzig, Leipzig, Germany; 53 LIFE—Leipzig Research Centre for Civilization Diseases, University of Leipzig, Leipzig, Germany; 54 Division of Evolution and Genomic Sciences, School of Biological Sciences, Faculty of Biology, Medicine and Health, University of Manchester, Manchester Academic Health Science Centre, Manchester, UK; 55 North West Genomics Laboratory Hub, Manchester Centre for Genomic Medicine, St Mary’s Hospital, Manchester University NHS Foundation Trust, Manchester Academic Health Science Centre, Manchester, UK; 56 Molecular Diagnostics Laboratory, INRASTES, National Centre for Scientific Research ‘Demokritos’, Athens, Greece; 57 Clinical Genetics Branch, Division of Cancer Epidemiology and Genetics, National Cancer Institute, Bethesda, MD, USA; 58 Academic Unit of Clinical and Molecular Oncology, Trinity College Dublin and St James’s Hospital, Dublin, Eire; 59 Department of Human Genetics, University Würzburg, Würzburg, Germany; 60 Service de Génétique, Groupement Hospitalier Est, Hospices Civils de Lyon, Bron, France; 61 Department of Pathology and Laboratory Medicine, University of Kansas, Medical Center, Kansas City, KS, USA; 62 Department of Dermatology, Huntsman Cancer Institute, University of Utah School of Medicine, Salt Lake City, UT, USA; 63 North of Scotland Regional Genetics Service, NHS Grampian & University of Aberdeen, Foresterhill, Aberdeen, UK; 64 Department of Gynecology and Obstetrics, University of Munich, Munich, Germany; 65 Center for Familial Breast and Ovarian Cancer, Faculty of Medicine and University Hospital Cologne, University of Cologne, Cologne, Germany; 66 Center for Integrated Oncology (CIO), Faculty of Medicine and University Hospital Cologne, University of Cologne, Cologne, Germany; 67 Molecular Genetics of Breast Cancer, German Cancer Research Center (DKFZ), Heidelberg, Germany; 68 Department of Clinical Genetics, Rigshospitalet, Copenhagen University Hospital, Copenhagen, Denmark; 69 Southwest Thames Regional Genetics Service, St George’s Hospital, London, UK; 70 Institute of Human Genetics, University Hospital Leipzig, Leipzig, Germany; 71 Institute of Human Genetics, University of Münster, Münster, Germany; 72 The Hereditary Breast and Ovarian Cancer Research Group Netherlands (HEBON), Coordinating Center: The Netherlands Cancer Institute, Amsterdam, The Netherlands; 73 Clinical Genetics, Guy’s and St Thomas’ NHS Foundation Trust, London, UK; 74 Parkville Familial Cancer Centre, Peter MacCallum Cancer Center, Melbourne, Victoria, Australia; 75 Faculty of Medicine, Institute of Biomedical Sciences, Department of Human and Medical Genetics, Vilnius University, Vilnius, Lithuania; 76 State Research Institute Centre for Innovative Medicine, Vilnius, Lithuania; 77 Department of Clinical Genetics, Aarhus University Hospital, Aarhus N, Denmark; 78 Department of Oncology, Landspitali University Hospital, Reykjavik, Iceland; 79 Department of Epidemiology & Population Health, Stanford University School of Medicine, Stanford, CA, USA; 80 Department of Medicine, Division of Oncology, Stanford Cancer Institute, Stanford University School of Medicine, Stanford, CA, USA; 81 Department of Urology, Medical University of Vienna, Vienna, Austria; 82 Department of Clinical Genetics, Aalborg University Hospital, Aalborg, Denmark; 83 Clinical Cancer Research Center, Aalborg University Hospital, Aalborg, Denmark; 84 Genetic Epidemiology of Cancer Team, Inserm U900, Paris, France; 85 Magee-Womens Hospital, University of Pittsburgh School of Medicine, Pittsburgh, PA, USA; 86 Department of Surgery, National Institute of Oncology, Budapest, Hungary; 87 Immunology and Molecular Oncology Unit, Veneto Institute of Oncology IOV—IRCCS, Padua, Italy; 88 Department of Medicine, Abramson Cancer Center, Perelman School of Medicine at the University of Pennsylvania, Philadelphia, PA, USA; 89 Department of Cancer Epidemiology, Moffitt Cancer Center, Tampa, FL, USA; 90 Northern Ireland Regional Genetics Centre, Belfast City Hospital, Belfast, UK; 91 Department of Obstetrics and Gynecology, Helsinki University Hospital, University of Helsinki, Helsinki, Finland; 92 All Wales Medical Genetics Services, University Hospital of Wales, Cardiff, UK; 93 Precision Medicine, School of Clinical Sciences at Monash Health, Monash University, Clayton, Victoria, Australia; 94 Department of Clinical Pathology, The University of Melbourne, Melbourne, Victoria, Australia; 95 Department of Gynecology and Obstetrics, University Hospital Düsseldorf, Heinrich-Heine University Düsseldorf, Düsseldorf, Germany; 96 Center for Clinical Cancer Genetics, The University of Chicago, Chicago, IL, USA; 97 Cancer Risk Factors and Life-Style Epidemiology Unit, Institute for Cancer Research, Prevention and Clinical Network (ISPRO), Florence, Italy; 98 Department of Genetics and Computational Biology, QIMR Berghofer Medical Research Institute, Brisbane, Queensland, Australia; 99 Molecular Diagnostics, Aalborg University Hospital, Aalborg, Denmark; 100 Department of Clinical Medicine, Aalborg University, Aalborg, Denmark; 101 Genome Diagnostics Program, IFOM—the FIRC Institute of Molecular Oncology, Milan, Italy; 102 Department of Clinical Genetics, Vejle Hospital, Vejle, Denmark; 103 Department of Genetics, Portuguese Oncology Institute, Porto, Portugal; 104 South East of Scotland Regional Genetics Service, Western General Hospital, Edinburgh, UK; 105 Translational Research Laboratory, IDIBELL (Bellvitge Biomedical Research Institute), Catalan Institute of Oncology, CIBERONC, Barcelona, Spain; 106 Unit of Molecular Bases of Genetic Risk and Genetic Testing, Department of Research, Fondazione IRCCS Istituto Nazionale dei Tumori (INT), Milan, Italy; 107 Division of Gynaecology and Obstetrics, Klinikum rechts der Isar der Technischen Universität München, Munich, Germany; 108 Clinical Genetics, Karolinska Institutet, Stockholm, Sweden; 109 Clinical Genetics Service, Department of Medicine, Memorial Sloan Kettering Cancer Center, New York, NY, USA; 110 Institute for Clinical Genetics, Faculty of Medicine Carl Gustav Carus, Technische Universität Dresden, Dresden, Germany; 111 Servicio de Análisis Clínicos y Bioquímica Clínica, Complejo Hospitalario Universitario Insular Materno-Infantil de Gran Canaria , Las Palmas de Gran Canaría, Spain; 112 West Midlands Regional Genetics Service, Birmingham Women’s Hospital Healthcare NHS Trust, Birmingham, UK; 113 Princess Anne Hospital, Southampton, UK; 114 Department of OB/GYN and Comprehensive Cancer Center, Medical University of Vienna, Vienna, Austria; 115 Université Paris Descartes, Paris, France; 116 Institute of Human Genetics, University Hospital Heidelberg, Heidelberg, Germany; 117 Dept of OB/GYN, Medical University of Vienna, Vienna, Austria; 118 Biomedical Sciences Institute (ICBAS), University of Porto, Porto, Portugal; 119 Department of Medicine, Magee-Womens Hospital, University of Pittsburgh School of Medicine, Pittsburgh, PA, USA; 120 Program in Cancer Genetics, Departments of Human Genetics and Oncology, McGill University, Montréal, QC, Canada; 121 Department of Medical Genetics, University of Cambridge, Cambridge, UK; 122 Department of Cancer Biology and Genetics, The Ohio State University, Columbus, OH, USA; 123 IRCCS Istituto Tumori Giovanni Paolo II, Bari, Italy; 124 Department of Medicine, University of Melbourne, Melbourne, Victoria, Australia; 125 Department of Clinical Genetics, Leiden University Medical Center, Leiden, The Netherlands; 126 Clinical Genetics Research Lab, Department of Cancer Biology and Genetics, Memorial Sloan Kettering Cancer Center, New York, NY, USA; 127 Oxford Regional Genetics Service, Churchill Hospital, Oxford, UK; 128 Department of Gynaecology and Obstetrics, University Hospital Ulm, Ulm, Germany; 129 Liverpool Centre for Genomic Medicine, Liverpool Women’s NHS Foundation Trust, Liverpool, UK; 130 Department of Laboratory Medicine and Pathology, Mayo Clinic, Rochester, MN, USA; 131 Center for Molecular Medicine Cologne (CMMC), Faculty of Medicine and University Hospital Cologne, University of Cologne, Cologne, Germany; 132 Genomics Center, Centre Hospitalier Universitaire de Québec—Université Laval Research Center, Québec City, QC, Canada; 133 Centre for Cancer Genetic Epidemiology, Department of Oncology, University of Cambridge, Cambridge, UK

## Abstract

**Background:**

Recent population-based female breast cancer and prostate cancer polygenic risk scores (PRS) have been developed. We assessed the associations of these PRS with breast and prostate cancer risks for male *BRCA1 and BRCA2* pathogenic variant carriers.

**Methods:**

483 *BRCA1* and 1318 *BRCA2* European ancestry male carriers were available from the Consortium of Investigators of Modifiers of *BRCA1/2* (CIMBA). A 147-single nucleotide polymorphism (SNP) prostate cancer PRS (PRS_PC_) and a 313-SNP breast cancer PRS were evaluated. There were 3 versions of the breast cancer PRS, optimized to predict overall (PRS_BC_), estrogen receptor (ER)–negative (PRS_ER-_), or ER-positive (PRS_ER+_) breast cancer risk.

**Results:**

PRS_ER+_ yielded the strongest association with breast cancer risk. The odds ratios (ORs) per PRS_ER+_ standard deviation estimates were 1.40 (95% confidence interval [CI] =1.07 to 1.83) for *BRCA1* and 1.33 (95% CI = 1.16 to 1.52) for *BRCA2* carriers. PRS_PC_ was associated with prostate cancer risk for *BRCA1* (OR = 1.73, 95% CI = 1.28 to 2.33) and *BRCA2* (OR = 1.60, 95% CI = 1.34 to 1.91) carriers. The estimated breast cancer odds ratios were larger after adjusting for female relative breast cancer family history. By age 85 years, for *BRCA2* carriers, the breast cancer risk varied from 7.7% to 18.4% and prostate cancer risk from 34.1% to 87.6% between the 5th and 95th percentiles of the PRS distributions.

**Conclusions:**

Population-based prostate and female breast cancer PRS are associated with a wide range of absolute breast and prostate cancer risks for male *BRCA1* and *BRCA2* carriers. These findings warrant further investigation aimed at providing personalized cancer risks for male carriers and informing clinical management.

*BRCA1 and BRCA2* pathogenic variants are associated with increased male breast cancer and prostate cancer risks (1-4). A recent prospective study estimated the lifetime risk of developing prostate cancer to be 29% for *BRCA1* and 60% for *BRCA2* carriers (5). The risks of developing male breast cancer compared with the general population have been estimated to be 15- to 18-fold higher for *BRCA1* and 80-fold higher for *BRCA2* carriers (6,7). Up to 1 in 10 male *BRCA2* carriers develops breast cancer (8–12) and displays potentially more aggressive disease relative to sporadic cases (8,12,13).

Polygenic risk scores (PRS) that combine the effects of multiple disease-associated single nucleotide polymorphisms (SNPs) provide marked cancer risk stratification in the general population (14,15) and *BRCA1* and *BRCA2* carriers (16–18). Our previous findings suggested the joint effects of PRS and *BRCA1* and *BRCA2* pathogenic variants may identify men at clinically meaningful breast and prostate cancer risk levels (17). Recent studies have identified additional breast and prostate cancer susceptibility variants (15,19,20) and have refined PRS for these cancers (15,21).

The Breast Cancer Association Consortium recently developed and validated a 313-SNP PRS in European ancestry women, which was further optimized to predict estrogen receptor (ER)–specific disease (21). The estimated per standard deviation odds ratio (OR) for the most predictive (ER-positive) PRS was 1.68 (95% confidence interval [CI] = 1.63 to 1.73) (21). A recent evaluation of this PRS in unselected male breast cancer cases showed similar associations with breast cancer risk in men (22). The most recent prostate cancer PRS was developed using 147-SNPs associated with prostate cancer risk in European-ancestry men from the general population (15). The estimated per standard deviation odds ratio for the prostate cancer PRS was 1.86 (95% CI = 1.83 to 1.89) (15).

Male *BRCA1* and *BRCA2* carriers are likely to benefit from more personalized breast and prostate cancer risk estimates (23). Investigating the extent to which these PRS modify cancer risks may lead to more precise and gender-specific cancer risk assessment and could assist in optimizing cancer screening.

Here, we assessed the associations of the newly developed 313-SNP breast cancer PRS and 147-SNP prostate cancer PRS derived using population-based data, with breast and prostate cancer risks, respectively, for male *BRCA1* and *BRCA2* carriers. We investigated whether cancer family history influences the associations and if breast cancer associations differed by ER status or tumor grade. Furthermore, we assessed whether associations vary by age or *BRCA1* and *BRCA2* pathogenic variant characteristics (location; functional effect). We used the results to estimate age-specific absolute risks of developing breast and prostate cancers for male carriers by PRS distribution percentiles.

## Methods

Statistical analyses were performed using R-3.6.3 (R Foundation for Statistical Computing, Vienna, Austria) (commands can be found in the Supplementary Methods, available online).

### Study Participants and Genotyping

Male *BRCA1 and BRCA2* pathogenic variant carriers were recruited through 40 studies from 19 countries participating in the Consortium of Investigators of Modifiers of *BRCA1 and BRCA2* (CIMBA) (24). The majority of male carriers were ascertained through families attending cancer genetic clinics (96.9%; Supplementary Tables 1 and 2, available online). In this setting, individuals are referred to clinical genetics because of strong family or personal cancer history. The first individual in a family, screened for mutations, tends to be an affected individual diagnosed at a young age, most often a female relative with a young age at breast cancer diagnosis (24). When a pathogenic variant is identified, then other family members are tested for the same variant. All participants were aged 18 years or older and provided written informed consent. All studies were approved by local ethical review committees. A total of 1989 male *BRCA1* and *BRCA2* carriers of European ancestry were included in the present study, by selecting all available men with a breast or prostate cancer diagnosis and matched controls. Details of matching, genotyping, and quality control processes have been described previously (17) and in Supplementary Table 2 (available online).

Data collected included breast or prostate cancer diagnoses; age at diagnosis or interview; prostate cancer Gleason score; breast cancer ER status and grade; and family history of prostate, male breast, and female breast cancers among first- and second-degree relatives. *BRCA1* and *BRCA2* pathogenic variants (detailed pathogenicity description: http://cimba.ccge.medschl.cam.ac.uk/files/CIMBA_Mutation_Classification_guidelines_May16.pdf) were categorized according to their known or predicted effect on protein function: class I included loss-of-function variants expected to yield unstable or no protein; class II included variants likely to produce stable mutant proteins (25). Pathology data were obtained from pathology reviews; medical, pathology or tumor registry records; or immunohistochemical staining of tissue microarrays (26).

### Polygenic Risk Scores

PRS were constructed as the weighted sums of alleles (Supplementary Methods, available online) for 313-SNPs for breast cancer (21) and 147-SNPs for prostate cancer (15) (Supplementary Tables 3 and 4, available online). Three breast cancer PRS were evaluated, optimized to predict overall (PRS_BC_), ER-negative (PRS_ER__-_), and ER-positive (PRS_ER+_) breast cancer (21). These PRS were scaled to the female population-based control PRS standard deviation (21). The prostate cancer PRS (PRS_PC_) was scaled to the standard deviation calculated from population-based controls (15).

### Associations Between PRS and Cancer Risks

PRS associations with breast and prostate cancer risks were assessed simultaneously using multinomial logistic regression to estimate per standard deviation odds ratios. Men without breast or prostate cancer diagnoses were considered controls. Breast and prostate cancer cases were defined by considering the first occurring cancer. Instances in which breast and prostate cancers were diagnosed simultaneously were considered as breast cancer cases. Statistical models were adjusted for 3 ancestry informative principal components (proxy adjustment for study and/or country , as a direct adjustment would result in too few controls and cases within each study and/or country; Supplementary Table 1, available online) and age. Models using the combined sample of carriers were adjusted for *BRCA1* and *BRCA2* status. To account for relatedness, we estimated robust variances by clustering on family membership (27,28). The primary analyses assumed a continuous PRS. Categorical PRS associations were evaluated using the quartiles of the PRS distributions in the combined *BRCA1* and *BRCA2* carrier controls.

Because the distribution of tumor ER status in male carriers may differ from the distributions in the general population (26), we assessed the associations between all 3 versions of the breast cancer PRS with overall breast cancer risk and ER-specific disease. Associations with ER-positive and ER-negative breast cancer were assessed simultaneously by considering ER negative, ER positive, or unknown as distinct multinomial outcomes. We also assessed the associations with breast cancer grade-specific risk by considering grade 1, grade 2, grade 3, or unknown grade as separate multinomial outcomes. A case-only logistic regression also was undertaken that considered grades 1 and 2 as controls and grade 3 as cases.

To assess the PRS_PC_ association with disease aggressiveness, we partitioned prostate cancers into those with Gleason scores less than 7, 7 or greater, or unknown, and these were used as distinct multinomial outcomes. A case-only logistic regression assessed differences in the associations with Gleason scores less than 7 (controls) and Gleason scores of 7 or greater (cases).

Discriminatory ability of each PRS was assessed by calculating the area under the receiver operator characteristic curve (AUC). Under the sampling design, the majority of male carriers were identified through clinical genetics. Therefore, the majority of both affected and unaffected carriers are expected to have family history of cancer. To determine whether this introduces any biases in the PRS associations, we fitted models that were adjusted for family history in first- and second-degree relatives.

To determine whether PRS associations varied by age (continuous), pathogenic variant location, or pathogenic variant effects on protein function (class I or class II variants), we estimated interaction terms between these factors with the PRS, and statistical significance was assessed using likelihood ratio tests (LRT). Pathogenic variants were categorized based on previously reported nucleotide position differences in breast and ovarian, or prostate cancer risks (29–31).

We undertook a sensitivity analysis to test for PRS heterogeneity across study countries (Supplementary Methods, available online).

All statistical tests were 2-sided, and a *P* value of less than .05 was considered statistically significant.

### Predicted Age-Specific Absolute and 10-Year Cancer Risks by PRS

We predicted absolute risks up to age 85 years and 10-year risks of developing breast and prostate cancers by PRS distribution percentiles, assuming the estimated PRS odds ratio follows a log-linear model across the entire PRS range (Supplementary Methods, available online) (32).

## Results

### Study Participants and Genotyping

After quality control, the analyses included 483 *BRCA1* (33 breast and 70 prostate cancer cases) and 1318 *BRCA2* (244 breast and 141 prostate cancer cases) carriers of European ancestry (Supplementary Tables 1 and 2, available online).

All SNPs from both PRS were well imputed (*r*^2^≥ 0.76; Supplementary Tables 3 and 4, Supplementary Figures 1 and 2, available online). Average PRS were larger for cases compared with controls (Supplementary Table 2, available online).

### Associations With Breast Cancer Risk

The associations between the breast cancer PRS and male breast cancer risk for carriers are shown in Table 1 and Supplementary Tables 5 and 6 (available online). The PRS_ER+_ yielded the strongest associations with overall breast cancer risk for *BRCA1* (OR = 1.40, 95% CI = 1.07 to 1.83) and *BRCA2* (OR = 1.33, 95% CI = 1.16 to 1.52) carriers. The PRS_BC_ resulted in nearly identical associations as the PRS_ER+_. There was no statistically significant evidence that the PRS_ER+_ associations differed by country (*P*_heterogeneity_ ≥ .48; Supplementary Figure 3, available online). In the joint analysis of *BRCA1* and *BRCA2* carriers, men in the uppermost PRS_ER+_ quartile had approximately twofold increased breast cancer risk (OR = 2.10, 95% CI = 1.43 to 3.08) compared with men in the lowest quartile (Supplementary Table 6, available online).

**Table 1.** Breast cancer PRS associations with breast cancer risk for *BRCA1 and BRCA2* carriers

PRS investigated and outcome	*BRCA1* carriers				*BRCA2* carriers			
	No. of controls	No. of cases	OR (95% CI)	*P* ^a^	No. of controls	No. of cases	OR (95% CI)	*P* ^a^
PRS_BC_								
PRS_BC_ association with breast cancer risk								
Continuous^b^	380	33	1.40 (1.06 to 1.85)	.02	933	244	1.32 (1.15 to 1.52)	<.001
Continuous: adjusted for male relative breast cancer FH^c^	380	33	1.39 (1.05 to 1.84)	.02	933	244	1.33 (1.15 to 1.52)	<.001
Continuous: adjusted for female relative breast cancer FH^c^	380	33	1.44 (1.07 to 1.95)	.02	933	244	1.36 (1.18 to 1.57)	<.001
PRS_BC_ association with grade-specific breast cancer risk^d^								
Controls	380	—	1.00 (referent)		933	—	1.00 (referent)	
Grade 1	—	1	1.03 (0.63 to 1.67)^g^	.92	—	11	1.33 (0.74 to 2.36)	.34
Grade 2	—	6			—	68	1.29 (1.04 to 1.60)	.02
Grade 3	—	12	1.56 (1.03 to 2.37)	.04	—	98	1.23 (1.00 to 1.50)	.05
Grade unknown	—	14	1.47 (0.93 to 2.32)	.10	—	67	1.51 (1.18 to 1.93)	.001
Case-only: grade 1 + 2 vs grade 3^e^	7	12	6.30 (0.88 to 44.87)	.07	79	98	0.95 (0.71 to 1.27)	.73
PRS_ER-_								
PRS_ER-_ association with breast cancer risk								
Continuous^b^	380	33	1.12 (0.79 to 1.59)	.52	933	244	1.23 (1.07 to 1.41)	.004
Continuous: adjusted for male relative breast cancer FH^c^	380	33	1.12 (0.79 to 1.59)	.53	933	244	1.23 (1.07 to 1.42)	.004
Continuous: adjusted for female relative breast cancer FH^c^	380	33	1.14 (0.80 to 1.63)	.48	933	244	1.25 (1.09 to 1.45)	.002
PRS_ER-_ association with ER-specific breast cancer risk^f^								
Controls	380	—	1.00 (referent)		933	—	1.00 (referent)	
ER negative	—	2	0.38 (0.06 to 2.29)	.29	—	7	0.51 (0.27 to 0.98)	.04
ER positive	—	21	1.47 (0.97 to 2.24)	.07	—	178	1.26 (1.08 to 1.47)	.004
ER status unknown	—	10	0.78 (0.46 to 1.30)	.34	—	59	1.24 (0.94 to 1.64)	.13
PRS_ER+_								
PRS_ER+_ association with breast cancer risk								
Continuous^b^	380	33	1.40 (1.07 to 1.83)	.01	933	244	1.33 (1.16 to 1.52)	<.001
Continuous: adjusted for male relative breast cancer FH^c^	380	33	1.39 (1.06 to 1.82)	.02	933	244	1.33 (1.16 to 1.53)	<.001
Continuous: adjusted for female relative breast cancer FH^c^	380	33	1.46 (1.09 to 1.94)	.01	933	244	1.36 (1.18 to 1.57)	<.001
PRS_ER+_ association with ER-specific breast cancer risk^f^								
Controls	380	—	1.00 (referent)		933	—	1.00 (referent)	
ER negative	—	2	0.35 (0.03 to 3.59)	.37	—	7	0.68 (0.38 to 1.22)	.20
ER positive	—	21	1.79 (1.30 to 2.48)	<.001	—	178	1.30 (1.11 to 1.52)	<.001
ER status unknown	—	10	1.00 (0.68 to 1.47)	1.00	—	59	1.52 (1.18 to 1.94)	.001
PRS_ER+_ association with grade-specific breast cancer risk^d^								
Controls	380	—	1.00 (referent)		933	—	1.00 (referent)	
Grade 1	—	1	1.03 (0.65 to 1.65)^g^	.89	—	11	1.31 (0.76 to 2.27)	.34
Grade 2	—	6			—	68	1.29 (1.05 to 1.59)	.02
Grade 3	—	12	1.51 (1.04 to 2.19)	.03	—	98	1.23 (1.01 to 1.51)	.04
Grade unknown	—	14	1.51 (0.96 to 2.38)	.07	—	67	1.51 (1.19 to 1.92)	<.001
Case-only: grade 1 + 2 vs grade 3^e^	7	12	5.41 (0.79 to 37.20)	.09	79	98	0.95 (0.71 to 1.28)	.75

^a^ *P* value was calculated using a 2-sided Wald test. CI = confidence interval; ER = estrogen receptor; FH = family history; OR = odds ratio per PRS standard deviation, estimated from a multinomial logistic regression (unless otherwise stated); PRS = polygenic risk scores PRS_BC_ = overall breast cancer PRS; PRS_ER-_ = ER-negative breast cancer PRS; PRS_ER+_ = ER-positive breast cancer PRS.

^b^ The continuous test shows the per PRS standard deviation associations, estimated from a multinomial logistic regression model assuming a continuous PRS.

^c^ Association estimates adjusted for family history of (male and female) breast cancer in first- and second-degree relatives. FH was coded as no family history, 1 or more relatives diagnosed with breast cancer, unknown FH or missing FH. Supplementary Table 8 (available online; male breast cancer FH adjusted) and Supplementary Table 9 (available online; female breast cancer FH adjusted) describe the breast cancer FH adjusted analyses in greater detail.

^d^ The breast cancer grade specific odds ratios were estimated by partitioning breast cancer status into multinomial outcomes for grade 1, grade 2, grade 3, or grade unknown.

^e^ The case-only breast cancer grade analysis was a logistic regression considering grade 1 and grade 2 breast cancers combined as controls and grade 3 breast cancers as cases.

^f^ The ER-specific breast cancer odds ratios were estimated by partitioning breast cancer status into distinct multinomial outcomes for ER negative, ER positive, or ER status unknown.

^g^ Grade 1 and grade 2 combined for *BRCA1* carriers (to ensure adequate sample size to estimate associations).

Most breast cancers among the male carriers were ER positive (95.7%). The odds ratio for the association between the PRS_ER+_ and ER-positive breast cancer risk for *BRCA1* carriers (OR = 1.79, 95% CI = 1.30 to 2.48; Table 1) was somewhat higher compared with the odds ratio for overall breast cancer. The number of ER-negative cancers was too small to assess associations with ER-negative disease. There was no statistically significant evidence for differences in the associations of any of the PRS by grade (Table 1; Supplementary Table 6, available online).

The ability of PRS_ER+_ to discriminate between controls and breast cancer cases was estimated as an AUC of 0.60 (95% CI = 0.51 to 0.69) for *BRCA1* and 0.59 (95% CI = 0.55 to 0.63) for *BRCA2* carriers.

### Associations With Prostate Cancer Risk

The estimated associations between the PRS_PC_ and prostate cancer risk for male carriers are reported in Table 2 and Supplementary Tables 5 and 7 (available online). The odds ratios per PRS_PC_ standard deviation were estimated to be 1.73 (95% CI = 1.28 to 2.33) for *BRCA1* and 1.60 (95% CI = 1.34 to 1.91) for *BRCA2* carriers. There was no statistically significant evidence that the PRS_PC_ associations differed by country (*P*_heterogeneity_ ≥ .14; Supplementary Figure 4, available online). In the joint analysis of *BRCA1* and *BRCA2* carriers, men in the top PRS_PC_ quartile had a prostate cancer odds ratio of 3.35 (95% CI = 2.06 to 5.42) compared with men in the lowest quartile (Supplementary Table 7, available online).

**Table 2.** Prostate cancer PRS associations with prostate cancer risk for *BRCA1 and BRCA2* carriers

PRS investigated and outcome	*BRCA1* carriers				*BRCA2* carriers			
	No. of controls	No. of cases	OR (95% CI)	*P* ^a^	No. of controls	No. of cases	OR (95% CI)	*P* ^a^
Continuous^b^	380	70	1.73 (1.28 to 2.33)	<.001	933	141	1.60 (1.34 to 1.91)	<.001
Continuous: adjusted for FH^c^	380	70	1.74 (1.29 to 2.35)	<.001	933	141	1.59 (1.32 to 1.90)	<.001
PRS_PC_ association with Gleason score (GS)–specific prostate cancer risk^d^								
Controls	380	—	1.00 (referent)		933	—	1.00 (referent)	
GS < 7	—	26	1.11 (0.70 to 1.77)	.66	—	27	1.83 (1.29 to 2.58)	<.001
GS ≥ 7	—	21	2.09 (1.27 to 3.46)	.004	—	82	1.68 (1.32 to 2.13)	<.001
GS unknown	—	23	2.38 (1.49 to 3.80)	<.001	—	32	1.26 (0.95 to 1.68)	.11
Case-only analysis: GS ≥ 7 vs GS < 7^e^	26	21	1.87 (1.01 to 3.44)	.05	27	82	0.93 (0.63 to 1.37)	.72

^a^ *P* value was calculated using a 2-sided Wald test. CI = confidence interval; GS = Gleason score; FH = family history; OR = odds ratio per PRS standard deviation, estimated from a multinomial logistic regression (unless otherwise stated); PRS = polygenic risk scores; PRS_PC_ = prostate cancer PRS.

^b^ The continuous test shows the per PRS standard deviation associations, estimated from a multinomial logistic regression model assuming a continuous PRS.

^c^ Association estimates adjusted for family history of prostate cancer in first- and second-degree relatives. FH was coded as no family history, 1 or more diagnosed relatives, unknown FH, or missing FH. Supplementary Table 10 (available online) describes the prostate cancer FH adjusted analyses in greater detail.

^d^ The Gleason score prostate cancer odds ratios were estimated by partitioning prostate cancer status into distinct multinomial outcomes for GS < 7, GS ≥ 7, or GS unknown.

^e^ The case-only prostate cancer analysis was a logistic regression considering GS < 7 prostate cancers as “controls” and GS ≥ 7 prostate cancers as “cases”.

There was a suggestion of higher risk for aggressive disease for *BRCA1* carriers (Gleason score ≥7: OR = 2.09, 95% CI = 1.27 to 3.46; Gleason score <7: OR = 1.11, 95% CI = 0.70 to 1.77), also supported by the case-only analysis (OR = 1.87, 95% CI = 1.01 to 3.44; *P* = .05; Table 2). There were no differences in the PRS_PC_ associations with high- or low-Gleason score among *BRCA2* carriers (Table 2).

The PRS_PC_ discriminatory ability was estimated as an AUC of 0.62 (95% CI = 0.54 to 0.69) for *BRCA1* and 0.62 (95% CI = 0.57 to 0.67) for *BRCA2* carriers.

### Adjusting for Cancer Family History

Adjusting for family history of male breast cancer did not influence the PRS_ER+_ associations with breast cancer risk (Table 1;Supplementary Table 8, available online). However, the odds ratio estimates were somewhat larger when adjusting for female breast cancer family history (Table 1;Supplementary Table 9, available online).

The associations of PRS_PC_ with prostate cancer risk remained similar after adjusting for prostate cancer family history (Table 2;Supplementary Table 10, available online).

### PRS Interactions With Age and Gene Pathogenic Variants Characteristics

There was little evidence for odds ratio estimate variability with age, for both the breast and prostate cancer PRS (P_LRT_ ≥ .43; Table 3).

**Table 3.** PRS interactions with age and *BRCA1 and BRCA2* pathogenic variant characteristics for *BRCA1 and BRCA2* carriers with breast cancer risk and prostate cancer risk.

Model and category	Breast cancer (PRS_ER+_)^a^				Prostate cancer (PRS_PC_)			
	*BRCA1* carriers		*BRCA2* carriers		*BRCA1* carriers		*BRCA2* carriers	
	OR (95% CI)	*P* ^b^	OR (95% CI)	*P* ^b^	OR (95% CI)	*P* ^b^	OR (95% CI)	*P* ^b^
PRS x age interaction^c^								
PRS	1.88 (0.68 to 5.18)	.22	1.34 (0.71 to 2.53)	.37	0.64 (0.20 to 2.04)	.45	2.03 (0.91 to 4.52)	.08
PRS x age	1.00 (0.98 to 1.01)	.56	1.00 (0.99 to 1.01)	.98	1.02 (1.00 to 1.03)	.09	1.00 (0.98 to 1.01)	.55
P_LRT_^d^		.90		.86		.43		.79
Gene pathogenic variant class^e^								
Class I	1.38 (1.03 to 1.84)	.03	1.31 (1.13 to 1.52)	<.001	1.57 (1.13 to 2.19)	.008	1.57 (1.31 to 1.89)	<.001
Class II	1.71 (0.72 to 4.07)	.23	1.39 (0.67 to 2.86)	.38	3.00 (1.36 to 6.60)	.006	2.04 (0.63 to 6.55)	.23
P_LRT_^d^		.76		.69		.26		.97
*BRCA1* pathogenic variant location (OCCR)								
5’ to c.2281	1.50 (1.00 to 2.26)	.05	NA		NA		NA	
c.2282 to c.4071	1.17 (0.79 to 1.72)	.44	NA		NA		NA	
c.4072 to 3’	1.61 (0.87 to 2.98)	.13	NA		NA		NA	
P_LRT_^d^		.85						
*BRCA2* pathogenic variant location (OCCR)								
5’ to c.2830	NA		1.43 (1.09 to 1.88)	.009	NA		NA	
c.2831 to c.6401	NA		1.24 (0.99 to 1.55)	.06	NA		NA	
c.6402 to 3’	NA		1.33 (1.04 to 1.70)	.02	NA		NA	
P_LRT_^d^				.61				
*BRCA2* pathogenic variant location (PCCR)								
5’ to c.755	NA		NA		NA		1.67 (1.06 to 2.62)	.03
c.756 to c.1000	NA		NA		NA		1.77 (1.07 to 2.95)	.03
c.1001 to c.7913	NA		NA		NA		1.49 (1.18 to 1.89)	<.001
c.7914 to 3’	NA		NA		NA		1.76 (1.24 to 2.50)	.002
P_LRT_^d^								.52

^a^ The associations with breast cancer risk are reported for the ER-positive breast cancer PRS (PRS_ER+_). CI = confidence interval; OCCR = ovarian cancer cluster region; OR = odds ratio per PRS standard deviation, estimated from a multinomial logistic regression; PCCR = prostate cancer cluster region; PRS = polygenic risk score; NA = not applicable.

^b^ *P* value was calculated using a 2-sided Wald test, unless otherwise indicated.

^c^ The PRS term is applicable at age 0 years and the PRS x age interaction term is a per-year effect. Age in years.

^d^ *P* values were calculated using a 2-sided likelihood ratio test. The likelihood ratio test compared the model that estimated the interaction term with a nested model that omitted the interaction term.

^e^ Class I pathogenic variant = loss-of-function pathogenic variants expected to result in unstable or no protein; class II pathogenic variant = pathogenic variants likely to yield stable mutant proteins.

The PRS_ER+_ and PRS_PC_ odds ratios with breast or prostate cancer risks appeared to be larger for class II variant (pathogenic variants likely to yield stable mutant proteins) carriers compared with class I *BRCA1* and *BRCA2* variant carriers (Table 3). However, these differences were not statistically significant (P_LRT_ ≥ .26).

There was no statistically significant evidence that the PRS_ER+_ (P_LRT_ ≥ .61) or PRS_PC_ (P_LRT_ = .52) associations differed by the pathogenic variant location in the gene (Table 3).

### Absolute Risks of Developing Breast and Prostate Cancer

**Figure 1.**
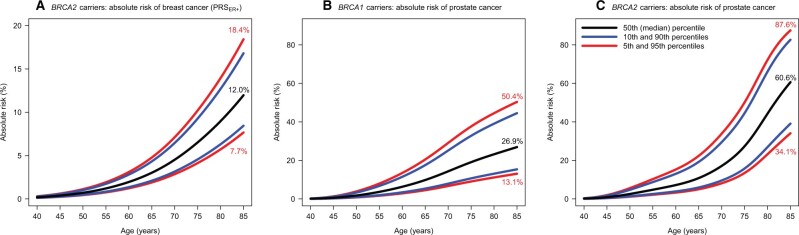
The predicted absolute risks of developing breast cancer and prostate cancer by PRS percentile. Risks were calculated assuming the per standard deviation ratio estimates in the combined sample of *BRCA1 and BRCA2* carriers (Supplementary Tables 6 and 7). **(A)** The absolute risks of developing breast cancer for *BRCA2* carriers by PRS_ER+_ percentiles. **(B)** The absolute risks of developing prostate cancer for *BRCA1* carriers by PRS_PC_ percentiles. **(C)** The absolute risks of developing prostate cancer for *BRCA2* carriers by PRS_PC_ percentiles. PRS = polygenic risk scores; PRS_ER+_ = ER-positive breast cancer PRS.

**Figure 2.**
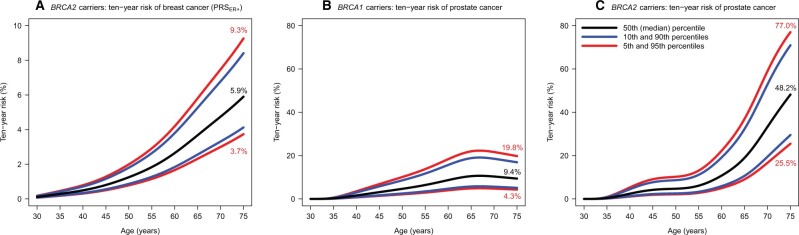
The predicted 10-year risks of developing breast cancer and prostate cancer by PRS percentile. Ten-year risks were calculated from the absolute risks of developing breast cancer or prostate cancer (Figure 1). **(A)** The 10-year risks of developing breast cancer for *BRCA2* carriers by PRS_ER+_ percentiles. **(B)** The 10-year risks of developing prostate cancer for *BRCA1* pathogenic variant carriers by PRS_PC_ percentiles. **(C)** The 10-year risks of developing prostate cancer for *BRCA2* pathogenic variant carriers by PRS_PC_ percentiles. PRS = polygenic risk scores PRS_ER+_ = ER-positive breast cancer PRS.

The absolute risks of developing breast cancer by age 85 years for *BRCA2* carriers was predicted to be 7.7% at the 5th and 18.4% at the 95th PRS_ER+_ distribution percentiles (Figure 1). The 10-year risks of developing breast cancer at 50 years were 0.8% at the 5th and 2.0% at the 95th PRS_ER+_ distribution percentiles for *BRCA2* carriers (Figure 2). The corresponding risks at age 75 years were 3.7% and 9.3%, respectively.

The predicted absolute risks of developing prostate cancer by age 85 years were 13.1% at the 5th and 50.4% at the 95th PRS_PC_ distribution percentiles for *BRCA1* carriers (Figure 1). The corresponding risks for *BRCA2* carriers were 34.1% and 87.6%. *BRCA2* carriers had 10-year risks of 2.1% and 10.1% at the 5th and 95th PRS_PC_ percentiles at age 50 years, respectively. The corresponding risks at age 75 years were 25.5% and 77.0% (Figure 2).

## Discussion

We evaluated the associations of the most recently developed breast and prostate cancer PRS with site-specific cancer risks in the largest case-control study of male *BRCA1 and BRCA2* carriers available to date. Our findings showed that these PRS, developed using population-based data, are associated with breast and prostate cancer risks for male *BRCA1* and *BRCA2* carriers. Despite the modest estimated AUCs, our results demonstrate that because male carriers are already at elevated risks of developing breast and prostate cancers, these PRS can lead to large differences in the absolute cancer risks for carriers across PRS percentiles.

PRS_BC_ and PRS_ER+_ were associated with larger odds ratio estimates than PRS_ER-_ in predicting breast cancer risk, consistent with the fact that most breast cancers in men are ER positive, including those harboring *BRCA1* and *BRCA2* pathogenic variants (26). Similarly, when assessing associations with ER-positive breast cancer risk, PRS_BC_ and PRS_ER+_ showed the strongest associations for *BRCA1* and *BRCA2* carriers. There were no differences in PRS associations by breast cancer grade.

The 147-SNP PRS_PC_ (15) yielded larger per standard deviation odds ratio estimates than a previously evaluated 103-SNP prostate cancer PRS (17). There was some evidence that PRS_PC_ may be associated with a higher odds ratio for more aggressive disease (Gleason score ≥7) for *BRCA1* carriers. This pattern was not observed for *BRCA2* carriers, who tend to develop more aggressive disease (5). If this finding is replicated by larger studies, the PRS may prove to be useful in cancer prevention and surveillance by identifying *BRCA1* carriers at greater risk of developing aggressive prostate cancers.

PRS associations with breast or prostate cancer risk, adjusted for family history of male breast or prostate cancer, were similar to unadjusted estimates, suggesting that cancer family history in male relatives does not alter PRS associations. Adjusting for family history of female breast cancer resulted in somewhat larger odds ratio estimates for the breast cancer PRS compared with unadjusted estimates. This observation is consistent with male carriers being identified and recruited into our studies mostly based on their female relatives’ breast cancers.

There was little evidence supporting variability in PRS associations by age or pathogenic variant characteristics. However, larger sample sizes are required to reliably assess such differences, and the current analyses were likely underpowered.

Previous studies (18,33) suggest the magnitude of the breast cancer PRS associations is attenuated in female *BRCA1* and *BRCA2* carriers compared with associations seen in the general population (21). As seen for female carriers, the estimated breast cancer odds ratios for male carriers were attenuated compared with estimates for women in the general population (21). Similarly, the estimated prostate cancer odds ratio estimate for male carriers was attenuated compared with population-based data (15). Taken together, these observations suggest there is a deviation from the multiplicative model for the joint effects of *BRCA1* and *BRCA2* pathogenic variants and the PRS for male and female carriers. These observed attenuations for *BRCA1* and *BRCA2* carriers are unlikely to be an overestimation of the effects in the general population [“winner’s curse” (34)], as they have been validated in independent prospective cohorts (21). The lower odds ratios for the breast and prostate cancer PRS in male *BRCA1* and *BRCA2* carriers, compared with the general population, may reflect a general attenuation of the effect sizes of common variants on genetic risk in the presence of a pathogenic variant in a high-risk gene (35,36). This supposition may also explain the larger PRS odds ratios for *BRCA1* carriers, who are at lower risk compared with *BRCA2* carriers (37). However, given the current study design, we cannot rule out that the observed attenuations in effect size are related to ascertainment biases. Although adjusting for family history did not change the odds ratio estimates substantially, residual confounding may still remain. Large-scale population studies will be required to address this. If the attenuations in the PRS effect size are real, they would result in a smaller range of cancer risks for *BRCA1* and *BRCA2* carriers compared with using the PRS effect sizes estimated from general population data.

Although breast cancer risk stratification might not currently be feasible for men in the general population, male *BRCA1* and *BRCA2* carriers may represent a group likely to benefit from a more refined stratification of their individual breast and prostate cancer risks, to better inform their clinical management. At present, limited recommendations based on low-level evidence or expert opinion are available for male carriers. Current guidelines recommend clinical breast examinations beginning at ages 30-35 years and suggest mammographic screening on an individual basis, whereas clinical prostate cancer screening, particularly for *BRCA2* carriers, is recommended from ages 40 to 45 years (38–40).

The PRS percentile-specific absolute risks varied substantially over the PRS distribution, consistent with previous studies in male (17) and female (16,18) *BRCA1* and *BRCA2* carriers. At least twofold increased risk is often considered a clinically actionable level for breast and prostate cancers (41). Our findings may inform the development of age-specific clinical recommendations and provide guidance on when to start risk-adapted screening, based on their PRS percentile-specific 10-year risks. Overall, refined risk estimates may be useful to distinguish male carriers at higher risk, who may benefit from enhanced and/or earlier screening, and identify carriers at lower risk, who may opt for more limited or postponed surveillance. Identification of men at lower risk of prostate cancer by PRS stratification has been shown to be useful in reducing overdiagnosis in the general population, resulting in a reduction in the harms associated with prostate-specific antigen testing (42). Similar arguments may apply to male carriers in whom PRS prediction may further improve screening efficacy.

Strengths of this study include the fact that this is an independent validation of the most recently derived breast (21) and prostate (15) cancer PRS derived from population-based data. We benefited from the availability of Gleason scores and breast cancer ER status and grade; hence, we could assess subtype-specific associations. Finally, we assumed recent prospectively estimated prostate cancer incidence rates (5) to predict absolute prostate cancer risks, which may be more representative of risks for carriers currently seen in clinical genetics centers.

Study limitations include the limited sample size to assess PRS associations with cancer risks for subgroups of male carriers. However, these data remain the largest male *BRCA1* and *BRCA2* carrier case-control study with available genotype data. The breast (21) and prostate (15) cancer PRS do not include male breast cancer-specific risk-associated SNPs or SNPs that may specifically be associated with prostate cancer risk for carriers. If such SNPs exist, further improvement may be gained in risk prediction by including them in PRS. The absolute risk calculations assumed that the PRS odds ratio behaves log linearly over the PRS range. It was difficult to evaluate this assumption in the present analyses because of the limited sample size of male carriers. However, empirical evidence based on larger sample sizes of female carriers (18) or in the general population (15,21) suggests that this assumption is plausible. Additionally, the absolute breast and prostate cancer risk predictions by PRS will require validation in large prospective studies of male carriers with long-term follow-up, although such studies remain a challenge. Finally, the PRS that we investigated were derived using European ancestry data; hence, our estimated associations and predicted risks may not be applicable to non-European ancestry carriers.

PRS are now used in cancer risk–stratified screening trials and implementation studies in the general population (43–47). They are commercially available and are used in multifactorial cancer-risk prediction models for women (48,49). We found that PRS derived from population-based data are associated with breast and prostate cancer risks and lead to meaningful risk stratification for male carriers. These findings may potentially be used to provide more personalized cancer risk predictions and therefore assist clinical management decisions. Future implementation studies should determine if optimal strategies exist for incorporating these PRS into genetic counseling and risk assessment to clarify whether they can influence the clinical management decisions of male *BRCA1* or *BRCA2* carriers.

## Funding

The CIMBA data management and data analysis were supported by Cancer Research UK grants C12292/A20861 and PPRPGM-Nov20\100002. The research leading to these results has received funding from the Italian Association for Cancer Research (AIRC) under IG 2018 - ID. 21389 and the Italian League for the Fight Against Cancer (LILT) under IG 2019 projects, P.I. Ottini Laura and Italian Ministry of Education, Universities and Research-Dipartimenti di Eccellenza-L. 232/2016. CIMBA: GCT is a National Health and Medical Research Council (NHMRC) Research Fellow. iCOGS and OncoArray data: the European Community’s Seventh Framework Programme under grant agreement No. 223175 (HEALTH-F2-2009-223175) (COGS), Cancer Research UK (C1287/A10118, C1287/A 10710, C12292/A11174, C1281/A12014, C5047/A8384, C5047/A15007, C5047/A10692, C8197/A16565), the National Institutes of Health (NIH) (CA128978) and Post-Cancer GWAS initiative (1U19 CA148537, 1U19 CA148065 and 1U19 CA148112 - the GAME-ON initiative), the Department of Defence (W81XWH-10-1-0341), the Canadian Institutes of Health Research (CIHR) for the CIHR Team in Familial Risks of Breast Cancer (CRN-87521), and the Ministry of Economic Development, Innovation and Export Trade (PSR-SIIRI-701), Komen Foundation for the Cure, the Breast Cancer Research Foundation, and the Ovarian Cancer Research Fund. The Personalized Risk Stratification for Prevention and Early Detection of Breast Cancer (PERSPECTIVE) and PERSPECTIVE I&I projects were supported by the Government of Canada through Genome Canada and the Canadian Institutes of Health Research, the Ministry of Economy and Innovation through Genome Québec, and The Quebec Breast Cancer Foundation and the Ontario Research Fund. Breast Cancer Family Registry (BCFR): UM1 CA164920 from the National Cancer Institute (NCI). Baltic Familial Breast Ovarian Cancer Consortium (BFBOCC): Lithuania (BFBOCC-LT): Research Council of Lithuania grant SEN-18/2015. Beth Israel Deaconess Medical Center (BIDMC): Breast Cancer Research Foundation. BRCA-gene mutations and breast cancer in South African women (BMBSA): Cancer Association of South Africa (PI Elizabeth J. van Rensburg). Spanish National Cancer Centre (CNIO): Spanish Ministry of Health PI16/00440 supported by Fondo Europeo de Desarrollo Regional (FEDER) funds, the Spanish Ministry of Economy and Competitiveness (MINECO) SAF2014-57680-R and the Spanish Research Network on Rare diseases (CIBERER). City of Hope - Clinical Cancer Genomics Community Research Network (COH-CCGCRN): Research reported in this publication was supported by the NCI of the NIH under grant No. R25CA112486, and RC4CA153828 (PI: J. Weitzel) from the NCI and the Office of the Director, NIH. CONsorzio Studi ITaliani sui Tumori Ereditari Alla Mammella (CONSIT TEAM): Associazione Italiana Ricerca sul Cancro (AIRC; IG2014 No.15547) to P. Radice. Funds from Italian citizens who allocated the 5x1000 share of their tax payment in support of the Fondazione IRCCS Istituto Nazionale Tumori, according to Italian laws (INT-Institutional strategic projects ‘5x1000’) to S. Manoukian. Associazione CAOS Varese to M.G. Tibiletti. AIRC (IG2015 No.16732) to P. Peterlongo. National Centre for Scientific Research Demokritos (DEMOKRITOS): European Union (European Social Fund—ESF) and Greek national funds through the Operational Program “Education and Lifelong Learning” of the National Strategic Reference Framework (NSRF) - Research Funding Program of the General Secretariat for Research & Technology: SYN11_10_19 NBCA. Investing in knowledge society through the European Social Fund. German Cancer Research Center (DFKZ): German Cancer Research Center. Epidemiological Study of Familial Breast Cancer (EMBRACE): Cancer Research UK Grants C1287/A10118 and C1287/A11990. D. Gareth Evans and Fiona Lalloo are supported by an National Institute for Health Research (NIHR) grant to the Biomedical Research Centre, Manchester. The Investigators at The Institute of Cancer Research and The Royal Marsden National Health Service (NHS) Foundation Trust are supported by an NIHR grant to the Biomedical Research Centre at The Institute of Cancer Research and The Royal Marsden NHS Foundation Trust. Ros Eeles and Elizabeth Bancroft are supported by Cancer Research UK Grant C5047/A8385. Ros Eeles is also supported by NIHR support to the Biomedical Research Centre at The Institute of Cancer Research and The Royal Marsden NHS Foundation Trust. Fox Chase Cancer Center (FCCC): The University of Kansas Cancer Center (P30 CA168524) and the Kansas Bioscience Authority Eminent Scholar Program. AKG was in part funded by the NCI (R01 CA214545 and R01 CA140323), The Kansas Institute for Precision Medicine (P20 GM130423), and the Kansas Bioscience Authority Eminent Scholar Program. A.K.G. is the Chancellors Distinguished Chair in Biomedical Sciences Professor. Fundación Pública Galega de Medicina Xenómica (FPGMX): FISPI05/2275 and Mutua Madrileña Foundation (FMMA). German Familial Breast Group (GC-HBOC): German Cancer Aid (grant No. 110837, Rita K. Schmutzler) and the European Regional Development Fund and Free State of Saxony, Germany (LIFE—Leipzig Research Centre for Civilization Diseases, project No. 713-241202, No. 713-241202, No. 14505/2470, and No. 14575/2470). Genetic Modifiers of cancer risk in BRCA1/2 mutation carriers (GEMO): Ligue Nationale Contre le Cancer; the Association “Le cancer du sein, parlons-en!” Award, the Canadian Institutes of Health Research for the “CIHR Team in Familial Risks of Breast Cancer” program and the French National Institute of Cancer (INCa grants 2013-1-BCB-01-ICH-1 and SHS-E-SP 18-015). Georgetown University (GEORGETOWN): the Non-Therapeutic Subject Registry Shared Resource at Georgetown University (NIH/NCI grant P30-CA051008), the Fisher Center for Hereditary Cancer and Clinical Genomics Research, and Swing Fore the Cure. Ghent University Hospital (G-FAST): Bruce Poppe is a senior clinical investigator of FWO. Mattias Van Heetvelde obtained funding from IWT. Hospital Clinico San Carlos (HCSC): Spanish Ministry of Health PI15/00059, PI16/01292, and CB-161200301 CIBERONC from ISCIII (Spain), partially supported by European Regional Development FEDER funds. Helsinki Breast Cancer Study (HEBCS): Helsinki University Hospital Research Fund, the Finnish Cancer Society and the Sigrid Juselius Foundation. Hereditary Breast and Ovarian cancer study the Netherlands (HEBON): the Dutch Cancer Society grants NKI1998-1854, NKI2004-3088, NKI2007-3756, the Netherlands Organization of Scientific Research grant NWO 91109024, the Pink Ribbon grants 110005 and 2014-187.WO76, the Biobanking and Biomolecular Resources Research Infrastructure (BBMRI) grant NWO 184.021.007/CP46 and the Transcan grant JTC 2012 Cancer 12-054. HEBON thanks the registration teams of Dutch Cancer Registry (IKNL; S. Siesling, J. Verloop) and the Dutch Pathology database (PALGA; L. Overbeek) for part of the data collection. Study of Genetic Mutations in Breast and Ovarian Cancer patients in Hong Kong and Asia (HRBCP): Hong Kong Sanatorium and Hospital, Dr Ellen Li Charitable Foundation, The Kerry Group Kuok Foundation, National Institute of Health1R 03CA130065, and North California Cancer Center. Molecular Genetic Studies of Breast- and Ovarian Cancer in Hungary (HUNBOCS): Hungarian Research Grants KTIA-OTKA CK-80745 and NKFI_OTKA K-112228. Institut Català d’Oncologia (ICO): The authors would like to particularly acknowledge the support of the Asociación Española Contra el Cáncer (AECC), the Instituto de Salud Carlos III (organismo adscrito al Ministerio de Economía y Competitividad) and “FEDER, una manera de hacer Europa” (PI10/01422, PI13/00285, PIE13/00022, PI15/00854, PI16/00563 and CIBERONC) and the Institut Català de la Salut and Autonomous Government of Catalonia (2009SGR290, 2014SGR338 and PERIS Project MedPerCan). International Hereditary Cancer Centre (IHCC): PBZ_KBN_122/P05/2004. Iceland Landspitali – University Hospital (ILUH): Icelandic Association “Walking for Breast Cancer Research” and by the Landspitali University Hospital Research Fund. INterdisciplinary HEalth Research Internal Team BReast CAncer susceptibility (INHERIT): Canadian Institutes of Health Research for the “CIHR Team in Familial Risks of Breast Cancer” program—grant No. CRN-87521 and the Ministry of Economic Development, Innovation and Export Trade—grant No. PSR-SIIRI-701. Istituto Oncologico Veneto (IOVHBOCS): Ministero della Salute and “5x1000” Istituto Oncologico Veneto grant. Portuguese Oncology Institute-Porto Breast Cancer Study (IPOBCS): Liga Portuguesa Contra o Cancro. Kathleen Cuningham Consortium for Research into Familial Breast Cancer (kConFab): The National Breast Cancer Foundation, and previously by the National Health and Medical Research Council (NHMRC), the Queensland Cancer Fund, the Cancer Councils of New South Wales, Victoria, Tasmania and South Australia, and the Cancer Foundation of Western Australia. Korean Hereditary Breast Cancer Study (KOHBRA): the Korea Health Technology R&D Project through the Korea Health Industry Development Institute (KHIDI), and the National R&D Program for Cancer Control, Ministry of Health & Welfare, Republic of Korea (HI16C1127; 1020350; 1420190). Mayo Clinic (MAYO): NIH grants CA116167, CA192393 and CA176785, an NCI Specialized Program of Research Excellence (SPORE) in Breast Cancer (CA116201), and a grant from the Breast Cancer Research Foundation. McGill University (MCGILL): Jewish General Hospital Weekend to End Breast Cancer, Quebec Ministry of Economic Development, Innovation and Export Trade. Marc Tischkowitz is supported by the funded by the European Union Seventh Framework Program (2007Y2013)/European Research Council (Grant No. 310018). Modifier Study of Quantitative Effects on Disease (MODSQUAD): MH CZ—DRO (MMCI, 00209805), MEYS—NPS I—LO1413 to LF, and by Charles University in Prague project UNCE204024 (MZ). Memorial Sloane Kettering Cancer Center (MSKCC): the Breast Cancer Research Foundation, the Robert and Kate Niehaus Clinical Cancer Genetics Initiative, the Andrew Sabin Research Fund and a Cancer Center Support Grant/Core Grant (P30 CA008748). Women’s College Research Institute Hereditary Breast and Ovarian Cancer Study (NAROD): 1R01 CA149429-01. National Cancer Institute (NCI): the Intramural Research Program of the US NCI, NIH, and by support services contracts NO2-CP-11019-50, N02-CP-21013-63 and N02-CP-65504 with Westat, Inc, Rockville, MD. National Israeli Cancer Control Center (NICCC): Clalit Health Services in Israel, the Israel Cancer Association and the Breast Cancer Research Foundation (BCRF), NY. N.N. Petrov Institute of Oncology (NNPIO): the Russian Foundation for Basic Research (grants 17-54-12007, 17-00-00171 and 18-515-12007). NRG Oncology: U10 CA180868, NRG SDMC grant U10 CA180822, NRG Administrative Office and the NRG Tissue Bank (CA 27469), the NRG Statistical and Data Center (CA 37517) and the Intramural Research Program, NCI. The Ohio State University Comprehensive Cancer Center (OSUCCG): Ohio State University Comprehensive Cancer Center. Università di Pisa (PBCS): AIRC [IG 2013 N.14477] and Tuscany Institute for Tumors (ITT) grant 2014-2015-2016. South East Asian Breast Cancer Association Study (SEABASS): Ministry of Science, Technology and Innovation, Ministry of Higher Education (UM.C/HlR/MOHE/06) and Cancer Research Initiatives Foundation. Sheba Medical Centre (SMC): the Israeli Cancer Association. Swedish Breast Cancer Study (SWE-BRCA): the Swedish Cancer Society. University of Chicago (UCHICAGO): NCI Specialized Program of Research Excellence (SPORE) in Breast Cancer (CA125183), R01 CA142996, 1U01CA161032 and by the Ralph and Marion Falk Medical Research Trust, the Entertainment Industry Fund National Women’s Cancer Research Alliance and the Breast Cancer research Foundation. OIO is an American Cancer Society (ACS) Clinical Research Professor. University of California Los Angeles (UCLA): Jonsson Comprehensive Cancer Center Foundation; Breast Cancer Research Foundation. University of California San Francisco (UCSF): UCSF Cancer Risk Program and Helen Diller Family Comprehensive Cancer Center. UK Familial Ovarian Cancer Registry (UKFOCR): Cancer Research UK. University of Pennsylvania (UPENN): NIH (R01-CA102776 and R01-CA083855); Breast Cancer Research Foundation; Susan G. Komen Foundation for the cure, Basser Research Center for BRCA. Cancer Family Registry University of Pittsburg (UPITT/MWH): Hackers for Hope Pittsburgh. Victorian Familial Cancer Trials Group (VFCTG): Victorian Cancer Agency, Cancer Australia, National Breast Cancer Foundation. Women’s Cancer Program at Cedars-Sinai Medical Center (WCP): Dr Karlan is funded by the ACS Early Detection Professorship (SIOP-06-258-01-COUN) and the National Center for Advancing Translational Sciences (NCATS), Grant UL1TR000124. TN-D is a recipient of a Career Development Fellow from the National Breast Cancer Foundation (Australia, ECF-17-001).

## Notes

**Role of the funders**: The study sponsors had no role in the design of the study; the collection, analysis, and interpretation of the data; the writing of the manuscript; and the decision to submit the manuscript for publication.

**Disclosures:** ILA has received funding from the NIH. NA has received lecture fees from AstraZeneca and Clovis Oncology. ÅB has received personal honoraria for lectures at courses in tumor biology and genetics for medical students and physicians, courses organized by AstraZeneca and Roche. LC has received honoraria from AstraZeneca, MSD, Pfizer and Novartis. SMD has received honoraria from AstraZeneca. CE received funding from German Cancer Aid. DGE has received honoraria from AstraZeneca, Springworks and Cerexis. AKG has received funding from the NIH, NCI, and NIGMS and honoraria from VITRAC Therapeutics and NanoString Technologies, and is co-founder of Sinochips Diagnostics. TVOH has received lecture honoraria from Pfizer. GK received advisory board honoraria from AstraZeneca, Sanofi-Aventis, Janssen, Bayer, AMGEN, Ferring and Astellas. H.N has funding from the Helsinki University Hospital Research Fund, The Sigrid Juselius Foundation, The Finnish Cancer Society and honoraria from AstraZeneca. OIO is co-Founder of Cancer IQ and serves on the boards of 54gene and Tempus. ZS’s immediate family member received consulting fees from Genentech/Roche, Novartis, RegenexBio, Neurogene, Optos Plc, Regeneron, Allergan, Gyroscope Tx and Adverum. L.S has received funding from the NCI paid to institution. AET has received funding from the NCI paid to institution. ATo has received honoraria from Lilly, Roche, Novartis and MSD. JV has received funding from the Breast Cancer Research Foundation. FJC has received funding from the NIH and the Breast Cancer Research Foundation paid to institution. RKS has received funding from German Cancer Aid. JS has received funding from the Government of Canada through Genome Canada and the Canadian Institutes of Health Research, the Ministère de l’Économie et de l’Innovation du Québec through Genome Québec, the Quebec Breast Cancer Foundation, the CHU de Quebec Foundation, and the Ontario Research Fund. DFE has received funding from Cancer Research UK paid to institution. ACA is listed as creator of the BOADICEA algorithm, which has been licensed to Cambridge Enterprise. LO has received funding from the Italian Association for Cancer Research. All other authors have no disclosures.

**Author contributions:** Conceptualization: GC-T, ACA and LO. Data curation: DRB, GL, LMc, JD and XY. Formal analysis: DRB and VS. Funding acquisition: KO, MTh, FJC, RKS, JS, DFE, GC-T, ACA and LO. Investigation: DRB, VS, JAd, BAA, MA, KA, ILA, AA, NA, BA, JAz, JBal, RBB, DB, JBar, MB, JBe, PBe, SEB, ÅB, ABo, ABr, PBr, CB, JBr, ABu, SSB, TC, MAC, IC, HC, LLC, GC, KBMC, JC, AC, LC, GD, ED, RD, MDLH, KDL, RDP, JDV, OD, YCD, SMD, AD, JE, RE, CE, DGE, LF, FF, MF, DF, DG, AG, SG, GG, AKG, DEG, MHG, HG, EG, EH, UH, TVOH, HH, JHe, JHo, LI, AI, PAJ, RJ, UBJ, OTJ, EMJ, GK, LK, TAK, CLau, CLaz, FL, AL-F, PLM, SM, ZM, LMa, KNM, NM, AMe, MM, ANM, PJM, TAM, AMu, KLN, SLN, HN, TN-D, DN, EO, OIO, DP, MTP, ISP, BP, PP-S, PPe, AHP, PPi, MEP, CP, MAP, PR, JRam, JRan, MR, MTR, KR, AR, AMSDA, PDS, SS, LES, CFS, ZS, LS, DS-L, CS, YYT, MRT, ATe, DLT, MTi, AET, ST, ATo, AHT, VT, VV, CJVA, MV, AV, JV, LW, SW-G, BW, AW, IZ, KO, MTh, FJC, RKS, JS, DFE, GC-T, ACA and LO. Methodology: DRB, VS, ACA and LO. Project administration: GL, LMc, ACA and LO. Resources: JAd, BAA, MA, KA, ILA, AA, NA, BA, JAz, JBal, RBB, DB, JBar, MB, JBe, PBe, SEB, ÅB, ABo, ABr, PBr, CB, JBr, ABu, SSB, TC, MAC, IC, HC, LLC, GC, KBMC, JC, AC, LC, GD, ED, RD, MDLH, KDL, RDP, JDV, OD, YCD, SMD, AD, JE, RE, CE, DGE, LF, FF, MF, DF, DG, AG, SG, GG, AKG, DEG, MHG, HG, EG, EH, UH, TVOH, HH, JHe, JHo, LI, AI, PAJ, RJ, UBJ, OTJ, EMJ, GK, LK, TAK, CLau, CLaz, FL, AL-F, PLM, SM, ZM, LMa, KNM, NM, AMe, MM, ANM, PJM, TAM, AMu, KLN, SLN, HN, TN-D, DN, EO, OIO, DP, MTP, ISP, BP, PP-S, PPe, AHP, PPi, MEP, CP, MAP, PR, JRam, JRan, MR, MTR, KR, AR, AMSDA, PDS, SS, LES, CFS, ZS, LS, DS-L, CS, YYT, MRT, ATe, DLT, MTi, AET, ST, ATo, AHT, VT, VV, CJVA, MV, AV, JV, LW, SW-G, BW, AW, IZ, KO, MTh, FJC, RKS, JS, DFE, GC-T, ACA and LO. Software: DRB. Supervision: ACA and LO. Visualization: DRB. Writing—original draft: DRB, VS, ACA and LO. Writing—review & editing: DRB, VS, GL, LMc, JD, XY, JAd, BAA, MA, KA, ILA, AA, NA, BA, JAz, JBal, RBB, DB, JBar, MB, JBe, PBe, SEB, ÅB, ABo, ABr, PBr, CB, JBr, ABu, SSB, TC, MAC, IC, HC, LLC, GC, KBMC, JC, AC, LC, GD, ED, RD, MDLH, KDL, RDP, JDV, OD, YCD, SMD, AD, JE, RE, CE, DGE, LF, FF, MF, DF, DG, AG, SG, GG, AKG, DEG, MHG, HG, EG, EH, UH, TVOH, HH, JHe, JHo, LI, AI, PAJ, RJ, UBJ, OTJ, EMJ, GK, LK, TAK, CLau, CLaz, FL, AL-F, PLM, SM, ZM, LMa, KNM, NM, AMe, MM, ANM, PJM, TAM, AMu, KLN, SLN, HN, TN-D, DN, EO, OIO, DP, MTP, ISP, BP, PP-S, PPe, AHP, PPi, MEP, CP, MAP, PR, JRam, JRan, MR, MTR, KR, AR, AMSDA, PDS, SS, LES, CFS, ZS, LS, DS-L, CS, YYT, MRT, ATe, DLT, MTi, AET, ST, ATo, AHT, VT, VV, CJVA, MV, AV, JV, LW, SW-G, BW, AW, IZ, KO, MTh, FJC, RKS, JS, DFE, GC-T, ACA and LO.

## Supplementary Material

djab147_Supplementary_Data
