# Supplementary material for: Breast and Prostate Cancer Risks for Male BRCA1 and BRCA2 Pathogenic Variant Carriers Using Polygenic Risk Scores
Source: J Natl Cancer Inst. 2021 Jul 28;114(1):109–22. doi: 10.1093/jnci/djab147 (PMC8755508; doi:10.1093/jnci/djab147)
Supplement: djab147_Supplementary_Data [file djab147_supplementary_data.docx]

**SUPPLEMENTARY MATERIAL**

**Supplementary Methods**

**Study participants and genotyping**
1,989 male *BRCA1* and *BRCA2* pathogenic variant carriers recruited by the Consortium of Investigators of Modifiers of *BRCA1* and *BRCA2* (CIMBA)^1^ were selected for genotyping from a case-control study (every breast and/or prostate cancer case matched with up to three controls), matching on study or country, year of birth (within ±5 years) and *BRCA1* or *BRCA2* carrier status. Most of the men were recruited through cancer genetics clinics (96.9%, Supplementary Table 1)^2^. Genotyping was performed using the OncoArray, a custom genotyping array of approximately 533,000 SNPs including GWAS backbone tagging common SNPs^3^. At the time of sample selection for genotyping, the number of non-European ancestry male carriers in CIMBA was very small to allow any meaningful analyses. Therefore, sample selection was restricted to carriers of self-reported European ancestry. During the quality control process, male carriers of non-European genetic ancestry were also excluded from statistical analyses. Genetic ancestry was determined using multidimensional scaling based on 30,733 uncorrelated (r^2^<0.10) autosomal SNPs to calculate the kinship between all *BRCA1* and *BRCA2* carriers and 267 samples from HapMap (CHB, JPT, YRI and CEU populations). These were converted to distances and underwent multidimensional scaling. Using the first two components, the proportion of European ancestry was calculated for each individual and samples with >27% non-European ancestry were excluded. Two-stage imputation was undertaken using SHAPEIT^4^ for phasing and IMPUTE2^5^ for imputation using the 1000 Genomes Project (Phase 3) reference panel. Following quality control a total of 483 *BRCA1* and 1,318 *BRCA2* pathogenic variant carriers of European ancestry were eligible for inclusion in the analysis.

SNPs were included in the PRS if they were adequately imputed in the CIMBA data. The imputation accuracy was assessed using the *r*^2^ statistic, based on the “info” statistic produced by the IMPUTE2 software (<https://mathgen.stats.ox.ac.uk/impute/impute_v2.html#info_metric_details>)^5^. This statistic takes values from 0 (complete uncertainty of imputed genotypes) to 1 (no uncertainty of imputed genotypes). The minimum *r*^2^ value for the 313-SNP breast cancer PRS^6^ SNPs was 0.86 (Supplementary Table 3, Supplementary Figure 1) and the minimum *r*^2^ for the 147-SNP prostate cancer PRS^7^ SNPs was 0.76 (Supplementary Table 4 and Supplementary Figure 2).

**Principal components analysis**

To adjust for potential (intra-continental) population stratification, principal components analysis was performed using data from 33,661 uncorrelated SNPs (which included 2,318 SNPs specifically selected on informativeness for determining continental ancestry) with an allele frequency of ≥0.05 and a maximum correlation of 0.1 in the OncoArray dataset, using purpose-written software (<http://ccge.medschl.cam.ac.uk/software/pccalc>).

**Calculation of the breast cancer and prostate cancer PRS**

The PRS for each individual, *i*, can be expressed as:

$${PRS}_{i} = \sum_{j=1}^{N} \beta_{j}g_{ij}$$

where *g_ij_* is the genotype or imputed dosage for SNP *j* observed for participant *i*. Each β*_j_* is the weight for the *j*^th^ SNP.

The weights for the breast cancer PRS were the log Odds Ratios (log-OR) estimates of association used to construct the 313-SNP PRS based on female population-based data from BCAC^6^ (Supplementary Table 3).

The weights for the prostate cancer PRS were the log-OR estimated of association used to form the 147-SNP PRS based on data from the general population from PRACTICAL^7^ (Supplementary Table 4).

The three breast cancer PRS were scaled to the female population-based PRS standard deviations calculated in controls (PRS_BC_ SD=0.609; PRS_ER-_ SD=0.592; PRS_ER+_ SD=0.652)^6^, allowing for direct comparison of association estimates with previous investigations^6,8,9^. The prostate cancer PRS was scaled to the male population-based standard deviation calculated in controls (SD=0.689)^7^, making the estimated associations directly comparable with previous studies^7^.

**Description of the statistical models**

***Multinomial logistic regression.*** The case-control study association analyses were performed using multinomial logistic regression. These analyses considered controls, breast cancer cases and prostate cancer cases simultaneously as the outcome of interest. These models estimated the association of the PRS with breast cancer risk and prostate cancer risk simultaneously. All models were adjusted for the first three principal components and age at cancer diagnosis (breast cancer or prostate cancer, whichever occurred first) or age at interview for controls. In the analyses combining the *BRCA1* and *BRCA2* carriers, a further adjustment was made for the gene pathogenic variant carrier status: either a *BRCA1* pathogenic variant carrier or a *BRCA2* pathogenic variant carrier. Cluster robust variances were estimated to account for related individuals. Multinomial logistic regression models were fitted using the mlogit command from the R “mlogit” library.

***Adjusting models for family history of cancer in first- and second-degree relatives.*** Considering breast cancer as the primary outcome of interest, we fitted models adjusting for family history of: (i) male breast cancer; and (ii) female breast cancer. When considering prostate cancer as the disease of interest, we adjusted for family history of prostate cancer when considering prostate cancer as the primary outcome. Family history was categorized as: “no family history”, “one or more relatives with cancer”, “unknown” or “missing”.

***BRCA1 and BRCA2 pathogenic variant locations.*** For the breast cancer PRS *BRCA1* pathogenic variants were grouped into three regions (5’ to c.2281, c.2282 to c.4071 and c.4072 to 3’)^10,11^. *BRCA2* pathogenic variants were grouped using the “wide” (5’ to c.2831, c.2832 to c.6401, c.6402 to 3’) ovarian cancer cluster region (OCCR) definition^10,12^. For the PRS_PC_, we used the *BRCA2* prostate cancer cluster regions (PCCR)^13^: 5’ to c.755, c.756 to c.1000, c.1001 to c.7913 and c.7914 to 3’.

***Sensitivity analysis: Heterogeneity in PRS association by country.*** We were unable to assess study specific PRS associations (small number of controls or cases within each study, Supplementary Table 1). Therefore, we instead evaluated the evidence for PRS association heterogeneity by country. Due to the small number of samples for specific countries, we also grouped some countries together, based on geographical location. For *BRCA1* carriers we created the following groups of countries: (a) Germany, Austria, Hungary, France and the Netherlands; (b) Greece, Italy and Spain; (c) UK, Denmark, Finland, Sweden, Latvia and Lithuania; (d) USA and Canada and (e) Australia. For *BRCA2* carriers we used: (a) Belgium and France; (b) Finland, Sweden and Denmark; (d) Germany, Hungary, Austria and the Netherlands; (d) Greece and Italy; (e) Spain and Portugal; and (f) USA and Canada. Australia, Iceland and UK formed individual strata.

To assess the evidence of heterogeneity in PRS associations we fit two models: (i) a model estimating the main effect of the PRS and a PRSxCountry interaction term; and (ii) a nested model estimating the main effect of the PRS with no interaction term. Models (i) and (ii) were then compared using a likelihood ratio test (description below).

***Model comparisons.*** Likelihood ratio tests (LRTs) were undertaken to determine whether models which included interaction terms (PRS interaction with age, PRS interaction with gene pathogenic variant location, PRS interaction with gene pathogenic variant class, and country) fitted data better than the nested model that did not include the interaction term. Here we considered two models: (i) a model that includes the PRS interaction term, with a corresponding log-likelihood, *L_I_* and the nested model without the interaction term with log-likelihood *L_N_*. Hence, the LRT comparing these models has the form:

$$-2[L_{N}-L_{I}] \sim\chi_{\Delta d}^{2}$$

where Δ*d* is the degrees of freedom for the χ^2^ test, given by the difference in the number of parameters estimated in each model.

**Calculating absolute risks of cancer by PRS percentiles**

Breast cancer and prostate cancer absolute risks were calculated according to percentiles of the relevant PRS, following a previously published method^14^. For these calculations we assumed external estimates of male breast cancer incidence and prostate cancer incidence. Average age-specific cancer incidences were constrained over PRS percentile categories to agree with the external cancer incidences for male *BRCA1* and *BRCA2* carriers^15-17^. We applied the estimated ORs from the combined sample of *BRCA1* and *BRCA2* carriers to these assumed cancer incidence rates (Supplementary Tables 4, Supplementary Table 5). The combined *BRCA1*/*2* carrier sample OR estimates were chosen to ensure estimates were taken from the model with greatest stability from the increased sample size of combining the *BRCA1* and *BRCA2* carriers into a single model. Furthermore, the estimated ORs from the combined sample model (Supplementary Tables 4 and 5) were consistent with models fitted to *BRCA1* and *BRCA2* carriers separately (Table 1, Table 2).

We assumed male breast cancer age-specific population-based incidences applicable to a man born in the UK in 1985^17^. To obtain incidences applicable to *BRCA1* and *BRCA2* carriers, we multiplied the population-based incidence rates relative risks (RR) estimates of developing male breast cancer for *BRCA1* and *BRCA2* carriers relative to the general population as previously estimated by the Breast Cancer Linkage Consortium (BCLC). A constant RR=8 across the age range up to age 85-years was used for *BRCA1* carriers^18^ [Breast Cancer Linkage Consortium]. The corresponding RR for *BRCA2* carriers was RR=80^12^. These assumed breast cancer incidences for male *BRCA1*/*2* carriers are consistent with previous PRS risk prediction analyses for male carriers^16^.

The assumed prostate cancer incidence rates were those estimated from a prospective cohort of *BRCA1* and *BRCA2* carriers^15^. We assumed the incidence from Nyberg et al that were estimated in age groups 19-44, 45-54, 55-64, 65-74 and 75-84. Incidence rates of zero were assumed from birth to age 18-years. We further assumed prostate cancer incidence rates applicable to the general population (UK Office for National Statistics, 2017 Cancer registration statistics for England)^19^ for ages 35-39 (rate: 0.4 per 100,000) and 40-44 (rate: 4 per 100,000), in place of the estimated incidence of zero by Nyberg et al^15^, which is likely an underestimate for carriers in these age groups as it is lower than the observed population incidence rates.

**Calculating ten-year risks of cancer by PRS percentile**

The ten-year risks, *R*(*t*)_10_, of developing male breast cancer and prostate cancer at age *t* was calculated as the risk difference between ages (*t*+10) and *t*, conditional on not developing cancer up to age *t*. This can be written as:

$${R(t)}_{10}=\frac{P(t+10) - P(t)}{1 - P(t)}$$

where *P*(*t*) is the cumulative disease risk at age *t* and is calculated using PRS specific incidences calculated in the previous section.

**Statistical software (R) commands used for statistical analyses**

*Function to calculate cluster robust variance estimates with R “mlogit” command*

# Input requires “mod”, a “mlogit” model

# To calculate cluster robust variance, provide clustering variable “clust”

proc.mlogit <- function(mod,clust=NULL,ci=95) {

require(mlogit)

require(sandwich)

require(lmtest)

# Checks

stopifnot(class(mod)=="mlogit")

stopifnot(class(ci)%in%c("numeric","integer"))

stopifnot(ci>0 & ci<100)

# Confidence level

ci <- ((100-ci)/100)/2

# Cluster robust variance

if(!is.null(clust)) {

m <- length(unique(clust))

n <- length(clust)

k <- length(mod$coefficients)

dfc <- m/(m-1)

uj <- apply(estfun(mod),2, function(x) tapply(x, clust, sum));

cl.vcov <- dfc*sandwich(mod, meat.=crossprod(uj)/n)

cl <- coeftest(mod,cl.vcov)

cl <- data.frame(cl[,1:2])

colnames(cl) <- c("beta","robust.se")

cl$or <- exp(cl$beta)

cl$lci <- exp(cl$beta-abs(qnorm(ci)*cl$robust.se))

cl$uci <- exp(cl$beta+abs(qnorm(ci)*cl$robust.se))

cl$p <- 2*pnorm(abs(cl$beta/cl$robust.se),lower.tail=FALSE)

} else {

cl <- NA

cl.vcov <- vcov(mod)

}

m <- data.frame(summary(mod)$CoefTable[,1:2])

colnames(m) <- c("beta","se")

m$or <- exp(m$beta)

m$lci <- exp(m$beta-abs(qnorm(ci)*m$se))

m$uci <- exp(m$beta+abs(qnorm(ci)*m$se))

m$p <- 2*pnorm(abs(m$beta/m$se),lower.tail=FALSE)

return(list(standard = m, cluster.robust = cl, var.covar = cl.vcov))

}

*Multinomial logistic regression*
library(mlogit)

mdat <- mlogit.data(dat, shape = "wide", choice = "mult.cens.var")

clust <- dat$FAMILY
model <- proc.mlogit(mlogit(MULT.CENSVAR ~ 0 | NORMALISED.PRS + PC1 + PC2 + PC3 + AGE + BRCA, data = mdat), clust))

*Discrimination analyses: calculating the area under the receiver operator characteristic curve*

library(pROC)
# set “c” according to which cancer to exclude from the AUC calculation

# 1 = exclude breast cancer cases / 2 = exclude prostate cancer cases

# The AUC then applies to the alternate cancer versus controls
c <- 1
roc.censvar <- dat[which(dat$MULT.CENSVAR != c),]$MULT.CENSVAR

roc.prs <- dat[which(dat$MULT.CENSVAR != c),]$NORMALISED.PRS

auc <- roc(roc.censvar, roc.prs, direction = "auto")

**References**

1. Chenevix-Trench G, Milne RL, Antoniou AC, et al. An international initiative to identify genetic modifiers of cancer risk in BRCA1 and BRCA2 mutation carriers: the Consortium of Investigators of Modifiers of BRCA1 and BRCA2 (CIMBA). *Breast Cancer Res.* 2007;9(2):104.

2. Silvestri V, Barrowdale D, Mulligan AM, et al. Male breast cancer in BRCA1 and BRCA2 mutation carriers: pathology data from the Consortium of Investigators of Modifiers of BRCA1/2. *Breast Cancer Res.* 2016;18(1):15.

3. Amos CI, Dennis J, Wang Z, et al. The OncoArray Consortium: A Network for Understanding the Genetic Architecture of Common Cancers. *Cancer Epidemiol Biomarkers Prev.* 2017;26(1):126-135.

4. Delaneau O, Marchini J, Zagury JF. A linear complexity phasing method for thousands of genomes. *Nat Methods.* 2011;9(2):179-181.

5. Howie BN, Donnelly P, Marchini J. A flexible and accurate genotype imputation method for the next generation of genome-wide association studies. *PLoS Genet.* 2009;5(6):e1000529.

6. Mavaddat N, Michailidou K, Dennis J, et al. Polygenic Risk Scores for Prediction of Breast Cancer and Breast Cancer Subtypes. *Am J Hum Genet.* 2019;104(1):21-34.

7. Schumacher FR, Al Olama AA, Berndt SI, et al. Association analyses of more than 140,000 men identify 63 new prostate cancer susceptibility loci. *Nat Genet.* 2018;50(7):928-936.

8. Barnes DR, Rookus MA, McGuffog L, et al. Polygenic risk scores and breast and epithelial ovarian cancer risks for carriers of BRCA1 and BRCA2 pathogenic variants. *Genet Med.* 2020;22(10):1653-1666.

9. Maguire S, Perraki E, Tomczyk K, et al. Common susceptibility loci for male breast cancer. *J Natl Cancer Inst.* 2020;113(4).

10. Rebbeck TR, Mitra N, Wan F, et al. Association of type and location of BRCA1 and BRCA2 mutations with risk of breast and ovarian cancer. *JAMA.* 2015;313(13):1347-1361.

11. Thompson D, Easton D, Breast Cancer Linkage C. Variation in BRCA1 cancer risks by mutation position. *Cancer Epidemiol Biomarkers Prev.* 2002;11(4):329-336.

12. Thompson D, Easton D, Breast Cancer Linkage C. Variation in cancer risks, by mutation position, in BRCA2 mutation carriers. *Am J Hum Genet.* 2001;68(2):410-419.

13. Patel VL, Busch EL, Friebel TM, et al. Association of Genomic Domains in BRCA1 and BRCA2 with Prostate Cancer Risk and Aggressiveness. *Cancer Res.* 2020;80(3):624-638.

14. Antoniou AC, Beesley J, McGuffog L, et al. Common breast cancer susceptibility alleles and the risk of breast cancer for BRCA1 and BRCA2 mutation carriers: implications for risk prediction. *Cancer Res.* 2010;70(23):9742-9754.

15. Nyberg T, Frost D, Barrowdale D, et al. Prostate Cancer Risks for Male BRCA1 and BRCA2 Mutation Carriers: A Prospective Cohort Study. *Eur Urol.* 2020;77(1):24-35.

16. Lecarpentier J, Silvestri V, Kuchenbaecker KB, et al. Prediction of Breast and Prostate Cancer Risks in Male BRCA1 and BRCA2 Mutation Carriers Using Polygenic Risk Scores. *J Clin Oncol.* 2017;35(20):2240-2250.

17. Lee A, Mavaddat N, Wilcox AN, et al. BOADICEA: a comprehensive breast cancer risk prediction model incorporating genetic and nongenetic risk factors. *Genet Med.* 2019;21(8):1708-1718.

18. Antoniou AC, Cunningham AP, Peto J, et al. The BOADICEA model of genetic susceptibility to breast and ovarian cancers: updates and extensions. *Br J Cancer.* 2008;98(8):1457-1466.

19. ONS. Cancer registration statistics, England <https://www.ons.gov.uk/peoplepopulationandcommunity/healthandsocialcare/conditionsanddiseases/datasets/cancerregistrationstatisticscancerregistrationstatisticsengland>. Published 2019. Accessed 9th October, 2020.

**Supplementary Tables**

**Supplementary Table 1**: Description of participants included in statistical analyses by study.

| Study | Country | Clinic-based  Ascertainment, %^a^ | *BRCA1* carriers | | | | *BRCA2* carriers | | | |
| --- | --- | --- | --- | --- | --- | --- | --- | --- | --- | --- |
|  |  |  | Controls | Breast cancer cases | Prostate cancer cases | Controls and cases | Controls | Breast cancer cases | Prostate cancer cases | Controls and cases |
| BCFR-AU | Australia | 0.0 | 0 | 0 | 0 | 0 | 0 | 0 | 1 | 1 |
| BCFR-NC | USA | 0.0 | 2 | 0 | 0 | 2 | 5 | 0 | 0 | 5 |
| BCFR-ON | Canada | 16.7 | 5 | 1 | 1 | 7 | 6 | 3 | 2 | 11 |
| BCFR-UT | USA | 100.0 | 11 | 1 | 2 | 14 | 24 | 1 | 3 | 28 |
| BFBOCC | Latvia/Lithuania | 100.0 | 3 | 0 | 1 | 4 | 0 | 0 | 0 | 0 |
| BRICOH | USA | 100.0 | 11 | 1 | 6 | 18 | 36 | 16 | 8 | 60 |
| CBCS | Denmark | 100.0 | 1 | 0 | 0 | 1 | 0 | 0 | 0 | 0 |
| CNIO | Spain | 100.0 | 1 | 0 | 0 | 1 | 3 | 0 | 0 | 3 |
| CONSIT TEAM | Italy | 91.3 | 77 | 2 | 0 | 79 | 64 | 26 | 4 | 94 |
| DEMOKRITOS | Greece | 100.0 | 1 | 0 | 1 | 2 | 0 | 1 | 0 | 1 |
| DKFZ | Germany | 100.0 | 5 | 0 | 0 | 5 | 1 | 0 | 0 | 1 |
| EMBRACE | UK | 100.0 | 58 | 4 | 15 | 77 | 162 | 25 | 34 | 221 |
| FCCC | USA | 0.00 | 1 | 0 | 1 | 2 | 11 | 2 | 2 | 15 |
| G-FAST | Belgium | 100.0 | 0 | 0 | 0 | 0 | 24 | 6 | 0 | 30 |
| GC-HBOC | Germany | 100.0 | 28 | 9 | 4 | 41 | 74 | 30 | 5 | 109 |
| GEMO | France | 100.0 | 7 | 0 | 3 | 10 | 43 | 10 | 5 | 58 |
| HCSC | Spain | 100.0 | 0 | 0 | 0 | 0 | 28 | 6 | 3 | 37 |
| HEBCS | Finland | 100.0 | 5 | 0 | 1 | 6 | 20 | 0 | 5 | 25 |
| HEBON | Netherlands | 100.0 | 6 | 0 | 2 | 8 | 7 | 0 | 0 | 7 |
| HUNBOCS | Hungary | 100.0 | 6 | 2 | 0 | 8 | 4 | 13 | 1 | 18 |
| HVH | Spain | 100.0 | 1 | 0 | 1 | 2 | 10 | 7 | 1 | 18 |
| ICO | Spain | 100.0 | 7 | 0 | 0 | 7 | 52 | 8 | 4 | 64 |
| ILUH | Iceland | 100.0 | 0 | 0 | 0 | 0 | 26 | 9 | 7 | 42 |
| IOVHBOCS | Italy | 100.0 | 0 | 0 | 1 | 1 | 14 | 5 | 1 | 20 |
| IPOBCS | Portugal | 100.0 | 0 | 0 | 0 | 0 | 9 | 2 | 1 | 12 |
| KCONFAB | Australia | 100.0 | 40 | 5 | 9 | 54 | 128 | 24 | 28 | 180 |
| MAYO | USA | 100.0 | 2 | 0 | 0 | 2 | 1 | 1 | 0 | 2 |
| MSKCC | USA | 100.0 | 11 | 1 | 2 | 14 | 27 | 3 | 7 | 37 |
| MUV | Austria | 100.0 | 3 | 0 | 1 | 4 | 10 | 7 | 1 | 18 |
| NCI | USA | 100.0 | 5 | 1 | 1 | 7 | 7 | 1 | 0 | 8 |
| OCGN | Canada | 100.0 | 4 | 0 | 2 | 6 | 9 | 3 | 0 | 12 |
| OSU CCG | USA | 100.0 | 3 | 0 | 1 | 4 | 7 | 2 | 0 | 9 |
| OUH | Denmark | 100.0 | 19 | 2 | 5 | 26 | 60 | 10 | 8 | 78 |
| PBCS | Italy | 100.0 | 0 | 0 | 0 | 0 | 1 | 3 | 0 | 4 |
| SWE-BRCA | Sweden | 100.0 | 6 | 0 | 2 | 8 | 2 | 0 | 1 | 3 |
| UCHICAGO | USA | 100.0 | 4 | 0 | 3 | 7 | 9 | 2 | 2 | 13 |
| UPENN | USA | 100.0 | 36 | 3 | 3 | 42 | 31 | 10 | 3 | 44 |
| UPITT | USA | 100.0 | 0 | 0 | 1 | 1 | 3 | 7 | 1 | 11 |
| VFCTG | Australia | 100.0 | 11 | 1 | 1 | 13 | 15 | 1 | 3 | 19 |

^a^ Clinic-based ascertainment (%) = percentage of participants ascertained through cancer genetics clinics, as opposed to population-based ascertainment.

**Supplementary Table 2**: Case-control study characteristics for 483 *BRCA1* and 1,318 *BRCA2* male carriers.

| Characteristic | *BRCA1* carriers | | | | *BRCA2* carriers | | | | *BRCA1* and *BRCA2* carriers | | | |
| --- | --- | --- | --- | --- | --- | --- | --- | --- | --- | --- | --- | --- |
|  | Controls  (n = 380) | Breast cancer cases  (n = 33) | Prostate cancer cases  (n = 70) | Controls and cases  (n = 483) | Controls  (n = 933) | Breast cancer cases  (n = 244) | Prostate cancer cases  (n = 141) | Controls and cases  (n = 1318) | Controls  (n = 1313) | Breast cancer cases  (n = 277) | Prostate cancer cases  (n = 211) | Controls and cases  (n = 1801) |
| Age |  |  |  |  |  |  |  |  |  |  |  |  |
| Median (IQR), years | 64.1 (50.0-73.0) | 65.0 (56.6-71.0) | 67.5 (59.9-71.2) | 65.0 (53.0-72.9) | 57.0 (48.0-67.0) | 62.8 (54.0-70.0) | 62.2 (56.6-67.3) | 59.0 (50.0-67.3) | 59.0 (48.3-69.0) | 63.0 (54.0-70.0) | 63.0 (57.0-70.0) | 60.6 (50.5-69.3) |
| <30y | 6 | 0 | 0 | 6 | 13 | 0 | 1 | 14 | 19 | 0 | 1 | 20 |
| 30-39 y | 37 | 1 | 0 | 38 | 69 | 6 | 1 | 76 | 106 | 7 | 1 | 114 |
| 40-49 y | 49 | 4 | 1 | 54 | 193 | 26 | 8 | 227 | 242 | 30 | 9 | 281 |
| 50-59 y | 59 | 7 | 17 | 83 | 245 | 65 | 43 | 353 | 304 | 72 | 60 | 436 |
| 60-69 y | 88 | 12 | 27 | 127 | 249 | 81 | 59 | 389 | 337 | 93 | 86 | 516 |
| 70-79 y | 108 | 7 | 21 | 136 | 132 | 60 | 24 | 216 | 240 | 67 | 45 | 352 |
| 80-89 y | 30 | 2 | 4 | 36 | 27 | 6 | 5 | 38 | 57 | 8 | 9 | 74 |
| ≥90 y | 3 | 0 | 0 | 3 | 5 | 0 | 0 | 5 | 8 | 0 | 0 | 8 |
| Pathogenic variant class^a^ |  |  |  |  |  |  |  |  |  |  |  |  |
| I | 280 | 21 | 51 | 352 | 831 | 225 | 123 | 1179 | 1111 | 246 | 174 | 1531 |
| II | 62 | 9 | 12 | 83 | 29 | 6 | 7 | 42 | 91 | 15 | 19 | 125 |
| III | 38 | 3 | 7 | 48 | 73 | 13 | 11 | 97 | 111 | 16 | 18 | 145 |
| *BRCA1* pathogenic variant location (OCCR) |  |  |  |  |  |  |  |  |  |  |  |  |
| 5' to c.2281 | 160 | 9 | 39 | 208 | ─ | ─ | ─ | ─ | ─ | ─ | ─ | ─ |
| c.2282 to c.4071 | 123 | 10 | 17 | 150 | ─ | ─ | ─ | ─ | ─ | ─ | ─ | ─ |
| c.4072 to 3' | 97 | 14 | 14 | 125 | ─ | ─ | ─ | ─ | ─ | ─ | ─ | ─ |
| *BRCA2* pathogenic variant location (narrow OCCR) |  |  |  |  |  |  |  |  |  |  |  |  |
| 5' to c.3846 | ─ | ─ | ─ | ─ | 305 | 81 | 43 | 429 | ─ | ─ | ─ | ─ |
| c.3847 to c.6275 | ─ | ─ | ─ | ─ | 313 | 66 | 45 | 424 | ─ | ─ | ─ | ─ |
| c.6276 to 3' | ─ | ─ | ─ | ─ | 315 | 97 | 53 | 465 | ─ | ─ | ─ | ─ |
| *BRCA2* pathogenic variant location (wide OCCR) |  |  |  |  |  |  |  |  |  |  |  |  |
| 5' to c.2830 | ─ | ─ | ─ | ─ | 263 | 75 | 38 | 376 | ─ | ─ | ─ | ─ |
| c.2831 to c.6401 | ─ | ─ | ─ | ─ | 363 | 76 | 56 | 495 | ─ | ─ | ─ | ─ |
| c.6402 to 3' | ─ | ─ | ─ | ─ | 307 | 93 | 47 | 447 | ─ | ─ | ─ | ─ |
| *BRCA2* pathogenic variant location (PCCR) |  |  |  |  |  |  |  |  |  |  |  |  |
| 5' to c.755 | ─ | ─ | ─ | ─ | 100 | 33 | 15 | 148 | ─ | ─ | ─ | ─ |
| c.756 to c.1000 | ─ | ─ | ─ | ─ | 37 | 12 | 10 | 59 | ─ | ─ | ─ | ─ |
| c.1001 to c.7913 | ─ | ─ | ─ | ─ | 619 | 140 | 81 | 840 | ─ | ─ | ─ | ─ |
| c.7914 to 3' | ─ | ─ | ─ | ─ | 177 | 59 | 35 | 271 | ─ | ─ | ─ | ─ |
| ER-status |  |  |  |  |  |  |  |  |  |  |  |  |
| Negative | ─ | 2 | ─ | ─ | ─ | 7 | ─ | ─ | ─ | 9 | ─ | ─ |
| Positive | ─ | 21 | ─ | ─ | ─ | 178 | ─ | ─ | ─ | 199 | ─ | ─ |
| Missing | ─ | 9 | ─ | ─ | ─ | 58 | ─ | ─ | ─ | 67 | ─ | ─ |
| Gleason score (GS) |  |  |  |  |  |  |  |  |  |  |  |  |
| < 7 | ─ | ─ | 26 | ─ | ─ | ─ | 27 | ─ | ─ | ─ | 53 | ─ |
| ≥ 7 | ─ | ─ | 21 | ─ | ─ | ─ | 82 | ─ | ─ | ─ | 103 | ─ |
| GS unknown | ─ | ─ | 23 | ─ | ─ | ─ | 32 | ─ | ─ | ─ | 55 | ─ |
| Ascertainment |  |  |  |  |  |  |  |  |  |  |  |  |
| Clinic | 371 | 31 | 68 | 470 | 910 | 230 | 136 | 1276 | 1281 | 261 | 204 | 1746 |
| Population-based | 9 | 2 | 2 | 13 | 23 | 14 | 5 | 42 | 32 | 16 | 7 | 55 |
| Family history of breast cancer in male 1st and 2nd degree relatives |  |  |  |  |  |  |  |  |  |  |  |  |
| None | 286 | 24 | 49 | 359 | 585 | 154 | 103 | 842 | 871 | 178 | 152 | 1201 |
| ≥ 1 case | 7 | 0 | 0 | 7 | 128 | 33 | 12 | 173 | 135 | 33 | 12 | 180 |
| Unknown | 26 | 2 | 3 | 31 | 53 | 3 | 9 | 65 | 79 | 5 | 12 | 96 |
| Missing | 61 | 7 | 18 | 86 | 167 | 54 | 17 | 238 | 228 | 61 | 35 | 324 |
| Family history of breast cancer in female 1st and 2nd degree relatives |  |  |  |  |  |  |  |  |  |  |  |  |
| None | 37 | 4 | 5 | 46 | 59 | 43 | 13 | 115 | 96 | 47 | 18 | 161 |
| ≥ 1 case | 277 | 22 | 46 | 345 | 701 | 145 | 109 | 955 | 978 | 167 | 155 | 1300 |
| Unknown | 5 | 0 | 1 | 6 | 6 | 2 | 2 | 10 | 11 | 2 | 3 | 16 |
| Missing | 61 | 7 | 18 | 86 | 167 | 54 | 17 | 238 | 228 | 61 | 35 | 324 |
| Family history of prostate cancer in 1st and 2nd degree relatives |  |  |  |  |  |  |  |  |  |  |  |  |
| None | 240 | 20 | 35 | 295 | 532 | 137 | 62 | 731 | 772 | 157 | 97 | 1026 |
| ≥ 1 case | 53 | 4 | 14 | 71 | 188 | 49 | 54 | 291 | 241 | 53 | 68 | 362 |
| Unknown | 26 | 2 | 3 | 31 | 46 | 4 | 8 | 58 | 72 | 6 | 11 | 89 |
| Missing | 61 | 7 | 18 | 86 | 167 | 54 | 17 | 238 | 228 | 61 | 35 | 324 |
| Breast cancer PRS mean (SD) |  |  |  |  |  |  |  |  |  |  |  |  |
| BC | -0.33 (0.67) | -0.10 (0.53) | -0.36 (0.61) | -0.31 (0.65) | -0.30 (0.65) | -0.11 (0.64) | -0.26 (0.64) | -0.26 (0.65) | -0.31 (0.65) | -0.11 (0.63) | -0.29 (0.63) | -0.27 (0.65) |
| ER- | -0.21 (0.62) | -0.14 (0.65) | -0.12 (0.57) | -0.19 (0.61) | -0.21 (0.63) | -0.09 (0.62) | -0.21 (0.55) | -0.19 (0.62) | -0.21 (0.63) | -0.10 (0.63) | -0.18 (0.56) | -0.19 (0.62) |
| ER+ | -0.32 (0.72) | -0.08 (0.54) | -0.38 (0.66) | -0.31 (0.70) | -0.29 (0.69) | -0.08 (0.68) | -0.23 (0.69) | -0.24 (0.69) | -0.29 (0.70) | -0.08 (0.66) | -0.28 (0.68) | -0.26 (0.69) |
| Prostate cancer PRS mean (SD) | 11.78 (0.64) | 11.76 (0.69) | 12.09 (0.69) | 11.82 (0.66) | 11.77 (0.69) | 11.62 (0.66) | 12.10 (0.71) | 11.78 (0.70) | 11.77 (0.68) | 11.64 (0.67) | 12.09 (0.70) | 11.79 (0.69) |

^a^ Pathogenic variant classes: “class I” pathogenic variant = loss-of-function pathogenic variants expected to result in unstable or no protein; “class II” pathogenic variant = pathogenic variants likely to yield stable mutant proteins; “class III” have unknown consequence. IQR interquartile range; OCCR = ovarian cancer cluster region; PCCR = prostate cancer cluster region; ER = estrogen receptor; SD = standard deviation.

**Supplementary Table 3**: The 313 SNPs used to construct the breast cancer PRS^6^.^a^

| SNP | Chr | Position | Alleles | | log(OR) / β | | | | Imputation accuracy *r*^2^ |
| --- | --- | --- | --- | --- | --- | --- | --- | --- | --- |
|  |  |  | Reference | Effect | Overall | ER-positive | ER-negative |  | |
| 1_100880328_A_T | 1 | 100880328 | A | T | 0.0373 | 0.0373 | 0.0373 | 1.000 | |
| 1_10566215_A_G | 1 | 10566215 | A | G | -0.0586 | -0.0407 | -0.1109 | 1.000 | |
| 1_110198129_CAAA_C | 1 | 110198129 | CAAA | C | 0.0458 | 0.0458 | 0.0458 | 0.931 | |
| 1_114445880_G_A | 1 | 114445880 | G | A | 0.0621 | 0.0621 | 0.0621 | 0.998 | |
| 1_118141492_A_C | 1 | 118141492 | A | C | 0.0452 | 0.0452 | 0.0452 | 0.997 | |
| 1_120257110_T_C | 1 | 120257110 | T | C | 0.0385 | 0.043 | 0.0226 | 0.996 | |
| 1_121280613_A_G | 1 | 121280613 | A | G | 0.0881 | 0.1052 | 0.0209 | 1.000 | |
| 1_121287994_A_G | 1 | 121287994 | A | G | -0.0673 | -0.0814 | -0.0114 | 0.964 | |
| 1_145604302_C_CT | 1 | 145604302 | C | CT | -0.0399 | -0.0469 | -0.0126 | 0.935 | |
| 1_149906413_T_C | 1 | 149906413 | T | C | 0.0548 | 0.0548 | 0.0548 | 1.000 | |
| 1_155556971_G_A | 1 | 155556971 | G | A | 0.0499 | 0.0499 | 0.0499 | 0.995 | |
| 1_168171052_CA_C | 1 | 168171052 | CA | C | -0.068 | -0.068 | -0.068 | 0.883 | |
| 1_172328767_T_TA | 1 | 172328767 | T | TA | -0.0435 | -0.0435 | -0.0435 | 0.909 | |
| 1_18807339_T_C | 1 | 18807339 | T | C | -0.0564 | -0.0649 | -0.0248 | 0.998 | |
| 1_201437832_C_T | 1 | 201437832 | C | T | 0.0917 | 0.0917 | 0.0917 | 1.000 | |
| 1_202184600_C_T | 1 | 202184600 | C | T | -0.0065 | 0.0133 | -0.0822 | 1.000 | |
| 1_203770448_T_A | 1 | 203770448 | T | A | 0.0498 | 0.0498 | 0.0498 | 0.996 | |
| 1_204502514_T_TTCTGAAACAGGG | 1 | 204502514 | T | TTCTGAAACAGGG | -0.0321 | -0.0024 | -0.1345 | 0.972 | |
| 1_208076291_G_A | 1 | 208076291 | G | A | -0.0366 | -0.0366 | -0.0366 | 0.994 | |
| 1_217053815_T_G | 1 | 217053815 | T | G | 0.0417 | 0.0417 | 0.0417 | 0.897 | |
| 1_217220574_G_A | 1 | 217220574 | G | A | -0.044 | -0.0459 | 0.0029 | 0.995 | |
| 1_220671050_C_T | 1 | 220671050 | C | T | 0.0418 | 0.0418 | 0.0418 | 0.941 | |
| 1_242034263_A_G | 1 | 242034263 | A | G | 0.1428 | 0.1428 | 0.1428 | 1.000 | |
| 1_41380440_C_T | 1 | 41380440 | C | T | 0.0426 | 0.0426 | 0.0426 | 0.978 | |
| 1_41389220_T_C | 1 | 41389220 | T | C | 0.155 | 0.155 | 0.155 | 0.945 | |
| 1_46670206_TC_T | 1 | 46670206 | TC | T | 0.0447 | 0.0595 | 0.0216 | 1.000 | |
| 1_51467096_CT_C | 1 | 51467096 | CT | C | 0.0374 | 0.0374 | 0.0374 | 0.906 | |
| 1_7917076_G_A | 1 | 7917076 | G | A | -0.0409 | -0.0409 | -0.0409 | 0.990 | |
| 1_88156923_G_A | 1 | 88156923 | G | A | 0.0494 | 0.058 | 0.0183 | 1.000 | |
| 1_88428199_C_A | 1 | 88428199 | C | A | -0.0387 | -0.0387 | -0.0387 | 1.000 | |
| 2_10138983_T_C | 2 | 10138983 | T | C | 0.0603 | 0.0603 | 0.0603 | 0.950 | |
| 2_121058254_A_G | 2 | 121058254 | A | G | -0.0334 | -0.0232 | -0.0682 | 0.996 | |
| 2_121089731_T_C | 2 | 121089731 | T | C | -0.0427 | -0.029 | -0.1027 | 0.909 | |
| 2_121159205_G_A | 2 | 121159205 | G | A | -0.044 | -0.044 | -0.044 | 1.000 | |
| 2_121246568_T_C | 2 | 121246568 | T | C | 0.0992 | 0.0992 | 0.0992 | 1.000 | |
| 2_172974566_C_G | 2 | 172974566 | C | G | -0.0473 | -0.0611 | -0.0061 | 1.000 | |
| 2_174212910_A_G | 2 | 174212910 | A | G | 0.0593 | 0.0621 | 0.0175 | 1.000 | |
| 2_192381934_C_T | 2 | 192381934 | C | T | 0.0316 | 0.018 | 0.1012 | 1.000 | |
| 2_19315675_T_A | 2 | 19315675 | T | A | -0.0331 | -0.0229 | -0.057 | 1.000 | |
| 2_202204741_T_C | 2 | 202204741 | T | C | -0.0492 | -0.0492 | -0.0492 | 1.000 | |
| 2_217920769_G_T | 2 | 217920769 | G | T | -0.1318 | -0.1532 | -0.0589 | 1.000 | |
| 2_217955896_GA_G | 2 | 217955896 | GA | G | -0.2016 | -0.2362 | -0.0558 | 0.985 | |
| 2_218292158_C_G | 2 | 218292158 | C | G | -0.0757 | -0.0757 | -0.0757 | 0.976 | |
| 2_218714845_G_A | 2 | 218714845 | G | A | -0.0431 | -0.0463 | -0.0184 | 1.000 | |
| 2_241388857_C_A | 2 | 241388857 | C | A | -0.1232 | -0.1232 | -0.1232 | 1.000 | |
| 2_25129473_A_G | 2 | 25129473 | A | G | -0.0427 | -0.0427 | -0.0427 | 1.000 | |
| 2_29179452_G_C | 2 | 29179452 | G | C | -0.0066 | 0.0207 | -0.1006 | 1.000 | |
| 2_29615233_T_C | 2 | 29615233 | T | C | -0.0427 | -0.0427 | -0.0427 | 0.983 | |
| 2_39699510_C_CT | 2 | 39699510 | C | CT | -0.0402 | -0.0402 | -0.0402 | 0.886 | |
| 2_70172587_G_A | 2 | 70172587 | G | A | -0.0412 | -0.0412 | -0.0412 | 0.962 | |
| 2_88358825_G_C | 2 | 88358825 | G | C | 0.0473 | 0.0473 | 0.0473 | 0.950 | |
| 3_141112859_CTT_C | 3 | 141112859 | CTT | C | 0.0551 | 0.0551 | 0.0551 | 0.996 | |
| 3_172285237_G_A | 3 | 172285237 | G | A | 0.0422 | 0.0501 | -0.0133 | 0.997 | |
| 3_189774456_C_T | 3 | 189774456 | C | T | -0.0478 | -0.0478 | -0.0478 | 0.995 | |
| 3_27353716_C_A | 3 | 27353716 | C | A | 0.0748 | 0.0822 | 0.031 | 1.000 | |
| 3_27388664_C_G | 3 | 27388664 | C | G | 0.0502 | 0.0502 | 0.0502 | 1.000 | |
| 3_29294845_C_T | 3 | 29294845 | C | T | -0.1281 | -0.1221 | -0.2988 | 0.922 | |
| 3_30684907_C_T | 3 | 30684907 | C | T | 0.0592 | 0.0657 | 0.017 | 1.000 | |
| 3_46888198_T_C | 3 | 46888198 | T | C | -0.0806 | -0.0806 | -0.0806 | 0.994 | |
| 3_4742251_A_G | 3 | 4742251 | A | G | 0.0616 | 0.0616 | 0.0616 | 1.000 | |
| 3_49709912_C_CT | 3 | 49709912 | C | CT | -0.0367 | -0.0355 | -0.0721 | 0.941 | |
| 3_55970777_A_AT | 3 | 55970777 | A | AT | -0.1195 | -0.1195 | -0.1195 | 0.957 | |
| 3_59373745_C_T | 3 | 59373745 | C | T | -0.0394 | -0.0394 | -0.0394 | 0.995 | |
| 3_63887449_T_TTG | 3 | 63887449 | T | TTG | 0.0648 | 0.0648 | 0.0648 | 0.989 | |
| 3_71620370_T_G | 3 | 71620370 | T | G | -0.0374 | -0.0374 | -0.0374 | 0.988 | |
| 3_87037543_A_G | 3 | 87037543 | A | G | -0.0723 | -0.0723 | -0.0723 | 0.946 | |
| 3_99403877_G_A | 3 | 99403877 | G | A | -0.0376 | -0.0376 | -0.0376 | 0.996 | |
| 4_106069013_G_T | 4 | 106069013 | G | T | 0.0471 | 0.0594 | 0.0097 | 1.000 | |
| 4_126752992_A_AAT | 4 | 126752992 | A | AAT | -0.0377 | -0.0377 | -0.0377 | 0.957 | |
| 4_143467195_C_T | 4 | 143467195 | C | T | -0.0569 | -0.0569 | -0.0569 | 0.998 | |
| 4_151218296_CATATTT_C | 4 | 151218296 | CATATTT | C | 0.0388 | 0.0388 | 0.0388 | 0.992 | |
| 4_175842495_G_A | 4 | 175842495 | G | A | -0.0898 | -0.1162 | 0.0199 | 1.000 | |
| 4_175847436_C_A | 4 | 175847436 | C | A | 0.0348 | 0.0537 | -0.0099 | 1.000 | |
| 4_187503758_A_T | 4 | 187503758 | A | T | 0.0357 | 0.0357 | 0.0357 | 0.997 | |
| 4_38784633_G_T | 4 | 38784633 | G | T | 0.0489 | 0.0489 | 0.0489 | 0.999 | |
| 4_84370124_TAA_TA | 4 | 84370124 | TAA | TA | -0.0464 | -0.0464 | -0.0464 | 0.944 | |
| 4_89240476_G_A | 4 | 89240476 | G | A | 0.0352 | 0.0352 | 0.0352 | 0.977 | |
| 4_92594859_TTCTTTC_T | 4 | 92594859 | TTCTTTC | T | -0.0407 | -0.0407 | -0.0407 | 0.932 | |
| 5_104300273_G_T | 5 | 104300273 | G | T | -0.0487 | -0.0487 | -0.0487 | 0.994 | |
| 5_122478676_C_A | 5 | 122478676 | C | A | -0.0386 | -0.0386 | -0.0386 | 0.999 | |
| 5_122705244_C_T | 5 | 122705244 | C | T | 0.0944 | 0.0944 | 0.0944 | 0.993 | |
| 5_1279790_C_T | 5 | 1279790 | C | T | 0.0617 | 0.0325 | 0.1502 | 1.000 | |
| 5_1296255_A_AG | 5 | 1296255 | A | AG | -0.0549 | -0.0417 | -0.1056 | 1.000 | |
| 5_131640536_A_G | 5 | 131640536 | A | G | 0.0392 | 0.0467 | 0.0099 | 0.977 | |
| 5_132407058_C_T | 5 | 132407058 | C | T | -0.0388 | -0.0561 | -0.0214 | 0.998 | |
| 5_1353077_T_C | 5 | 1353077 | T | C | 0.1552 | 0.1552 | 0.1552 | 1.000 | |
| 5_158244083_C_T | 5 | 158244083 | C | T | -0.0677 | -0.0677 | -0.0677 | 1.000 | |
| 5_16231194_G_C | 5 | 16231194 | G | C | -0.0426 | -0.0426 | -0.0426 | 1.000 | |
| 5_169591460_T_C | 5 | 169591460 | T | C | 0.0412 | 0.0501 | 0.0182 | 0.997 | |
| 5_173358154_G_A | 5 | 173358154 | G | A | 0.0365 | 0.0365 | 0.0365 | 0.986 | |
| 5_176134882_T_C | 5 | 176134882 | T | C | 0.0363 | 0.0363 | 0.0363 | 0.994 | |
| 5_2777029_G_A | 5 | 2777029 | G | A | 0.0391 | 0.0391 | 0.0391 | 0.983 | |
| 5_32579616_TCA_T | 5 | 32579616 | TCA | T | 0.0363 | 0.0363 | 0.0363 | 1.000 | |
| 5_345109_T_C | 5 | 345109 | T | C | 0.084 | 0.084 | 0.084 | 0.974 | |
| 5_44508264_G_GT | 5 | 44508264 | G | GT | -0.1177 | -0.1177 | -0.1177 | 0.973 | |
| 5_44619502_A_G | 5 | 44619502 | A | G | -0.1101 | -0.1101 | -0.1101 | 0.994 | |
| 5_44649944_C_T | 5 | 44649944 | C | T | 0.0492 | 0.0713 | -0.0261 | 1.000 | |
| 5_44706498_A_G | 5 | 44706498 | A | G | 0.0497 | 0.0648 | -0.0256 | 1.000 | |
| 5_44853593_G_C | 5 | 44853593 | G | C | -0.0336 | -0.0222 | -0.0778 | 1.000 | |
| 5_52679539_C_CA | 5 | 52679539 | C | CA | 0.0571 | 0.0571 | 0.0571 | 0.969 | |
| 5_55662540_C_CT | 5 | 55662540 | C | CT | -0.0458 | -0.0458 | -0.0458 | 0.968 | |
| 5_55965167_C_T | 5 | 55965167 | C | T | 0.0394 | 0.0394 | 0.0394 | 1.000 | |
| 5_56023083_T_G | 5 | 56023083 | T | G | 0.1366 | 0.1612 | 0.0686 | 1.000 | |
| 5_56042972_C_T | 5 | 56042972 | C | T | 0.0865 | 0.1082 | 0.0058 | 0.997 | |
| 5_56045081_T_C | 5 | 56045081 | T | C | -0.0564 | -0.0643 | -0.0168 | 1.000 | |
| 5_58241712_C_T | 5 | 58241712 | C | T | -0.0434 | -0.0434 | -0.0434 | 0.940 | |
| 5_71965007_G_A | 5 | 71965007 | G | A | -0.041 | -0.041 | -0.041 | 0.947 | |
| 5_73234583_T_C | 5 | 73234583 | T | C | -0.0363 | -0.0494 | -0.0101 | 0.976 | |
| 5_77155397_GT_G | 5 | 77155397 | GT | G | -0.0408 | -0.0408 | -0.0408 | 0.991 | |
| 5_79180995_G_GA | 5 | 79180995 | G | GA | 0.0328 | 0.0248 | 0.0804 | 0.993 | |
| 5_81512947_TA_T | 5 | 81512947 | TA | T | -0.0598 | -0.0731 | -0.0342 | 1.000 | |
| 5_90789470_G_A | 5 | 90789470 | G | A | -0.0564 | -0.0714 | -0.0031 | 1.000 | |
| 6_130341728_C_CT | 6 | 130341728 | C | CT | 0.0472 | 0.0472 | 0.0472 | 0.983 | |
| 6_13713366_G_C | 6 | 13713366 | G | C | -0.0553 | -0.0623 | -0.0152 | 1.000 | |
| 6_149595505_T_C | 6 | 149595505 | T | C | -0.0476 | -0.0476 | -0.0476 | 0.996 | |
| 6_151949806_A_C | 6 | 151949806 | A | C | 0.0703 | 0.0541 | 0.1103 | 1.000 | |
| 6_151955914_A_G | 6 | 151955914 | A | G | 0.1449 | 0.115 | 0.224 | 1.000 | |
| 6_152022664_CAAAAAAA_C | 6 | 152022664 | CAAAAAAA | C | 0.0137 | 0.0137 | 0.0137 | 0.914 | |
| 6_152023191_G_A | 6 | 152023191 | G | A | 0.0626 | 0.0509 | 0.1008 | 1.000 | |
| 6_152055978_A_T | 6 | 152055978 | A | T | 0.074 | 0.074 | 0.074 | 0.990 | |
| 6_152432902_C_T | 6 | 152432902 | C | T | 0.0649 | 0.0527 | 0.0965 | 1.000 | |
| 6_16399557_C_T | 6 | 16399557 | C | T | -0.0373 | -0.0373 | -0.0373 | 1.000 | |
| 6_169006947_C_G | 6 | 169006947 | C | G | -0.0308 | -0.0252 | -0.0628 | 0.932 | |
| 6_170332621_T_C | 6 | 170332621 | T | C | 0.0373 | 0.0373 | 0.0373 | 0.991 | |
| 6_18783140_G_A | 6 | 18783140 | G | A | 0.0326 | 0.0478 | 0.0033 | 0.998 | |
| 6_20537845_CA_C | 6 | 20537845 | CA | C | -0.0391 | -0.0391 | -0.0391 | 0.903 | |
| 6_21923810_T_C | 6 | 21923810 | T | C | -0.0321 | -0.0438 | -0.0032 | 0.999 | |
| 6_27425644_G_C | 6 | 27425644 | G | C | -0.0737 | -0.0737 | -0.0737 | 0.995 | |
| 6_43227141_G_A | 6 | 43227141 | G | A | -0.064 | -0.064 | -0.064 | 0.995 | |
| 6_82263549_AAT_A | 6 | 82263549 | AAT | A | 0.0477 | 0.0477 | 0.0477 | 0.922 | |
| 6_85912194_CAA_C | 6 | 85912194 | CAA | C | 0.0762 | 0.0762 | 0.0762 | 0.956 | |
| 6_87803819_T_C | 6 | 87803819 | T | C | 0.0383 | 0.0318 | 0.0678 | 0.989 | |
| 7_101552440_G_A | 7 | 101552440 | G | A | -0.0568 | -0.0568 | -0.0568 | 0.994 | |
| 7_102481842_T_C | 7 | 102481842 | T | C | 0.0418 | 0.0418 | 0.0418 | 0.997 | |
| 7_130656911_C_T | 7 | 130656911 | C | T | -0.0476 | -0.0476 | -0.0476 | 0.951 | |
| 7_130674481_G_A | 7 | 130674481 | G | A | 0.0416 | 0.0416 | 0.0416 | 1.000 | |
| 7_139943702_CT_C | 7 | 139943702 | CT | C | 0.0582 | 0.0666 | 0.0057 | 0.937 | |
| 7_144048902_G_T | 7 | 144048902 | G | T | -0.0563 | -0.0592 | -0.0148 | 0.906 | |
| 7_21940960_A_G | 7 | 21940960 | A | G | -0.0467 | -0.0467 | -0.0467 | 0.989 | |
| 7_25569548_C_T | 7 | 25569548 | C | T | -0.0486 | -0.0486 | -0.0486 | 0.947 | |
| 7_28869017_G_A | 7 | 28869017 | G | A | -0.0572 | -0.0572 | -0.0572 | 0.992 | |
| 7_55192256_A_C | 7 | 55192256 | A | C | -0.0349 | -0.0349 | -0.0349 | 1.000 | |
| 7_91459189_A_ATT | 7 | 91459189 | A | ATT | 0.0452 | 0.0452 | 0.0452 | 0.932 | |
| 7_94113799_T_C | 7 | 94113799 | T | C | 0.0449 | 0.0449 | 0.0449 | 0.995 | |
| 7_98005235_G_A | 7 | 98005235 | G | A | -0.0467 | -0.0467 | -0.0467 | 1.000 | |
| 7_99948655_T_G | 7 | 99948655 | T | G | 0.042 | 0.042 | 0.042 | 0.982 | |
| 8_102483100_T_C | 8 | 102483100 | T | C | 0.0593 | 0.0736 | 0.0137 | 0.942 | |
| 8_106358620_A_T | 8 | 106358620 | A | T | -0.0745 | -0.0895 | -0.01 | 0.982 | |
| 8_117209548_A_G | 8 | 117209548 | A | G | -0.0417 | -0.0417 | -0.0417 | 1.000 | |
| 8_120862186_A_G | 8 | 120862186 | A | G | 0.0527 | 0.0527 | 0.0527 | 0.974 | |
| 8_124563705_T_C | 8 | 124563705 | T | C | 0.0477 | 0.0477 | 0.0477 | 0.988 | |
| 8_124571581_G_A | 8 | 124571581 | G | A | 0.034 | 0.034 | 0.034 | 0.992 | |
| 8_124739913_T_G | 8 | 124739913 | T | G | 0.0466 | 0.0466 | 0.0466 | 0.978 | |
| 8_128213561_C_CA | 8 | 128213561 | C | CA | -0.043 | -0.043 | -0.043 | 1.000 | |
| 8_128370949_C_G | 8 | 128370949 | C | G | 0.0642 | 0.082 | 0.0076 | 0.998 | |
| 8_128372172_A_G | 8 | 128372172 | A | G | 0.0597 | 0.0597 | 0.0597 | 1.000 | |
| 8_129199566_G_A | 8 | 129199566 | G | A | 0.0615 | 0.0615 | 0.0615 | 1.000 | |
| 8_143669254_A_G | 8 | 143669254 | A | G | -0.0346 | -0.0346 | -0.0346 | 0.955 | |
| 8_170692_T_C | 8 | 170692 | T | C | 0.0477 | 0.0348 | 0.104 | 0.919 | |
| 8_17787610_CT_C | 8 | 17787610 | CT | C | -0.0377 | -0.0377 | -0.0377 | 0.925 | |
| 8_23447496_A_G | 8 | 23447496 | A | G | -0.0389 | -0.0389 | -0.0389 | 1.000 | |
| 8_23663653_C_A | 8 | 23663653 | C | A | 0.0335 | 0.0451 | 0.0059 | 0.999 | |
| 8_29509616_A_C | 8 | 29509616 | A | C | -0.0601 | -0.0601 | -0.0601 | 1.000 | |
| 8_36858483_A_G | 8 | 36858483 | A | G | -0.076 | -0.076 | -0.076 | 1.000 | |
| 8_76230943_A_G | 8 | 76230943 | A | G | 0.0755 | 0.0755 | 0.0755 | 1.000 | |
| 8_76333056_C_T | 8 | 76333056 | C | T | 0.1129 | 0.1129 | 0.1129 | 1.000 | |
| 8_76378165_G_T | 8 | 76378165 | G | T | -0.0391 | -0.0391 | -0.0391 | 1.000 | |
| 9_110303808_TAA_T | 9 | 110303808 | TAA | T | 0.0797 | 0.1007 | 0.013 | 0.993 | |
| 9_110837073_A_G | 9 | 110837073 | A | G | 0.1158 | 0.1315 | 0.0289 | 1.000 | |
| 9_110837176_C_T | 9 | 110837176 | C | T | 0.0653 | 0.0809 | -0.0037 | 1.000 | |
| 9_110849525_G_T | 9 | 110849525 | G | T | 0.0153 | 0.0153 | 0.0153 | 1.000 | |
| 9_110885479_C_T | 9 | 110885479 | C | T | 0.0877 | 0.111 | 0.0019 | 1.000 | |
| 9_119313486_A_G | 9 | 119313486 | A | G | -0.0462 | -0.0462 | -0.0462 | 0.987 | |
| 9_129424719_A_G | 9 | 129424719 | A | G | -0.0382 | -0.0382 | -0.0382 | 0.950 | |
| 9_136146597_C_T | 9 | 136146597 | C | T | 0.04 | 0.04 | 0.04 | 0.999 | |
| 9_21964882_CAAAA_C | 9 | 21964882 | CAAAA | C | 0.055 | 0.055 | 0.055 | 0.973 | |
| 9_22041998_C_G | 9 | 22041998 | C | G | 0.0289 | 0.0168 | 0.0906 | 1.000 | |
| 9_36928288_T_C | 9 | 36928288 | T | C | 0.0249 | 0.0249 | 0.0249 | 0.992 | |
| 9_6880263_A_G | 9 | 6880263 | A | G | 0.0348 | 0.0499 | -0.0078 | 1.000 | |
| 9_87782211_T_C | 9 | 87782211 | T | C | 0.0361 | 0.0361 | 0.0361 | 1.000 | |
| 9_98362587_T_C | 9 | 98362587 | T | C | 0.0576 | 0.0576 | 0.0576 | 0.991 | |
| 10_114777670_C_T | 10 | 114777670 | C | T | 0.0472 | 0.0472 | 0.0472 | 1.000 | |
| 10_115128491_T_C | 10 | 115128491 | T | C | -0.0592 | -0.0592 | -0.0592 | 1.000 | |
| 10_123095209_G_A | 10 | 123095209 | G | A | -0.0538 | -0.0702 | 0.0048 | 0.995 | |
| 10_123340107_A_G | 10 | 123340107 | A | G | 0.1508 | 0.1837 | 0.0053 | 0.998 | |
| 10_123340431_GC_G | 10 | 123340431 | GC | G | -0.2408 | -0.2913 | -0.0326 | 0.999 | |
| 10_123349324_A_T | 10 | 123349324 | A | T | -0.2609 | -0.327 | -0.0137 | 0.966 | |
| 10_13892298_G_A | 10 | 13892298 | G | A | 0.0371 | 0.0371 | 0.0371 | 1.000 | |
| 10_22032942_A_G | 10 | 22032942 | A | G | -0.058 | -0.0719 | 0.0344 | 1.000 | |
| 10_22477776_ACC_A | 10 | 22477776 | ACC | A | 0.1687 | 0.1687 | 0.1687 | 0.982 | |
| 10_22861490_A_C | 10 | 22861490 | A | C | 0.0875 | 0.096 | 0.0201 | 0.975 | |
| 10_38523626_C_A | 10 | 38523626 | C | A | 0.0404 | 0.0404 | 0.0404 | 0.957 | |
| 10_5794652_A_G | 10 | 5794652 | A | G | 0.047 | 0.047 | 0.047 | 1.000 | |
| 10_64299890_A_G | 10 | 64299890 | A | G | -0.1345 | -0.1428 | -0.103 | 0.981 | |
| 10_64819996_G_T | 10 | 64819996 | G | T | 0.0472 | 0.0472 | 0.0472 | 1.000 | |
| 10_71335574_C_T | 10 | 71335574 | C | T | -0.0404 | -0.0404 | -0.0404 | 0.956 | |
| 10_80851257_G_T | 10 | 80851257 | G | T | -0.0805 | -0.0898 | -0.0443 | 1.000 | |
| 10_80886726_A_G | 10 | 80886726 | A | G | 0.0762 | 0.0762 | 0.0762 | 1.000 | |
| 10_95292187_CAA_C | 10 | 95292187 | CAA | C | -0.0512 | -0.0512 | -0.0512 | 0.929 | |
| 11_103614438_T_G | 11 | 103614438 | T | G | 0.0147 | 0.0029 | 0.0676 | 0.992 | |
| 11_108267402_C_CA | 11 | 108267402 | C | CA | -0.0022 | 0.0141 | -0.0629 | 0.999 | |
| 11_111696440_T_C | 11 | 111696440 | T | C | -0.0396 | -0.0396 | -0.0396 | 0.997 | |
| 11_116727936_A_T | 11 | 116727936 | A | T | -0.0423 | -0.0423 | -0.0423 | 0.997 | |
| 11_122966626_A_G | 11 | 122966626 | A | G | -0.0383 | -0.0383 | -0.0383 | 0.998 | |
| 11_129243417_T_G | 11 | 129243417 | T | G | -0.0543 | -0.0543 | -0.0543 | 0.990 | |
| 11_129461016_A_G | 11 | 129461016 | A | G | 0.0453 | 0.0453 | 0.0453 | 1.000 | |
| 11_18664241_T_G | 11 | 18664241 | T | G | 0.0461 | 0.0461 | 0.0461 | 0.948 | |
| 11_1895708_C_A | 11 | 1895708 | C | A | -0.0762 | -0.0762 | -0.0762 | 1.000 | |
| 11_42844441_C_T | 11 | 42844441 | C | T | -0.0336 | -0.0336 | -0.0336 | 1.000 | |
| 11_433617_T_C | 11 | 433617 | T | C | -0.0437 | -0.0437 | -0.0437 | 0.984 | |
| 11_44368892_G_A | 11 | 44368892 | G | A | 0.0374 | 0.0374 | 0.0374 | 0.993 | |
| 11_46318032_C_G | 11 | 46318032 | C | G | -0.0748 | -0.0748 | -0.0748 | 0.904 | |
| 11_65553492_C_A | 11 | 65553492 | C | A | 0.0425 | 0.0425 | 0.0425 | 0.997 | |
| 11_65572431_G_A | 11 | 65572431 | G | A | -0.0347 | -0.0448 | -0.0067 | 0.999 | |
| 11_69328130_A_T | 11 | 69328130 | A | T | -0.0423 | -0.0538 | 0.0143 | 0.953 | |
| 11_69330983_G_A | 11 | 69330983 | G | A | 0.1022 | 0.124 | 0.0174 | 1.000 | |
| 11_69331418_C_T | 11 | 69331418 | C | T | 0.1782 | 0.2018 | 0.0066 | 0.993 | |
| 11_803017_A_G | 11 | 803017 | A | G | 0.0457 | 0.0457 | 0.0457 | 0.994 | |
| 12_103097887_C_T | 12 | 103097887 | C | T | 0.0546 | 0.0611 | 0.0149 | 0.988 | |
| 12_111600134_G_T | 12 | 111600134 | G | T | -0.0442 | -0.0442 | -0.0442 | 1.000 | |
| 12_115108136_T_C | 12 | 115108136 | T | C | 0.0465 | 0.0465 | 0.0465 | 1.000 | |
| 12_115796577_A_G | 12 | 115796577 | A | G | -0.0428 | -0.0643 | -0.0148 | 0.998 | |
| 12_115835836_T_C | 12 | 115835836 | T | C | -0.0813 | -0.0977 | -0.0153 | 1.000 | |
| 12_120832146_C_T | 12 | 120832146 | C | T | 0.0516 | 0.0516 | 0.0516 | 1.000 | |
| 12_14413931_G_C | 12 | 14413931 | G | C | 0.0484 | 0.0484 | 0.0484 | 1.000 | |
| 12_28149568_C_T | 12 | 28149568 | C | T | -0.062 | -0.062 | -0.062 | 1.000 | |
| 12_28174817_C_T | 12 | 28174817 | C | T | -0.0856 | -0.0856 | -0.0856 | 1.000 | |
| 12_28347382_C_T | 12 | 28347382 | C | T | -0.0521 | -0.0521 | -0.0521 | 0.983 | |
| 12_29140260_G_A | 12 | 29140260 | G | A | 0.0647 | 0.0647 | 0.0647 | 0.997 | |
| 12_293626_A_G | 12 | 293626 | A | G | 0.0401 | 0.0401 | 0.0401 | 0.994 | |
| 12_57146069_T_G | 12 | 57146069 | T | G | -0.0579 | -0.0579 | -0.0579 | 1.000 | |
| 12_70798355_A_T | 12 | 70798355 | A | T | 0.0469 | 0.0469 | 0.0469 | 0.995 | |
| 12_83064195_G_GA | 12 | 83064195 | G | GA | 0.0671 | 0.0671 | 0.0671 | 0.973 | |
| 12_85004551_C_T | 12 | 85004551 | C | T | 0.0348 | 0.0348 | 0.0348 | 0.985 | |
| 12_96027759_A_G | 12 | 96027759 | A | G | -0.0867 | -0.0867 | -0.0867 | 1.000 | |
| 13_32839990_G_A | 13 | 32839990 | G | A | 0.0424 | 0.0424 | 0.0424 | 1.000 | |
| 13_32972626_A_T | 13 | 32972626 | A | T | 0.2687 | 0.2308 | 0.4284 | 1.000 | |
| 13_43501356_A_G | 13 | 43501356 | A | G | 0.0517 | 0.0458 | 0.0975 | 0.968 | |
| 13_73806982_T_C | 13 | 73806982 | T | C | 0.0345 | 0.0251 | 0.0653 | 1.000 | |
| 13_73960952_A_G | 13 | 73960952 | A | G | 0.0399 | 0.0368 | 0.073 | 1.000 | |
| 14_105213978_T_G | 14 | 105213978 | T | G | 0.0399 | 0.0399 | 0.0399 | 0.983 | |
| 14_37128564_C_A | 14 | 37128564 | C | A | -0.0733 | -0.085 | -0.0339 | 1.000 | |
| 14_37228504_C_T | 14 | 37228504 | C | T | 0.039 | 0.039 | 0.039 | 0.997 | |
| 14_68660428_T_C | 14 | 68660428 | T | C | -0.0474 | -0.0612 | 0.0245 | 1.000 | |
| 14_68979835_T_C | 14 | 68979835 | T | C | -0.0911 | -0.0911 | -0.0911 | 1.000 | |
| 14_91751788_TC_T | 14 | 91751788 | TC | T | 0.038 | 0.0447 | 0.0091 | 1.000 | |
| 14_91841069_A_G | 14 | 91841069 | A | G | 0.0513 | 0.0513 | 0.0513 | 1.000 | |
| 14_93070286_C_T | 14 | 93070286 | C | T | -0.0577 | -0.0577 | -0.0577 | 1.000 | |
| 15_100905819_A_C | 15 | 100905819 | A | C | -0.0608 | -0.0608 | -0.0608 | 0.949 | |
| 15_46680811_C_A | 15 | 46680811 | C | A | -0.1973 | -0.1973 | -0.1973 | 0.948 | |
| 15_50694306_A_G | 15 | 50694306 | A | G | -0.0417 | -0.0417 | -0.0417 | 0.956 | |
| 15_66630569_G_A | 15 | 66630569 | G | A | -0.0369 | -0.0369 | -0.0369 | 0.976 | |
| 15_67457698_A_G | 15 | 67457698 | A | G | 0.0782 | 0.099 | 0.0141 | 1.000 | |
| 15_75750383_T_C | 15 | 75750383 | T | C | -0.0413 | -0.0413 | -0.0413 | 0.971 | |
| 15_91512267_G_T | 15 | 91512267 | G | T | -0.0589 | -0.0589 | -0.0589 | 1.000 | |
| 16_10706580_G_A | 16 | 10706580 | G | A | -0.074 | -0.074 | -0.074 | 0.966 | |
| 16_23007047_G_T | 16 | 23007047 | G | T | 0.1218 | 0.1218 | 0.1218 | 0.887 | |
| 16_4008542_CAAAAA_C | 16 | 4008542 | CAAAAA | C | -0.0329 | -0.0184 | -0.0892 | 0.897 | |
| 16_4106788_C_A | 16 | 4106788 | C | A | -0.03 | -0.0182 | -0.0782 | 0.969 | |
| 16_52538825_C_A | 16 | 52538825 | C | A | 0.1147 | 0.1147 | 0.1147 | 0.999 | |
| 16_52599188_C_T | 16 | 52599188 | C | T | 0.107 | 0.107 | 0.107 | 1.000 | |
| 16_53809123_C_T | 16 | 53809123 | C | T | -0.0704 | -0.0651 | -0.0957 | 1.000 | |
| 16_53861139_C_T | 16 | 53861139 | C | T | -0.0338 | -0.0167 | -0.0782 | 1.000 | |
| 16_53861592_G_A | 16 | 53861592 | G | A | -0.0337 | -0.0337 | -0.0337 | 1.000 | |
| 16_54682064_G_A | 16 | 54682064 | G | A | 0.0477 | 0.0477 | 0.0477 | 1.000 | |
| 16_6963972_C_G | 16 | 6963972 | C | G | 0.0354 | 0.0303 | 0.0811 | 0.962 | |
| 16_80648296_A_G | 16 | 80648296 | A | G | 0.0839 | 0.089 | 0.0467 | 0.999 | |
| 16_85145977_T_C | 16 | 85145977 | T | C | -0.0211 | -0.0044 | -0.0714 | 0.955 | |
| 16_87086492_T_C | 16 | 87086492 | T | C | -0.0469 | -0.0469 | -0.0469 | 0.997 | |
| 17_29168077_G_T | 17 | 29168077 | G | T | -0.0568 | -0.0568 | -0.0568 | 0.988 | |
| 17_39251123_T_C | 17 | 39251123 | T | C | 0.0799 | 0.0631 | 0.1431 | 0.891 | |
| 17_40127060_T_C | 17 | 40127060 | T | C | 0.0174 | -0.0161 | 0.1511 | 1.000 | |
| 17_40485239_G_T | 17 | 40485239 | G | T | -0.0571 | -0.0416 | -0.1142 | 0.937 | |
| 17_40744470_G_A | 17 | 40744470 | G | A | 0.2017 | 0.2017 | 0.2017 | 0.958 | |
| 17_43212339_C_CT | 17 | 43212339 | C | CT | 0.0438 | 0.0438 | 0.0438 | 0.976 | |
| 17_44283858_G_A | 17 | 44283858 | G | A | -0.054 | -0.054 | -0.054 | 0.958 | |
| 17_53209774_A_C | 17 | 53209774 | A | C | -0.0793 | -0.0933 | -0.0365 | 0.999 | |
| 17_77781725_A_G | 17 | 77781725 | A | G | -0.0401 | -0.0401 | -0.0401 | 1.000 | |
| 18_11696613_C_T | 18 | 11696613 | C | T | -0.0381 | -0.0281 | -0.094 | 1.000 | |
| 18_20634253_C_T | 18 | 20634253 | C | T | -0.0415 | -0.0415 | -0.0415 | 0.983 | |
| 18_24125857_T_C | 18 | 24125857 | T | C | 0.0346 | 0.0346 | 0.0346 | 1.000 | |
| 18_24337424_C_G | 18 | 24337424 | C | G | 0.0455 | 0.0455 | 0.0455 | 1.000 | |
| 18_24518050_AT_A | 18 | 24518050 | AT | A | -0.0599 | -0.083 | 0.006 | 1.000 | |
| 18_25407513_C_G | 18 | 25407513 | C | G | 0.0399 | 0.0307 | 0.0648 | 0.982 | |
| 18_29981526_G_A | 18 | 29981526 | G | A | -0.1058 | -0.1058 | -0.1058 | 0.999 | |
| 18_42411803_G_C | 18 | 42411803 | G | C | -0.0877 | -0.1037 | -0.0189 | 0.997 | |
| 18_42888797_T_C | 18 | 42888797 | T | C | -0.0542 | -0.0542 | -0.0542 | 1.000 | |
| 19_13249921_G_T | 19 | 13249921 | G | T | 0.0956 | 0.0956 | 0.0956 | 0.978 | |
| 19_17393925_C_A | 19 | 17393925 | C | A | 0.0378 | 0.0036 | 0.1692 | 1.000 | |
| 19_18569492_C_T | 19 | 18569492 | C | T | -0.0719 | -0.0719 | -0.0719 | 1.000 | |
| 19_19517054_C_CGGGCG | 19 | 19517054 | C | CGGGCG | 0.0437 | 0.0437 | 0.0437 | 0.999 | |
| 19_44283031_T_C | 19 | 44283031 | T | C | 0.0619 | 0.0619 | 0.0619 | 1.000 | |
| 19_46166073_T_C | 19 | 46166073 | T | C | -0.036 | -0.0447 | -0.0117 | 0.950 | |
| 19_55816678_C_T | 19 | 55816678 | C | T | -0.0359 | -0.0359 | -0.0359 | 0.993 | |
| 20_11379842_T_C | 20 | 11379842 | T | C | 0.0844 | 0.0844 | 0.0844 | 0.954 | |
| 20_41613706_C_G | 20 | 41613706 | C | G | 0.0315 | 0.0266 | 0.0784 | 0.939 | |
| 20_52296849_G_A | 20 | 52296849 | G | A | 0.044 | 0.0539 | 0.0144 | 0.949 | |
| 20_5948227_G_A | 20 | 5948227 | G | A | 0.076 | 0.076 | 0.076 | 1.000 | |
| 21_16364756_T_G | 21 | 16364756 | T | G | 0.0646 | 0.0646 | 0.0646 | 1.000 | |
| 21_16566350_A_G | 21 | 16566350 | A | G | 0.0595 | 0.0678 | 0.0172 | 1.000 | |
| 21_16574455_C_A | 21 | 16574455 | C | A | -0.0707 | -0.0808 | -0.0329 | 0.995 | |
| 21_47762932_G_A | 21 | 47762932 | G | A | 0.0946 | 0.0946 | 0.0946 | 0.979 | |
| 22_19766137_C_T | 22 | 19766137 | C | T | -0.0367 | -0.0367 | -0.0367 | 0.983 | |
| 22_29121087_A_G | 22 | 29121087 | A | G | 0.1839 | 0.2812 | -0.1566 | 1.000 | |
| 22_29135543_G_A | 22 | 29135543 | G | A | 0.0654 | 0.0654 | 0.0654 | 0.998 | |
| 22_29203724_C_T | 22 | 29203724 | C | T | 0.1405 | 0.1793 | 0.0191 | 0.999 | |
| 22_29551872_A_G | 22 | 29551872 | A | G | -0.1716 | -0.1716 | -0.1716 | 0.864 | |
| 22_38583315_AAAAG_AAAAGAAAG | 22 | 38583315 | AAAAG | AAAAGAAAG | -0.0471 | -0.0608 | 0.0079 | 0.960 | |
| 22_39343916_T_A | 22 | 39343916 | T | A | 0.0407 | 0.0407 | 0.0407 | 1.000 | |
| 22_40904707_CT_C | 22 | 40904707 | CT | C | 0.1148 | 0.1148 | 0.1148 | 0.974 | |
| 22_43433100_C_T | 22 | 43433100 | C | T | -0.06 | -0.06 | -0.06 | 0.992 | |
| 22_45319953_G_A | 22 | 45319953 | G | A | -0.0134 | -0.006 | -0.0611 | 0.997 | |
| 22_46283297_G_A | 22 | 46283297 | G | A | 0.0736 | 0.0736 | 0.0736 | 0.965 | |

^a^ The same set of 313 SNPs was used to construct the PRS_ER-_ and PRS_ER+_. The ER-specific PRS used different SNP weights (log-ORs for ER-specific breast cancer) if they had a statistically significant different effect on ER-subtype from a population-based breast cancer case-only analysis. SNP = single nucleotide polymorphism; chr = chromosome; position = human genome build 37 position; OR = per (effect) allele odds ratio association with breast cancer risk estimated from the Breast Cancer Association Consortium (BCAC) population-based data.

**Supplementary Table 4**: The 147 SNPs used to construct the prostate cancer PRS^7^.^a^

| SNP | Chr | Position | Effect allele | log(OR) / β | Imputation accuracy *r*^2^ |
| --- | --- | --- | --- | --- | --- |
| rs56391074 | 1 | 88210715 | AT | 0.047 | 0.993 |
| rs34579442 | 1 | 153899900 | C | 0.066 | 0.923 |
| rs62106670 | 2 | 8597123 | T | 0.052 | 0.899 |
| rs74702681 | 2 | 66652885 | T | 0.159 | 0.971 |
| rs11691517 | 2 | 111893096 | T | 0.064 | 1.000 |
| rs34925593 | 2 | 174234547 | C | 0.047 | 0.998 |
| rs59308963 | 2 | 202123479 | T | 0.051 | 0.996 |
| rs1283104 | 3 | 106962521 | G | 0.047 | 0.996 |
| rs182314334 | 3 | 152004202 | T | 0.089 | 0.996 |
| rs142436749 | 3 | 169093100 | G | 0.221 | 0.910 |
| rs10793821 | 5 | 133836209 | T | 0.053 | 0.993 |
| rs76551843 | 5 | 169172133 | A | 0.271 | 1.000 |
| rs4976790 | 5 | 177968915 | T | 0.074 | 1.000 |
| rs12665339 | 6 | 30601232 | G | 0.062 | 0.997 |
| rs9296068 | 6 | 32988695 | T | 0.048 | 1.000 |
| rs9469899 | 6 | 34793124 | A | 0.048 | 0.995 |
| rs4711748 | 6 | 43694598 | T | 0.052 | 0.990 |
| rs527510716 | 7 | 1944537 | C | 0.059 | 0.769 |
| rs11452686 | 7 | 20414110 | T | 0.05 | 0.857 |
| rs17621345 | 7 | 40875192 | A | 0.072 | 1.000 |
| rs1048169 | 9 | 19055965 | C | 0.061 | 0.998 |
| rs10122495 | 9 | 34049779 | T | 0.05 | 0.967 |
| rs1182 | 9 | 132576060 | A | 0.058 | 0.994 |
| rs141536087 | 10 | 854691 | GCGCA | 0.081 | 0.965 |
| rs1935581 | 10 | 90195149 | C | 0.048 | 0.998 |
| rs7094871 | 10 | 114712154 | G | 0.044 | 1.000 |
| rs1881502 | 11 | 1507512 | T | 0.058 | 0.998 |
| rs61890184 | 11 | 7547587 | A | 0.071 | 1.000 |
| rs547171081 | 11 | 47421962 | CGG | 0.047 | 0.902 |
| rs2277283 | 11 | 61908440 | C | 0.056 | 0.940 |
| rs12785905 | 11 | 66951965 | C | 0.116 | 0.901 |
| rs11290954 | 11 | 76260543 | AC | 0.061 | 0.997 |
| rs1800057 | 11 | 108143456 | G | 0.15 | 0.994 |
| rs138466039 | 11 | 125054793 | T | 0.281 | 0.951 |
| rs878987 | 11 | 134266372 | G | 0.064 | 0.988 |
| rs2066827 | 12 | 12871099 | T | 0.056 | 1.000 |
| rs10845938 | 12 | 14416918 | G | 0.057 | 1.000 |
| rs7968403 | 12 | 65012824 | T | 0.059 | 0.992 |
| rs5799921 | 12 | 90160530 | GA | 0.061 | 0.989 |
| rs7295014 | 12 | 133067989 | G | 0.052 | 0.971 |
| rs1004030 | 14 | 23305649 | T | 0.046 | 0.994 |
| rs11629412 | 14 | 37138294 | C | 0.057 | 0.999 |
| rs4924487 | 15 | 40922915 | C | 0.062 | 0.996 |
| rs33984059 | 15 | 56385868 | A | 0.176 | 1.000 |
| rs112293876 | 15 | 66764641 | C | 0.057 | 0.917 |
| rs11863709 | 16 | 57654576 | C | 0.148 | 0.933 |
| rs201158093 | 16 | 82178893 | TAA | 0.049 | 0.880 |
| rs28441558 | 17 | 7803118 | C | 0.151 | 1.000 |
| rs142444269 | 17 | 30098749 | C | 0.067 | 0.962 |
| rs2680708 | 17 | 56456120 | G | 0.046 | 1.000 |
| rs8093601 | 18 | 51772473 | C | 0.045 | 0.997 |
| rs28607662 | 18 | 53230859 | C | 0.075 | 1.000 |
| rs12956892 | 18 | 56746315 | T | 0.05 | 0.988 |
| rs533722308 | 18 | 60961193 | CT | 0.052 | 0.760 |
| rs10460109 | 18 | 73036165 | T | 0.044 | 0.997 |
| rs11666569 | 19 | 17214073 | C | 0.052 | 0.997 |
| rs118005503 | 19 | 32167803 | G | 0.09 | 0.949 |
| rs61088131 | 19 | 42700947 | T | 0.062 | 1.000 |
| rs11480453 | 20 | 31347512 | C | 0.046 | 0.955 |
| rs6091758 | 20 | 52455205 | G | 0.072 | 0.965 |
| rs9625483 | 22 | 28888939 | A | 0.134 | 1.000 |
| rs17321482 | 23 | 11482634 | C | 0.067 | 0.958 |
| rs17599629 | 1 | 150658287 | G | 0.065 | 1.000 |
| rs1218582 | 1 | 154834183 | G | 0.046 | 1.000 |
| rs4245739 | 1 | 204518842 | A | 0.092 | 1.000 |
| rs9287719 | 2 | 10710730 | C | 0.066 | 1.000 |
| rs9306895 | 2 | 20878153 | C | 0.077 | 1.000 |
| rs1465618 | 2 | 43553949 | T | 0.083 | 1.000 |
| rs721048 | 2 | 63131731 | A | 0.097 | 1.000 |
| rs10187424 | 2 | 85794297 | T | 0.074 | 1.000 |
| rs12621278 | 2 | 173311553 | A | 0.242 | 1.000 |
| rs2292884 | 2 | 238443226 | G | 0.061 | 0.998 |
| rs3771570 | 2 | 242382864 | T | 0.084 | 1.000 |
| rs2660753 | 3 | 87110674 | T | 0.12 | 1.000 |
| rs7611694 | 3 | 113275624 | A | 0.083 | 1.000 |
| rs10934853 | 3 | 128038373 | A | 0.099 | 1.000 |
| rs6763931 | 3 | 141102833 | A | 0.043 | 1.000 |
| rs10936632 | 3 | 170130102 | A | 0.097 | 0.995 |
| rs10009409 | 4 | 73855253 | T | 0.056 | 1.000 |
| rs1894292 | 4 | 74349158 | G | 0.062 | 1.000 |
| rs12500426 | 4 | 95514609 | A | 0.069 | 1.000 |
| rs17021918 | 4 | 95562877 | C | 0.085 | 1.000 |
| rs7679673 | 4 | 106061534 | C | 0.12 | 1.000 |
| rs2242652 | 5 | 1280028 | G | 0.16 | 0.971 |
| rs12653946 | 5 | 1895829 | T | 0.079 | 1.000 |
| rs2121875 | 5 | 44365545 | C | 0.048 | 1.000 |
| rs4713266 | 6 | 11219030 | C | 0.051 | 0.994 |
| rs7767188 | 6 | 30073776 | A | 0.054 | 1.000 |
| rs3096702 | 6 | 32192331 | A | 0.056 | 0.997 |
| rs3129859 | 6 | 32400939 | G | 0.06 | 1.000 |
| rs1983891 | 6 | 41536427 | T | 0.082 | 1.000 |
| rs9443189 | 6 | 76495882 | A | 0.064 | 0.992 |
| rs2273669 | 6 | 109285189 | G | 0.069 | 1.000 |
| rs339331 | 6 | 117210052 | T | 0.084 | 1.000 |
| rs1933488 | 6 | 153441079 | A | 0.076 | 1.000 |
| rs9364554 | 6 | 160833664 | T | 0.104 | 1.000 |
| rs12155172 | 7 | 20994491 | A | 0.093 | 1.000 |
| rs10486567 | 7 | 27976563 | G | 0.134 | 1.000 |
| rs56232506 | 7 | 47437244 | A | 0.054 | 0.987 |
| rs6465657 | 7 | 97816327 | C | 0.101 | 1.000 |
| rs2928679 | 8 | 23438975 | A | 0.053 | 1.000 |
| rs1512268 | 8 | 23526463 | T | 0.128 | 1.000 |
| rs11135910 | 8 | 25892142 | T | 0.078 | 1.000 |
| rs12543663 | 8 | 127924659 | C | 0.111 | 1.000 |
| rs10086908 | 8 | 128011937 | T | 0.126 | 1.000 |
| rs183373024 | 8 | 128104117 | G | 1.068 | 0.926 |
| rs16901979 | 8 | 128124916 | A | 0.445 | 1.000 |
| rs620861 | 8 | 128335673 | G | 0.139 | 1.000 |
| rs6983267 | 8 | 128413305 | G | 0.2 | 1.000 |
| rs1447295 | 8 | 128485038 | A | 0.345 | 1.000 |
| rs17694493 | 9 | 22041998 | G | 0.073 | 1.000 |
| rs76934034 | 10 | 46082985 | T | 0.115 | 1.000 |
| rs10993994 | 10 | 51549496 | T | 0.208 | 1.000 |
| rs3850699 | 10 | 104414221 | A | 0.07 | 1.000 |
| rs4962416 | 10 | 126696872 | C | 0.059 | 1.000 |
| rs7127900 | 11 | 2233574 | A | 0.17 | 1.000 |
| rs7931342 | 11 | 68994497 | G | 0.157 | 1.000 |
| rs11568818 | 11 | 102401661 | T | 0.074 | 1.000 |
| rs11214775 | 11 | 113807181 | G | 0.071 | 1.000 |
| rs80130819 | 12 | 48419618 | A | 0.096 | 1.000 |
| rs10875943 | 12 | 49676010 | C | 0.069 | 1.000 |
| rs902774 | 12 | 53273904 | A | 0.126 | 1.000 |
| rs1270884 | 12 | 114685571 | A | 0.07 | 1.000 |
| rs8008270 | 14 | 53372330 | C | 0.083 | 1.000 |
| rs7141529 | 14 | 69126744 | C | 0.051 | 1.000 |
| rs8014671 | 14 | 71092256 | G | 0.047 | 1.000 |
| rs684232 | 17 | 618965 | C | 0.083 | 1.000 |
| rs11649743 | 17 | 36074979 | G | 0.122 | 1.000 |
| rs4430796 | 17 | 36098040 | A | 0.197 | 0.984 |
| rs138213197 | 17 | 46805705 | T | 1.348 | 1.000 |
| rs11650494 | 17 | 47345186 | A | 0.099 | 1.000 |
| rs1859962 | 17 | 69108753 | G | 0.161 | 1.000 |
| rs7241993 | 18 | 76773973 | C | 0.076 | 1.000 |
| rs8102476 | 19 | 38735613 | C | 0.09 | 0.999 |
| rs11672691 | 19 | 41985587 | G | 0.092 | 1.000 |
| rs2735839 | 19 | 51364623 | G | 0.167 | 1.000 |
| rs12480328 | 20 | 49527922 | T | 0.107 | 0.999 |
| rs2427345 | 20 | 61015611 | C | 0.045 | 1.000 |
| rs6062509 | 20 | 62362563 | T | 0.078 | 1.000 |
| rs1041449 | 21 | 42901421 | G | 0.051 | 0.990 |
| rs58133635 | 22 | 40471188 | T | 0.068 | 1.000 |
| rs5759167 | 22 | 43500212 | G | 0.142 | 1.000 |
| rs2405942 | 23 | 9814135 | A | 0.049 | 1.000 |
| rs5945619 | 23 | 51241672 | C | 0.104 | 1.000 |
| rs2807031 | 23 | 52896949 | C | 0.058 | 1.000 |
| rs5919432 | 23 | 67021550 | T | 0.043 | 1.000 |
| rs6625711 | 23 | 70139850 | A | 0.008 | 0.840 |

^a^ SNP = single nucleotide polymorphism; chr = chromosome; position = human genome build 37 position; OR = per (effect) allele odds ratio association with breast cancer risk estimated from the Prostate Cancer Association Group to Investigate Cancer Associated Associations in the Genome (PRACTICAL) consortium population-based data.

**Supplementary Table 5**: Quartile-specific PRS associations for the breast cancer PRS with overall breast cancer risk and the prostate cancer PRS with prostate cancer risk.

| PRS | Association | Quartile^a^ | ***BRCA1* carriers** | | | ***BRCA2* carriers** | | |
| --- | --- | --- | --- | --- | --- | --- | --- | --- |
|  |  |  | Controls | Cases | OR (95% CI) | Controls | Cases | OR (95% CI) |
| PRS_BC_ | PRS_BC_ association with breast cancer risk | 1st quartile | 108 | 3 | 1.00 [reference] | 221 | 40 | 1.00 [reference] |
|  |  | 2nd quartile | 83 | 11 | 5.68 (1.48-21.85) | 245 | 56 | 1.22 (0.77-1.92) |
|  |  | 3rd quartile | 92 | 9 | 4.04 (1.02-15.99) | 236 | 57 | 1.35 (0.86-2.11) |
|  |  | 4th quartile | 97 | 10 | 4.12 (1.12-15.23) | 231 | 91 | 2.08 (1.36-3.20) |
| PRS_ER-_ | PRS_ER-_ association with breast cancer risk | 1st quartile | 98 | 6 | 1.00 [reference] | 231 | 50 | 1.00 [reference] |
|  |  | 2nd quartile | 103 | 11 | 1.88 (0.69-5.09) | 225 | 46 | 0.89 (0.57-1.40) |
|  |  | 3rd quartile | 82 | 6 | 1.24 (0.43-3.55) | 246 | 74 | 1.37 (0.91-2.07) |
|  |  | 4th quartile | 97 | 10 | 1.74 (0.60-5.01) | 231 | 74 | 1.54 (1.01-2.32) |
| PRS_ER+_ | PRS_ER+_ association with breast cancer risk | 1st quartile | 110 | 3 | 1.00 [reference] | 219 | 43 | 1.00 [reference] |
|  |  | 2nd quartile | 83 | 9 | 4.41 (1.10-17.73) | 245 | 51 | 1.00 (0.64-1.58) |
|  |  | 3rd quartile | 88 | 11 | 5.09 (1.33-19.40) | 240 | 58 | 1.24 (0.80-1.93) |
|  |  | 4th quartile | 99 | 10 | 4.00 (1.07-14.95) | 229 | 92 | 1.96 (1.30-2.96) |
| PRS_PC_ | PRS_PC_ association with prostate cancer risk | 1st quartile | 88 | 9 | 1.00 [reference] | 241 | 17 | 1.00 [reference] |
|  |  | 2nd quartile | 95 | 15 | 1.51 (0.61-3.73) | 233 | 34 | 2.14 (1.16-3.97) |
|  |  | 3rd quartile | 107 | 22 | 1.88 (0.81-4.35) | 221 | 32 | 2.22 (1.19-4.13) |
|  |  | 4th quartile | 90 | 24 | 2.80 (1.18-6.63) | 238 | 58 | 3.61 (2.01-6.49) |

^a^ PRS quartile thresholds were determined in the combined *BRCA1* and *BRCA2* carrier controls. PRC_BC_ = overall breast cancer PRS; PRS_ER-_ = ER-negative breast cancer PRS; PRS_ER+_ = ER-positive breast cancer PRS; PRS_PC_ = prostate cancer PRS.OR = odds ratio per PRS standard deviation, estimated from a multinomial logistic regression; CI = confidence interval.

**Supplementary Table 6**: Associations of the breast cancer PRS with breast cancer risk in the combined sample of *BRCA1* and *BRCA2* carriers.

| PRS investigated and outcome^a^ | **PRS_BC_** | | | | | **PRS_ER-_** | | | | | **PRS_ER+_** | | | | | |
| --- | --- | --- | --- | --- | --- | --- | --- | --- | --- | --- | --- | --- | --- | --- | --- | --- |
|  | Controls | Cases | OR (95% CI) | P | P_LRT_ | Controls | Cases | OR (95% CI) | P | P_LRT_ | Controls | Cases | OR (95% CI) | P | P_LRT_ | |
| PRS association with breast cancer risk |  |  |  |  |  |  |  |  |  |  |  |  |  |  |  | |
| 1st quartile^b^ | 329 | 43 | 1.00 [reference] |  |  | 329 | 56 | 1.00 [reference] |  |  | 329 | 46 | 1.00 [reference] |  |  | |
| 2nd quartile^b^ | 328 | 67 | 1.47 (0.97, 2.23) |  |  | 328 | 57 | 1.02 (0.68, 1.54) |  |  | 328 | 60 | 1.20 (0.78, 1.82) |  |  | |
| 3rd quartile^b^ | 328 | 66 | 1.52 (1.00, 2.30) |  |  | 328 | 80 | 1.39 (0.96, 2.02) |  |  | 328 | 69 | 1.45 (0.96, 2.19) |  |  | |
| 4th quartile^b^ | 328 | 101 | 2.24 (1.51, 3.31) |  |  | 328 | 84 | 1.57 (1.07, 2.30) |  |  | 328 | 102 | 2.10 (1.43, 3.08) |  |  | |
| Continuous^b^ | 1313 | 277 | 1.33 (1.17, 1.51) | <0.001 |  | 1313 | 277 | 1.21 (1.06, 1.38) | 0.004 |  | 1313 | 277 | 1.33 (1.17, 1.51 | <0.001 |  | |
| PRS association with ER-specific breast cancer risk^c^ |  |  |  |  |  |  |  |  |  |  |  |  |  |  |  | |
| Controls | 1313 | -- | 1.00 [reference] |  |  | 1313 | -- | 1.00 [reference] |  |  | 1313 | -- | 1.00 [reference] |  |  | |
| ER-negative | -- | 9 | 0.61 (0.37, 1.03) | 0.063 |  | -- | 9 | 0.49 (0.27, 0.90) | 0.02 |  | -- | 9 | 0.66 (0.41, 1.08) | 0.10 |  | |
| ER-positive | -- | 199 | 1.35 (1.17, 1.56) | <0.001 |  | -- | 199 | 1.28 (1.10, 1.48) | 0.001 |  | -- | 199 | 1.34 (1.16, 1.54) | <0.001 |  | |
| ER-status unknown | -- | 69 | 1.39 (1.11, 1.73) | 0.004 |  | -- | 69 | 1.15 (0.90, 1.46) | 0.26 |  | -- | 69 | 1.42 (1.14, 1.77) | 0.002 |  | |
| PRS association with grade-specific breast cancer risk^d^ |  |  |  |  |  |  |  |  |  |  |  |  |  |  |  | |
| Controls | 1313 | -- | 1.00 [reference] |  |  | 1313 | -- | 1.00 [reference] |  |  | 1313 | -- | 1.00 [reference] |  |  | |
| Grade 1 | -- | 12 | 1.33 (0.78, 2.27) | 0.30 |  | -- | 12 | 1.35 (0.82, 2.23) | 0.24 |  | -- | 12 | 1.31 (0.79, 2.19) | 0.30 |  | |
| Grade 2 | -- | 74 | 1.26 (1.02, 1.55) | 0.03 |  | -- | 74 | 1.20 (0.94, 1.52) | 0.14 |  | -- | 74 | 1.25 (1.02, 1.54) | 0.03 |  | |
| Grade 3 | -- | 110 | 1.26 (1.04, 1.51) | 0.02 |  | -- | 110 | 1.14 (0.94, 1.37) | 0.17 |  | -- | 110 | 1.26 (1.04, 1.52) | 0.02 |  | |
| Grade unknown | -- | 81 | 1.49 (1.21, 1.85) | <0.001 |  | -- | 81 | 1.30 (1.04, 1.63) | 0.02 |  | -- | 81 | 1.51 (1.22, 1.86) | <0.001 |  | |
| Case-only: grade 1+2 vs grade 3^e^ | 86 | 110 | 1.00 (0.75, 1.32) | 1.00 |  | 86 | 110 | 0.96 (0.73, 1.26) | 0.76 |  | 86 | 110 | 1.00 (0.75, 1.33) | 1.00 |  | |
| PRS x Age interaction^f^ |  |  |  |  |  |  |  |  |  |  |  |  |  |  |  | |
| PRS | 1313 | 277 | 1.35 (0.78, 2.33) | 0.29 | 0.82 | 1313 | 277 | 1.18 (0.69, 2.02) | 0.55 | 0.92 | 1313 | 277 | 1.39 (0.81, 2.41) | 0.23 | 0.76 | |
| PRS x Age |  |  | 1.00 (0.99, 1.01) | 0.96 |  |  |  | 1.00 (0.99, 1.01) | 0.93 |  |  |  | 1.00 (0.99, 1.01) | 0.86 |  |  |
| Gene pathogenic mutation class^g^ |  |  |  |  |  |  |  |  |  |  |  |  |  |  | |  |
| Class I | 1111 | 246 | 1.31 (1.14, 1.50) | <0.001 | 0.44 | 1111 | 246 | 1.20 (1.04, 1.38) | 0.01 | 0.16 | 1111 | 246 | 1.31 (1.14, 1.50) | <0.001 | 0.57 | |
| Class II | 91 | 15 | 1.48 (0.88, 2.52) | 0.14 |  | 91 | 15 | 1.18 (0.71, 1.98) | 0.52 |  | 91 | 15 | 1.51 (0.88, 2.58) | 0.13 |  |  |

^a^ PRC_BC_ = overall breast cancer PRS; PRS_ER-_ = ER-negative breast cancer PRS; PRS_ER+_ = ER-positive breast cancer PRS; ER = estrogen-receptor. OR = odds ratio per PRS standard deviation, estimated from a multinomial logistic regression (unless otherwise stated); CI = confidence interval. P value was calculated using a 2-sided Wald test. P_LRT_ = P values were calculated using a 2-sided likelihood ratio test. The likelihood ratio test compared the model that estimated the interaction term with a nested model that omitting the interaction term.

^b^ PRS quartile thresholds were determined in the combined *BRCA1* and *BRCA2* carrier controls. The continuous test shows the per PRS standard deviation associations, estimated from a multinomial logistic regression model assuming a continuous PRS.

^c^ The ER-specific breast cancer ORs were estimated by partitioning breast cancer status into distinct multinomial outcomes for (i) ER-negative, (ii) ER-positive, or (iii) ER-status unknown.

^d^ The breast cancer grade specific ORs were estimated by partitioning breast cancer status into multinomial outcomes for (i) grade 1, (ii) grade 2, (iii) grade 3, or (iv) grade unknown.

^e^ The case-only breast cancer grade analysis was a logistic regression considering grade 1 and grade 2 breast cancers combined as “controls” and grade 3 breast cancers as “cases”.

^f^ Age in years. The PRS term is applicable at age 0-years and the PRSxAge interaction term is a per-year effect.

^g^ “Class I” pathogenic variant = loss-of-function pathogenic variants expected to result in unstable or no protein; “class II” pathogenic variant = pathogenic variants likely to yield stable mutant proteins.

Association estimates adjusted for family history of (male and female) breast cancer in first- and second-degree relatives are presented in **Supplementary Table 8** (male breast cancer FH adjusted) and **Supplementary Table 9** (female breast cancer FH adjusted). Associations for *BRCA1* and *BRCA2* carriers analysed separately are presented in **Table 1** and **Table 3**.

**Supplementary Table 7**: Associations of the prostate cancer PRS with prostate cancer risk in the combined sample of *BRCA1* and *BRCA2* carriers.

| PRS investigated and outcome^a^ | **Controls** | **Cases** | **OR (95% CI)** | **P** | **P_LRT_** |
| --- | --- | --- | --- | --- | --- |
| PRS association with prostate cancer risk |  |  |  |  |  |
| 1st quartile^b^ | 329 | 26 | 1.00 [reference] |  |  |
| 2nd quartile^b^ | 328 | 49 | 1.92 (1.16, 3.20) |  |  |
| 3rd quartile^b^ | 328 | 54 | 2.10 (1.28, 3.45) |  |  |
| 4th quartile^b^ | 328 | 82 | 3.35 (2.06, 5.42) |  |  |
| Continuous^b^ | 1313 | 211 | 1.63 (1.40, 1.90) | <0.001 |  |
| PRS association with Gleason score (GS) specific prostate cancer risk^c^ |  |  |  |  |  |
| Controls | 1313 | -- | 1.00 [reference] |  |  |
| GS < 7 | -- | 53 | 1.46 (1.11, 1.93) | 0.007 |  |
| GS ≥ 7 | -- | 103 | 1.75 (1.41, 2.16) | <0.001 |  |
| GS unknown | -- | 55 | 1.59 (1.23, 2.05) | <0.001 |  |
| Case-only analysis: GS≥7 vs GS<7^d^ | 53 | 103 | 1.14 (0.82, 1.58) | 0.43 |  |
| PRS x Age interaction^e^ |  |  |  |  |  |
| PRS | 1313 | 211 | 1.46 (0.75, 2.85) | 0.27 | 0.93 |
| PRS x Age |  |  | 1.00 (0.99, 1.01) | 0.74 |  |
| Gene pathogenic mutation class^f^ |  |  |  |  |  |
| Class I | 1111 | 174 | 1.57 (1.33, 1.85) | <0.001 | 0.42 |
| Class II | 91 | 19 | 2.52 (1.28, 4.96) | 0.007 |  |

^a^ PRS_PC_ = prostate cancer PRS; GS = Gleason score. OR = odds ratio per PRS standard deviation, estimated from a multinomial logistic regression (unless otherwise stated); CI = confidence interval;

PRS quartile thresholds were determined in the combined *BRCA1* and *BRCA2* carrier controls. P value was calculated using a 2-sided Wald test. P_LRT_ = P values were calculated using a 2-sided likelihood ratio test. The likelihood ratio test compared the model that estimated the interaction term with a nested model that omitting the interaction term.

^b^ PRS quartile thresholds were determined in the combined *BRCA1* and *BRCA2* carrier controls. The continuous test shows the per PRS standard deviation associations, estimated from a multinomial logistic regression model assuming a continuous PRS.

^c^ The Gleason score prostate cancer ORs were estimated by partitioning prostate cancer status into distinct multinomial outcomes for (i) GS < 7, (ii) GS ≥ 7, or (iii) GS unknown.

^d^ The case-only prostate cancer analysis was a logistic regression considering GS < 7 prostate cancers as “controls” and GS ≥ 7 prostate cancers as “cases”.

^e^ Age in years. The PRS term is applicable at age 0-years and the PRSxAge interaction term is a per-year effect.

^f^ “Class I” pathogenic variant = loss-of-function pathogenic variants expected to result in unstable or no protein; “class II” pathogenic variant = pathogenic variants likely to yield stable mutant proteins.

Association estimates adjusted for family history of prostate cancer in first- and second-degree relatives are presented in **Supplementary Table 10**. Associations for *BRCA1* and *BRCA2* carriers analysed separately are presented in **Table 2** and **Table 3**.

**Supplementary Table 8**: Associations of the breast cancer PRS with breast cancer risk adjusted for family history of breast cancer in male first- and second-degree relatives.

| **PRS investigated**  **and outcome^a^** | ***BRCA1* and *BRCA2* carriers** | | | | ***BRCA1* carriers** | | | | ***BRCA2* carriers** | | | |
| --- | --- | --- | --- | --- | --- | --- | --- | --- | --- | --- | --- | --- |
|  | Controls | Cases | OR (95% CI) | P | Controls | Cases | OR (95% CI) | P | Controls | Cases | OR (95% CI) | P |
| **PRS_BC_** | | | | | | | | | | | | |
| PRS_BC_ association with breast cancer risk | | | | | | | | | | | | |
| 1st quartile^b^ | 329 | 43 | 1.00 [reference] |  | 108 | 3 | 1.00 [reference] |  | 221 | 40 | 1.00 [reference] |  |
| 2nd quartile^b^ | 328 | 67 | 1.45 (0.96, 2.21) |  | 83 | 11 | 5.66 (1.39, 23.00) |  | 245 | 56 | 1.20 (0.76, 1.89) |  |
| 3rd quartile^b^ | 328 | 66 | 1.52 (1.00, 2.31) |  | 92 | 9 | 4.06 (1.01, 16.35) |  | 236 | 57 | 1.36 (0.87, 2.13) |  |
| 4th quartile^b^ | 328 | 101 | 2.26 (1.53, 3.36) |  | 97 | 10 | 4.06 (1.09, 15.08) |  | 231 | 91 | 2.11 (1.37, 3.25) |  |
| Continuous^b^ | 1313 | 277 | 1.33 (1.17, 1.51) | <0.001 | 380 | 33 | 1.39 (1.05, 1.84) | 0.02 | 933 | 244 | 1.33 (1.15, 1.52) | <0.001 |
| PRS_BC_ association with ER-specific breast cancer risk^c^ | | | | | | | | | | | | |
| Controls | 1313 | -- | 1.00 [reference] |  | 380 | -- | 1.00 [reference] |  | 933 | -- | 1.00 [reference] |  |
| ER-negative | -- | 9 | 0.61 (0.36, 1.04) | 0.07 | -- | 2 | 0.26 (0.03, 2.09) | 0.20 | -- | 7 | 0.64 (0.35, 1.17) | 0.15 |
| ER-positive | -- | 199 | 1.35 (1.17, 1.56) | <0.001 | -- | 21 | 1.81 (1.29, 2.55) | <0.001 | -- | 178 | 1.31 (1.12, 1.53) | <0.001 |
| ER-status unknown | -- | 69 | 1.40 (1.11, 1.75) | 0.004 | -- | 10 | 0.98 (0.66, 1.45) | 0.90 | -- | 59 | 1.52 (1.16, 1.99) | 0.002 |
| PRS_BC_ association with grade-specific breast cancer risk^d^ | | | | | | | | | | | | |
| Controls | 1313 | -- | 1.00 [reference] |  | 380 | -- | 1.00 [reference] |  | 933 | -- | 1.00 [reference] |  |
| Grade 1 | -- | 12 | 1.30 (0.75, 2.28) | 0.35 | -- | 1 | 1.01 (0.59, 1.71)^f^ | 0.98 | -- | 11 | 1.30 (0.70, 2.38) | 0.40 |
| Grade 2 | -- | 74 | 1.26 (1.02, 1.55) | 0.03 | -- | 6 |  |  | -- | 68 | 1.30 (1.05, 1.61) | 0.02 |
| Grade 3 | -- | 110 | 1.25 (1.04, 1.51) | 0.02 | -- | 12 | 1.54 (1.02, 2.32) | 0.04 | -- | 98 | 1.21 (0.99, 1.49) | 0.06 |
| Grade unknown | -- | 81 | 1.52 (1.23, 1.90) | <0.001 | -- | 14 | 1.46 (0.92, 2.31) | 0.11 | -- | 67 | 1.59 (1.23, 2.06) | <0.001 |
| Case-only: grade 1+2  vs grade 3^e^ | 86 | 110 | 1.01 (0.76, 1.34) | 0.96 | 7 | 12 | 8.78 (0.60, 129.16) | 0.11 | 79 | 98 | 0.95 (0.71, 1.28) | 0.73 |
| **PRS_ER-_** | | | | | | | | | | | | |
| PRS_ER-_ association with breast cancer risk | | | | | | | | | | | | |
| 1st quartile^b^ | 329 | 56 | 1.00 [reference] |  | 98 | 6 | 1.00 [reference] |  | 231 | 50 | 1.00 [reference] |  |
| 2nd quartile^b^ | 328 | 57 | 1.45 (0.96, 2.21) |  | 103 | 11 | 5.66 (1.39, 23.00) |  | 225 | 46 | 1.20 (0.76, 1.89) |  |
| 3rd quartile^b^ | 328 | 80 | 1.52 (1.00, 2.31) |  | 82 | 6 | 4.06 (1.01, 16.35) |  | 246 | 74 | 1.36 (0.87, 2.13) |  |
| 4th quartile^b^ | 328 | 84 | 2.26 (1.53, 3.36) |  | 97 | 10 | 4.06 (1.09, 15.08) |  | 231 | 74 | 2.11 (1.37, 3.25) |  |
| Continuous^b^ | 1313 | 277 | 1.21 (1.06, 1.38) | 0.004 | 380 | 33 | 1.12 (0.79, 1.59) | 0.53 | 933 | 244 | 1.23 (1.07, 1.42) | 0.004 |
| PRS_ER-_ association with ER-specific breast cancer risk^c^ | | | | | | | | | | | | |
| Controls | 1313 | -- | 1.00 [reference] |  | 380 | -- | 1.00 [reference] |  | 933 | -- | 1.00 [reference] |  |
| ER-negative | -- | 9 | 0.50 (0.27, 0.91) | 0.02 | -- | 2 | 0.38 (0.06, 2.37) | 0.30 | -- | 7 | 0.51 (0.26, 1.01) | 0.05 |
| ER-positive | -- | 199 | 1.28 (1.11, 1.48) | 0.001 | -- | 21 | 1.47 (0.96, 2.23) | 0.07 | -- | 178 | 1.26 (1.08, 1.47) | 0.004 |
| ER-status unknown | -- | 69 | 1.14 (0.89, 1.46) | 0.29 | -- | 10 | 0.78 (0.47, 1.30) | 0.33 | -- | 59 | 1.27 (0.95, 1.70) | 0.11 |
| PRS_ER-_ association with grade-specific breast cancer risk^d^ | | | | | | | | | | | | |
| Controls | 1313 | -- | 1.00 [reference] |  | 380 | -- | 1.00 [reference] |  | 933 | -- | 1.00 [reference] |  |
| Grade 1 | -- | 12 | 1.34 (0.79, 2.26) | 0.27 | -- | 1 | 0.88 (0.46, 1.68)^f^ | 0.70 | -- | 11 | 1.35 (0.76, 2.40) | 0.30 |
| Grade 2 | -- | 74 | 1.20 (0.94, 1.52) | 0.14 | -- | 6 |  |  | -- | 68 | 1.24 (0.96, 1.60) | 0.09 |
| Grade 3 | -- | 110 | 1.14 (0.95, 1.37) | 0.17 | -- | 12 | 1.36 (0.72, 2.56) | 0.35 | -- | 98 | 1.11 (0.91, 1.35) | 0.30 |
| Grade unknown | -- | 81 | 1.31 (1.04, 1.64) | 0.02 | -- | 14 | 1.06 (0.65, 1.74) | 0.81 | -- | 67 | 1.45 (1.10, 1.91) | 0.009 |
| Case-only: grade 1+2  vs grade 3^e^ | 86 | 110 | 0.96 (0.72, 1.27) | 0.78 | 7 | 12 | 10.30 (0.55, 192.82) | 0.12 | 79 | 98 | 0.91 (0.67, 1.23) | 0.53 |
| **PRS_ER+_** | | | | | | | | | | | | |
| PRS_ER+_ association with breast cancer risk | | | | | | | | | | | | |
| 1st quartile^b^ | 329 | 46 | 1.00 [reference] |  | 110 | 3 | 1.00 [reference] |  | 219 | 43 | 1.00 [reference] |  |
| 2nd quartile^b^ | 328 | 60 | 1.17 (0.77, 1.79) |  | 83 | 9 | 4.37 (1.03, 18.49) |  | 245 | 51 | 0.98 (0.62, 1.55) |  |
| 3rd quartile^b^ | 328 | 69 | 1.45 (0.96, 2.18) |  | 88 | 11 | 5.11 (1.33, 19.62) |  | 240 | 58 | 1.24 (0.80, 1.93) |  |
| 4th quartile^b^ | 328 | 102 | 2.10 (1.43, 3.10) |  | 99 | 10 | 3.92 (1.04, 14.70) |  | 229 | 92 | 1.97 (1.29, 2.99) |  |
| Continuous^b^ | 1313 | 277 | 1.33 (1.17, 1.51) | <0.001 | 380 | 33 | 1.39 (1.06, 1.82) | 0.02 | 933 | 244 | 1.33 (1.16, 1.53) | <0.001 |
| PRS_ER+_ association with ER-specific breast cancer risk^c^ | | | | | | | | | | | | |
| Controls | 1313 | -- | 1.00 [reference] |  | 380 | -- | 1.00 [reference] |  | 933 | -- | 1.00 [reference] |  |
| ER-negative | -- | 9 | 0.66 (0.40, 1.09) | 0.11 | -- | 2 | 0.24 (0.03, 1.83) | 0.17 | -- | 7 | 0.69 (0.38, 1.24) | 0.21 |
| ER-positive | -- | 199 | 1.34 (1.16, 1.54) | <0.001 | -- | 21 | 1.78 (1.28, 2.48) | <0.001 | -- | 178 | 1.30 (1.11, 1.52) | 0.001 |
| ER-status unknown | -- | 69 | 1.43 (1.15, 1.79) | 0.002 | -- | 10 | 0.99 (0.68, 1.46) | 0.98 | -- | 59 | 1.55 (1.19, 2.02) | 0.001 |
| PRS_ER+_ association with grade-specific breast cancer risk^d^ | | | | | | | | | | | | |
| Controls | 1313 | -- | 1.00 [reference] |  | 380 | -- | 1.00 [reference] |  | 933 | -- | 1.00 [reference] |  |
| Grade 1 | -- | 12 | 1.29 (0.76, 2.19) | 0.35 | -- | 1 | 1.01 (0.61, 1.67)^f^ | 0.97 | -- | 11 | 1.28 (0.72, 2.29) | 0.40 |
| Grade 2 | -- | 74 | 1.26 (1.02, 1.54) | 0.03 | -- | 6 |  |  | -- | 68 | 1.30 (1.05, 1.60) | 0.02 |
| Grade 3 | -- | 110 | 1.25 (1.04, 1.51) | 0.02 | -- | 12 | 1.49 (1.04, 2.14) | 0.03 | -- | 98 | 1.22 (1.00, 1.50) | 0.06 |
| Grade unknown | -- | 81 | 1.54 (1.24, 1.91) | <0.001 | -- | 14 | 1.50 (0.95, 2.38) | 0.08 | -- | 67 | 1.59 (1.23, 2.05) | <0.001 |
| Case-only: grade 1+2  vs grade 3^e^ | 86 | 110 | 1.01 (0.75, 1.35) | 0.95 | 7 | 12 | 7.64 (0.58, 100.45) | 0.12 | 79 | 98 | 0.95 (0.71, 1.29) | 0.76 |

^a^ PRC_BC_ = overall breast cancer PRS; PRS_ER-_ = ER-negative breast cancer PRS; PRS_ER+_ = ER-positive breast cancer PRS; ER = estrogen-receptor. OR = odds ratio per PRS standard deviation, estimated from a multinomial logistic regression (unless otherwise stated); CI = confidence interval. P value was calculated using a 2-sided Wald test.
^b^ PRS quartile thresholds were determined in the combined *BRCA1* and *BRCA2* carrier controls. The continuous test shows the per PRS standard deviation associations, estimated from a multinomial logistic regression model assuming a continuous PRS.
^c^ The ER-specific breast cancer ORs were estimated by partitioning breast cancer status into distinct multinomial outcomes for (i) ER-negative, (ii) ER-positive, or (iii) ER-status unknown.
^d^ The breast cancer grade specific ORs were estimated by partitioning breast cancer status into multinomial outcomes for (i) grade 1, (ii) grade 2, (iii) grade 3, or (iv) grade unknown.
^e^ The case-only breast cancer grade analysis was a logistic regression considering grade 1 and grade 2 breast cancers combined as “controls” and grade 3 breast cancers as “cases”.
^f^ Grade 1 and grade 2 combined for *BRCA1* carriers (to ensure adequate sample size to estimate associations).

**Supplementary Table 9**: Associations of the breast cancer PRS with breast cancer risk adjusted for family history of breast cancer in female first- and second-degree relatives.

| **PRS investigated**  **and outcome^a^** | ***BRCA1* and *BRCA2* carriers** | | | | ***BRCA1* carriers** | | | | ***BRCA2* carriers** | | | |
| --- | --- | --- | --- | --- | --- | --- | --- | --- | --- | --- | --- | --- |
|  | Controls | Cases | OR (95% CI) | P | Controls | Cases | OR (95% CI) | P | Controls | Cases | OR (95% CI) | P |
| **PRS_BC_** | | | | | | | | | | | | |
| PRS_BC_ association with breast cancer risk | | | | | | | | | | | | |
| 1st quartile^b^ | 329 | 43 | 1.00 [reference] |  | 108 | 3 | 1.00 [reference] |  | 221 | 40 | 1.00 [reference] |  |
| 2nd quartile^b^ | 328 | 67 | 1.55 (1.02, 2.37) |  | 83 | 11 | 6.05 (1.51, 24.32) |  | 245 | 56 | 1.29 (0.81, 2.06) |  |
| 3rd quartile^b^ | 328 | 66 | 1.59 (1.05, 2.43) |  | 92 | 9 | 4.17 (1.02, 17.03) |  | 236 | 57 | 1.42 (0.90, 2.25) |  |
| 4th quartile^b^ | 328 | 101 | 2.43 (1.62, 3.63) |  | 97 | 10 | 4.58 (1.18, 17.83) |  | 231 | 91 | 2.30 (1.48, 3.57) |  |
| Continuous^b^ | 1313 | 277 | 1.36 (1.20, 1.55) | <0.001 | 380 | 33 | 1.44 (1.07, 1.95) | 0.02 | 933 | 244 | 1.36 (1.18, 1.57) | <0.001 |
| PRS_BC_ association with ER-specific breast cancer risk^c^ | | | | | | | | | | | | |
| Controls | 1313 | -- | 1.00 [reference] |  | 380 | -- | 1.00 [reference] |  | 933 | -- | 1.00 [reference] |  |
| ER-negative | -- | 9 | 0.63 (0.37, 1.10) | 0.10 | -- | 2 | 0.24 (0.03, 1.84) | 0.17 | -- | 7 | 0.66 (0.36, 1.23) | 0.19 |
| ER-positive | -- | 199 | 1.38 (1.19, 1.60) | <0.001 | -- | 21 | 1.97 (1.37, 2.82) | <0.001 | -- | 178 | 1.34 (1.14, 1.57) | <0.001 |
| ER-status unknown | -- | 69 | 1.41 (1.12, 1.77) | 0.003 | -- | 10 | 1.02 (0.68, 1.54) | 0.91 | -- | 59 | 1.53 (1.17, 1.99) | 0.002 |
| PRS_BC_ association with grade-specific breast cancer risk^d^ | | | | | | | | | | | | |
| Controls | 1313 | -- | 1.00 [reference] |  | 380 | -- | 1.00 [reference] |  | 933 | -- | 1.00 [reference] |  |
| Grade 1 | -- | 12 | 1.33 (0.76, 2.35) | 0.32 | -- | 1 | 1.04 (0.63, 1.72)^f^ | 0.88 | -- | 11 | 1.33 (0.73, 2.45) | 0.35 |
| Grade 2 | -- | 74 | 1.29 (1.04, 1.59) | 0.02 | -- | 6 |  |  | -- | 68 | 1.33 (1.07, 1.66) | 0.01 |
| Grade 3 | -- | 110 | 1.29 (1.06, 1.56) | 0.009 | -- | 12 | 1.60 (1.04, 2.46) | 0.03 | -- | 98 | 1.25 (1.02, 1.54) | 0.03 |
| Grade unknown | -- | 81 | 1.52 (1.23, 1.89) | <0.001 | -- | 14 | 1.54 (0.95, 2.51) | 0.08 | -- | 67 | 1.57 (1.22, 2.02) | <0.001 |
| Case-only: grade 1+2  vs grade 3^e^ | 86 | 110 | 1.02 (0.77, 1.36) | 0.87 | 7 | 12 | 16.03 (0.61, 421.59) | 0.10 | 79 | 98 | 0.97 (0.72, 1.30) | 0.83 |
| **PRS_ER-_** | | | | | | | | | | | | |
| PRS_ER-_ association with breast cancer risk | | | | | | | | | | | | |
| 1st quartile^b^ | 329 | 56 | 1.00 [reference] |  | 98 | 6 | 1.00 [reference] |  | 231 | 50 | 1.00 [reference] |  |
| 2nd quartile^b^ | 328 | 57 | 1.55 (1.02, 2.37) |  | 103 | 11 | 6.05 (1.51, 24.32) |  | 225 | 46 | 1.29 (0.81, 2.06) |  |
| 3rd quartile^b^ | 328 | 80 | 1.59 (1.05, 2.43) |  | 82 | 6 | 4.17 (1.02, 17.03) |  | 246 | 74 | 1.42 (0.90, 2.25) |  |
| 4th quartile^b^ | 328 | 84 | 2.43 (1.62, 3.63) |  | 97 | 10 | 4.58 (1.18, 17.83) |  | 231 | 74 | 2.30 (1.48, 3.57) |  |
| Continuous^b^ | 1313 | 277 | 1.23 (1.08, 1.40) | 0.001 | 380 | 33 | 1.14 (0.80, 1.63) | 0.47 | 933 | 244 | 1.25 (1.09, 1.45) | 0.002 |
| PRS_ER-_ association with ER-specific breast cancer risk^c^ | | | | | | | | | | | | |
| Controls | 1313 | -- | 1.00 [reference] |  | 380 | -- | 1.00 [reference] |  | 933 | -- | 1.00 [reference] |  |
| ER-negative | -- | 9 | 0.52 (0.28, 0.95) | 0.03 | -- | 2 | 0.17 (0.02, 1.19) | 0.07 | -- | 7 | 0.54 (0.28, 1.06) | 0.07 |
| ER-positive | -- | 199 | 1.31 (1.13, 1.51) | <0.001 | -- | 21 | 1.55 (1.01, 2.37) | 0.04 | -- | 178 | 1.28 (1.09, 1.50) | 0.002 |
| ER-status unknown | -- | 69 | 1.16 (0.91, 1.47) | 0.24 | -- | 10 | 0.79 (0.47, 1.32) | 0.37 | -- | 59 | 1.28 (0.96, 1.70) | 0.09 |
| PRS_ER-_ association with grade-specific breast cancer risk^d^ | | | | | | | | | | | | |
| Controls | 1313 | -- | 1.00 [reference] |  | 380 | -- | 1.00 [reference] |  | 933 | -- | 1.00 [reference] |  |
| Grade 1 | -- | 12 | 1.37 (0.81, 2.31) | 0.24 | -- | 1 | 0.89 (0.47, 1.68)^f^ | 0.73 | -- | 11 | 1.38 (0.78, 2.45) | 0.27 |
| Grade 2 | -- | 74 | 1.22 (0.96, 1.56) | 0.10 | -- | 6 |  |  | -- | 68 | 1.27 (0.98, 1.64) | 0.07 |
| Grade 3 | -- | 110 | 1.16 (0.96, 1.40) | 0.12 | -- | 12 | 1.40 (0.74, 2.64) | 0.31 | -- | 98 | 1.13 (0.93, 1.37) | 0.23 |
| Grade unknown | -- | 81 | 1.31 (1.05, 1.64) | 0.02 | -- | 14 | 1.06 (0.65, 1.73) | 0.81 | -- | 67 | 1.42 (1.09, 1.87) | 0.01 |
| Case-only: grade 1+2  vs grade 3^e^ | 86 | 110 | 0.97 (0.73, 1.28) | 0.81 | 7 | 12 | 13.58 (0.64, 287.93) | 0.09 | 79 | 98 | 0.91 (0.68, 1.23) | 0.55 |
| **PRS_ER+_** | | | | | | | | | | | | |
| PRS_ER+_ association with breast cancer risk | | | | | | | | | | | | |
| 1st quartile^b^ | 329 | 46 | 1.00 [reference] |  | 110 | 3 | 1.00 [reference] |  | 219 | 43 | 1.00 [reference] |  |
| 2nd quartile^b^ | 328 | 60 | 1.24 (0.81, 1.90) |  | 83 | 9 | 4.57 (1.10, 19.01) |  | 245 | 51 | 1.04 (0.66, 1.66) |  |
| 3rd quartile^b^ | 328 | 69 | 1.55 (1.02, 2.36) |  | 88 | 11 | 5.33 (1.34, 21.09) |  | 240 | 58 | 1.34 (0.85, 2.10) |  |
| 4th quartile^b^ | 328 | 102 | 2.24 (1.51, 3.31) |  | 99 | 10 | 4.42 (1.13, 17.37) |  | 229 | 92 | 2.11 (1.38, 3.23) |  |
| Continuous^b^ | 1313 | 277 | 1.36 (1.20, 1.55) | <0.001 | 380 | 33 | 1.46 (1.09, 1.94) | 0.01 | 933 | 244 | 1.36 (1.18, 1.57) | <0.001 |
| PRS_ER+_ association with ER-specific breast cancer risk^c^ | | | | | | | | | | | | |
| Controls | 1313 | -- | 1.00 [reference] |  | 380 | -- | 1.00 [reference] |  | 933 | -- | 1.00 [reference] |  |
| ER-negative | -- | 9 | 0.68 (0.40, 1.16) | 0.16 | -- | 2 | 0.27 (0.04, 1.82) | 0.18 | -- | 7 | 0.71 (0.39, 1.30) | 0.27 |
| ER-positive | -- | 199 | 1.37 (1.18, 1.59) | <0.001 | -- | 21 | 1.94 (1.37, 2.76) | <0.001 | -- | 178 | 1.33 (1.14, 1.56) | <0.001 |
| ER-status unknown | -- | 69 | 1.44 (1.16, 1.80) | 0.001 | -- | 10 | 1.05 (0.71, 1.55) | 0.80 | -- | 59 | 1.56 (1.21, 2.02) | <0.001 |
| PRS_ER+_ association with grade-specific breast cancer risk^d^ | | | | | | | | | | | | |
| Controls | 1313 | -- | 1.00 [reference] |  | 380 | -- | 1.00 [reference] |  | 933 | -- | 1.00 [reference] |  |
| Grade 1 | -- | 12 | 1.32 (0.77, 2.26) | 0.32 | -- | 1 | 1.04 (0.65, 1.69)^f^ | 0.86 | -- | 11 | 1.32 (0.74, 2.36) | 0.35 |
| Grade 2 | -- | 74 | 1.28 (1.04, 1.58) | 0.02 | -- | 6 |  |  | -- | 68 | 1.33 (1.07, 1.65) | 0.01 |
| Grade 3 | -- | 110 | 1.29 (1.07, 1.56) | 0.009 | -- | 12 | 1.55 (1.05, 2.29) | 0.03 | -- | 98 | 1.26 (1.02, 1.56) | 0.03 |
| Grade unknown | -- | 81 | 1.54 (1.24, 1.90) | <0.001 | -- | 14 | 1.61 (0.99, 2.63) | 0.06 | -- | 67 | 1.57 (1.23, 2.02) | <0.001 |
| Case-only: grade 1+2  vs grade 3^e^ | 86 | 110 | 1.03 (0.77, 1.37) | 0.87 | 7 | 12 | 14.44 (0.55, 382.09) | 0.11 | 79 | 98 | 0.97 (0.72, 1.31) | 0.85 |

^a^ PRC_BC_ = overall breast cancer PRS; PRS_ER-_ = ER-negative breast cancer PRS; PRS_ER+_ = ER-positive breast cancer PRS; ER = estrogen-receptor. OR = odds ratio per PRS standard deviation, estimated from a multinomial logistic regression (unless otherwise stated); CI = confidence interval. P value was calculated using a 2-sided Wald test.
^b^ PRS quartile thresholds were determined in the combined *BRCA1* and *BRCA2* carrier controls. The continuous test shows the per PRS standard deviation associations, estimated from a multinomial logistic regression model assuming a continuous PRS.
^c^ The ER-specific breast cancer ORs were estimated by partitioning breast cancer status into distinct multinomial outcomes for (i) ER-negative, (ii) ER-positive, or (iii) ER-status unknown.
^d^ The breast cancer grade specific ORs were estimated by partitioning breast cancer status into multinomial outcomes for (i) grade 1, (ii) grade 2, (iii) grade 3, or (iv) grade unknown.
^e^ The case-only breast cancer grade analysis was a logistic regression considering grade 1 and grade 2 breast cancers combined as “controls” and grade 3 breast cancers as “cases”.
^f^ Grade 1 and grade 2 combined for *BRCA1* carriers (to ensure adequate sample size to estimate associations).

**Supplementary Table 10**: Associations of the prostate cancer PRS with prostate cancer risk adjusted for family history of prostate cancer in first- and second-degree relatives.

| PRS investigated and outcome^a^ | ***BRCA1* and *BRCA2* carriers** | | | | ***BRCA1* carriers** | | | | ***BRCA2* carriers** | | | |
| --- | --- | --- | --- | --- | --- | --- | --- | --- | --- | --- | --- | --- |
|  | Controls | Cases | OR (95% CI) | P | Controls | Cases | OR (95% CI) | P | Controls | Cases | OR (95% CI) | P |
| PRS_PC_ association with prostate cancer risk | | | | | | | | | | | | |
| 1st quartile^b^ | 329 | 26 | 1.00 [reference] |  | 88 | 9 | 1.00 [reference] |  | 241 | 17 | 1.00 [reference] |  |
| 2nd quartile^b^ | 328 | 49 | 1.93 (1.15, 3.22) |  | 95 | 15 | 1.53 (0.60, 3.87) |  | 233 | 34 | 2.12 (1.14, 3.94) |  |
| 3rd quartile^b^ | 328 | 54 | 2.08 (1.26, 3.43) |  | 107 | 22 | 1.85 (0.78, 4.35) |  | 221 | 32 | 2.23 (1.20, 4.16) |  |
| 4th quartile^b^ | 328 | 82 | 3.27 (2.01, 5.32) |  | 90 | 24 | 2.88 (1.19, 6.98) |  | 238 | 58 | 3.49 (1.93, 6.30) |  |
| Continuous^b^ | 1313 | 211 | 1.61 (1.38, 1.88) | <0.001 | 380 | 70 | 1.74 (1.29, 2.35) | <0.001 | 933 | 141 | 1.59 (1.32, 1.90) | <0.001 |
| PRS_PC_ association with Gleason score (GS) specific prostate cancer risk^c^ | | | | | | | | | | | | |
| Controls | 1313 | -- | 1.00 [reference] |  | 380 | -- | 1.00 [reference] |  | 933 | -- | 1.00 [reference] |  |
| GS < 7 | -- | 53 | 1.46 (1.10, 1.93) | 0.008 | -- | 26 | 1.11 (0.70, 1.76) | 0.65 | -- | 27 | 1.82 (1.28, 2.60) | <0.001 |
| GS ≥ 7 | -- | 103 | 1.72 (1.39, 2.13) | <0.001 | -- | 21 | 2.06 (1.25, 3.40) | 0.005 | -- | 82 | 1.66 (1.31, 2.11) | <0.001 |
| GS unknown | -- | 55 | 1.59 (1.23, 2.05) | <0.001 | -- | 23 | 2.52 (1.52, 4.18) | <0.001 | -- | 32 | 1.26 (0.95, 1.68) | 0.11 |
| Case-only analysis: GS ≥ 7 vs GS < 7^d^ | 53 | 103 | 1.14 (0.82, 1.59) | 0.43 | 26 | 21 | 1.95 (1.01, 3.75) | 0.05 | 27 | 82 | 0.92 (0.61, 1.37) | 0.67 |

^a^ PRS_PC_ = prostate cancer PRS; GS = Gleason score. OR = odds ratio per PRS standard deviation, estimated from a multinomial logistic regression (unless otherwise stated); CI = confidence interval. P value was calculated using a 2-sided Wald test.

^b^ PRS quartile thresholds were determined in the combined *BRCA1* and *BRCA2* carrier controls. The continuous test shows the per PRS standard deviation associations, estimated from a multinomial logistic regression model assuming a continuous PRS.

^c^ The Gleason score prostate cancer ORs were estimated by partitioning prostate cancer status into distinct multinomial outcomes for (i) GS < 7, (ii) GS ≥ 7, or (iii) GS unknown.

^d^ The case-only prostate cancer analysis was a logistic regression considering GS < 7 prostate cancers as “controls” and GS ≥ 7 prostate cancers as “cases”.

**Supplementary Figures**


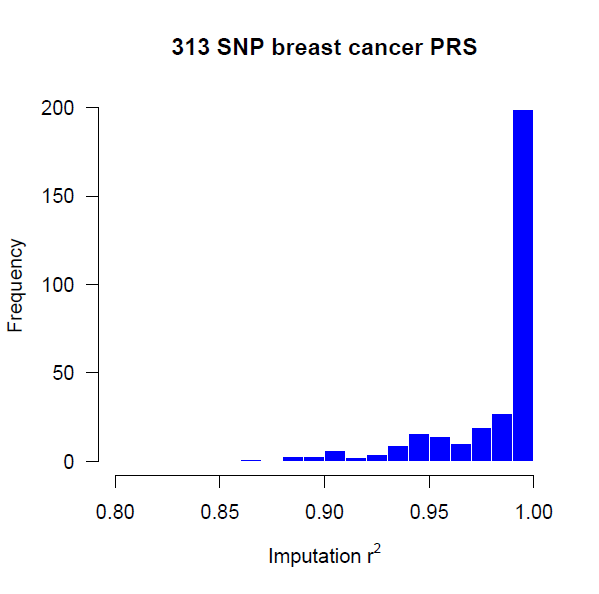


**Supplementary Figure 1:** Histogram of imputation accuracy (*r*^2^ statistics) for the 313 breast cancer polygenic risk score (PRS) single nucleotide polymorphisms (SNPs)^6^. Data were imputed in the combined sample of *BRCA1* and *BRCA2* carriers. All SNPs were well imputed (*r*^2^≥0.86, **Supplementary Table 3**).


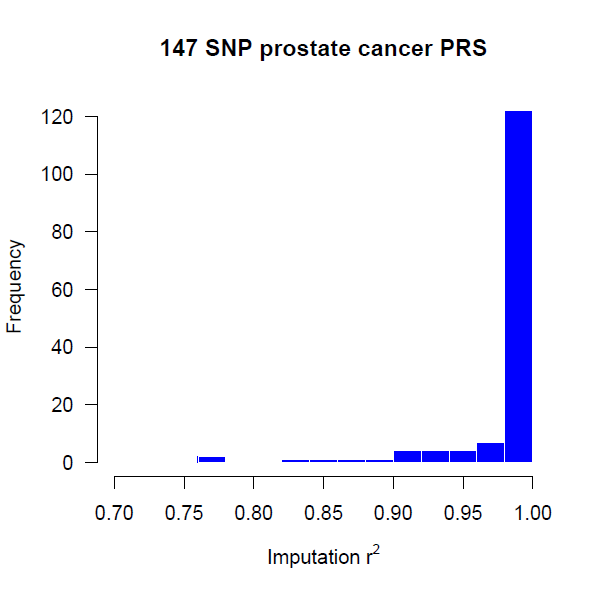


**Supplementary Figure 2:** Histogram of imputation accuracy (*r*^2^ statistics) for the 147 prostate cancer polygenic risk score (PRS) single nucleotide polymorphisms (SNPs)^7^. Data were imputed in the combined sample of *BRCA1* and *BRCA2* carriers. All SNPs were well imputed (*r*^2^≥0.76, **Supplementary Table 4**).


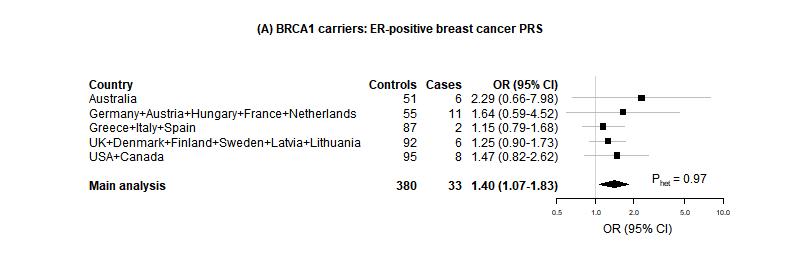

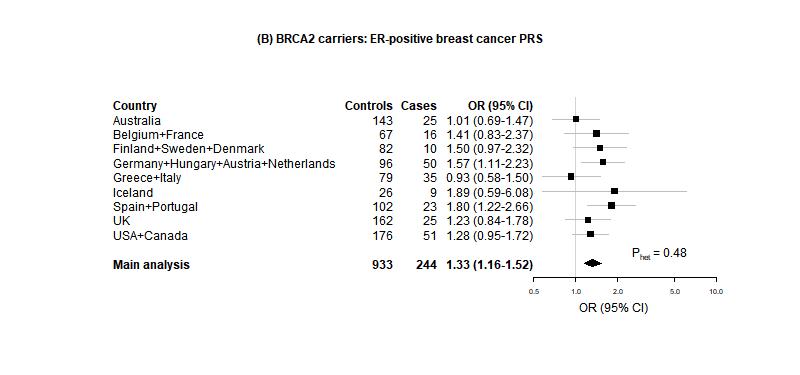


**Supplementary Figure 3:** Forest plots of country specific estrogen receptor (ER)-positive breast cancer polygenic risk score (PRS) associations with breast cancer risk for (**A**) *BRCA1* carriers and (**B**) *BRCA2* carriers. OR = odds ratio per PRS standard deviation, estimated from a multinomial logistic regression; CI = confidence interval; P_het_ = P value testing for heterogeneity of PRS associations across countries. “Main analysis” refers to the model estimating the main effect of the PRS with no interaction term (“continuous” test presented in **Table 1**). All statistical tests were 2-sided.


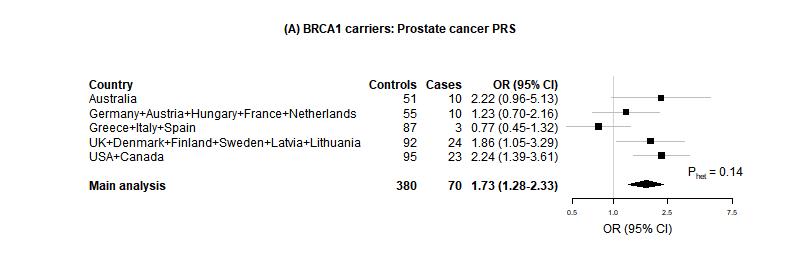

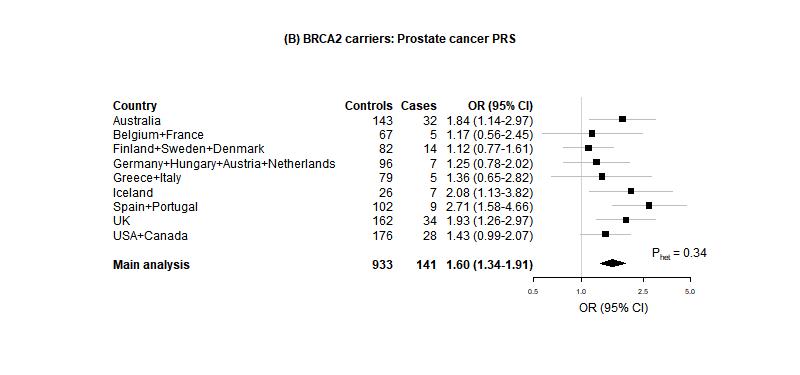


**Supplementary Figure 4:** Forest plots of country specific prostate cancer polygenic risk score (PRS) associations with prostate cancer risk for (**A**) *BRCA1* carriers and (**B**) *BRCA2* carriers. OR = odds ratio per PRS standard deviation, estimated from a multinomial logistic regression; CI = confidence interval; P_het_ = P value testing for heterogeneity of PRS associations across countries. “Main analysis” refers to the model estimating the main effect of the PRS with no interaction term (“continuous” test presented in **Table 2**). All statistical tests were 2-sided.
